# Safety of the fungal workhorses of industrial biotechnology: update on the mycotoxin and secondary metabolite potential of *Aspergillus niger*, *Aspergillus oryzae*, and *Trichoderma reesei*

**DOI:** 10.1007/s00253-018-9354-1

**Published:** 2018-10-06

**Authors:** Jens C. Frisvad, Lars L. H. Møller, Thomas O. Larsen, Ravi Kumar, José Arnau

**Affiliations:** 1 Department of Biotechnology and Biomedicine (DTU Bioengineering), Technical University of Denmark, Søltofts Plads, B. 221, 2800 Kongens Lyngby, Denmark; 2 Department of Product Safety, Novozymes A/S, Krogshoejvej 36, 2880 Bagsvaerd, Denmark; 3 Department of Genomics and Bioinformatics, Novozymes Inc., 1445 Drew Ave., Davis, CA 95618 USA; 4 Department of Fungal Strain Technology and Strain Approval Support, Novozymes A/S, Krogshoejvej 36, 2880 Bagsvaerd, Denmark

**Keywords:** Safety, Mycotoxins, Secondary metabolites, Industrial enzymes

## Abstract

This review presents an update on the current knowledge of the secondary metabolite potential of the major fungal species used in industrial biotechnology, i.e., *Aspergillus niger*, *Aspergillus oryzae*, and *Trichoderma reesei*. These species have a long history of safe use for enzyme production. Like most microorganisms that exist in a challenging environment in nature, these fungi can produce a large variety and number of secondary metabolites. Many of these compounds present several properties that make them attractive for different industrial and medical applications. A description of all known secondary metabolites produced by these species is presented here. Mycotoxins are a very limited group of secondary metabolites that can be produced by fungi and that pose health hazards in humans and other vertebrates when ingested in small amounts. Some mycotoxins are species-specific. Here, we present scientific basis for (1) the definition of mycotoxins including an update on their toxicity and (2) the clarity on misclassification of species and their mycotoxin potential reported in literature, e.g., *A. oryzae* has been wrongly reported as an aflatoxin producer, due to misclassification of *Aspergillus flavus* strains. It is therefore of paramount importance to accurately describe the mycotoxins that can potentially be produced by a fungal species that is to be used as a production organism and to ensure that production strains are not capable of producing mycotoxins during enzyme production. This review is intended as a reference paper for authorities, companies, and researchers dealing with secondary metabolite assessment, risk evaluation for food or feed enzyme production, or considerations on the use of these species as production hosts.

## Introduction

Earlier reviews on the safety of *Aspergillus niger*, *Aspergillus oryzae*, and *Trichoderma reesei* have been published (Schuster et al. 2002; Tanaka et al. 2002; Barbesgaard et al. 1992; Jørgensen 2007; Blumenthal 2004), but since these reviews were written, much progress has been made in the taxonomy, toxicology, natural product chemistry, genomics, genetics, and molecular biology of these fungi.

There is a clear distinction between mycotoxins and other secondary metabolites with attractive properties for diverse applications. Fungal species containing industrial strains have the potential to produce a rather limited number of compounds that are toxic to vertebrates (mycotoxins) and a large variety of other compounds that can display anticarcinogenic or antimicrobial activity, antioxidant activity, be pigments, etc. (Mushtaq et al. 2018). A clear definition of mycotoxin and secondary metabolite is presented here to provide a clear basis for the consideration of safety. The fungal strains that represent the workhorses of industrial biotechnology have a long and extensively documented history of safe use for food and feed applications. Strains belonging to the *Aspergillus* species *A. niger* and *A. oryzae* have been used for fermentation of food for more than 2 millennia and to manufacture food enzymes for over 50 years, while strains of *Trichoderma reesei* have been used safely for decades in enzyme production. Hundreds of enzymes produced in these species are considered as safe by regulatory authorities. Furthermore, mycotoxins and other secondary metabolites are not produced during the controlled, industrially relevant growth conditions where nutrients are not limited and where there is no growth challenge by any other microorganism.

This report includes a comprehensive update of the current knowledge about the mycotoxin and the promising secondary metabolite potential of these industry relevant fungal species. We have considered all published work and have critically evaluated the validity of the data and the accuracy of the taxonomic identification in each case. Consequently, not all publications have been included herein. The report is divided into three sections (taxonomy, mycotoxins, and secondary metabolite potential) for each species.

## Taxonomy of *Aspergillus niger*, *Aspergillus oryzae*, and *Trichoderma reesei*

**Fig. 1:**
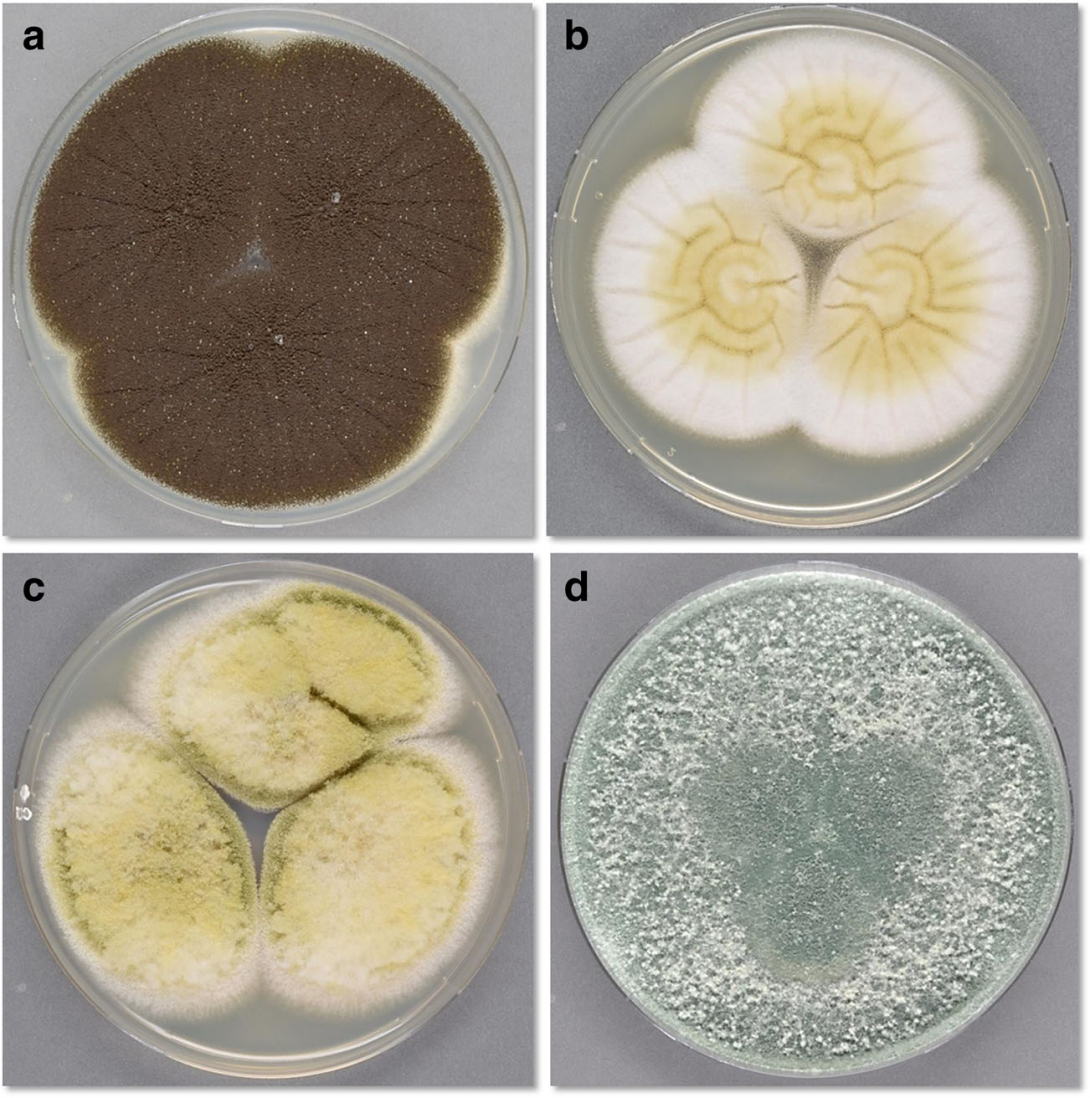
Macroscopic characteristics of 7-day old fungal species growing on solid medium (CYA). **a** *Aspergillus niger*; **b** *A. oryzae*; **c** *A. flavus*; **d** *Trichoderma reesei* (Photo: Birgitte Andersen)

Traditional identification of fungal species relied on microscopic and macroscopic morphological traits, e.g., sporulation structures and other phenotypic features like growth and colony features (see Fig. 1 for examples of *A. niger*, *A. oryzae*, and its close relative *Aspergillus flavus*, together with *T. reesei*). In the last decades, taxonomical classification aided by secondary metabolite profiles has also proven successful (Frisvad and Larsen 2015; Samson et al. 2014). More recently, the use of diagnostic gene sequences like rRNA and, later, the availability of whole genome sequences, have enabled direct comparison of different species at the nucleotide level, throughout the genome. In fact, rDNA-derived ITS sequences are recommended as one of the main “barcodes” for species identification (Samson et al. 2014). However, at least in some *Aspergillus* clades, there is limited variation in, e.g., ITS sequences, requiring the use of additional barcodes like calmodulin or β-tubulin (Samson et al. 2014). The level of resolution of these molecular techniques provides new ways to investigate what defines the species boundaries (Vesth et al. 2018). Still, differences in DNA sequences alone cannot always provide a biological understanding. Also, the profiles of secondary metabolites are species-specific (Frisvad and Larsen 2015) and thereby consistent with phylogenetic relationships in fungi (Larsen et al. 2005; Kocsubé et al. 2016). When taxonomical identification is required, it is therefore advantageous to combine the accumulated knowledge on morphological, physiological, and molecular characteristics. Taxonomical classification of *A. niger*, *A. oryzae*, and *T. reesei* together with relevant related species is described in the following texts.

### *Aspergillus niger*

*Aspergillus niger* is placed within the *Aspergillus niger* clade in the *Aspergillus* section *Nigri* (Varga et al. 2011a). The species is well-circumscribed, but it has a sibling species, with the same properties, called *Aspergillus welwitschiae* (Hong et al. 2013a). The latter species shares all morphological, physiological, and chemical characters with *A. niger* (Fig. 1), and the two species can only be distinguished by sequencing preferably one of the secondary bar-coding genes (Hong et al. 2013b). The DNA barcodes of *Aspergillus welwitschiae* are as follows: ITS (internally transcribed spacer regions and the 5.8 S of the ribosomal gene): FJ629340; BenA (β-tubulin): FJ629291; CaM (calmodulin): KC480196, while *A. niger* has the following barcodes: ITS: EF 661186; BenA (β-tubulin): EF661089; CaM (calmodulin): EF661154; RPB2 (RNA polymerase B2: EF661058). Different strains of *Aspergillus niger* have been genome-sequenced (see Baker 2006; Pel et al. 2007; Andersen et al. 2011).

Other species closely related to *A. niger* are *Aspergillus neoniger*, *Aspergillus tubingensis*, *Aspergillus vadensis*, *Aspergillus luchuensis*, *Aspergillus eucalypticola*, *Aspergillus costaricaensis*, and *Aspergillus piperis*, but it is mostly *A. luchuensis* (formerly *Aspergillus acidus* or *Aspergillus foetidus* var. *acidus*), *A. vadensis*, and *A. tubingensis* that are used in the industry. In some cases, the latter have been misidentified as *A. niger*, and *A. niger* is by far most commonly used species in the industry (Frisvad et al. 2011). *A. luchuensis* is found in fermented Puerh tea (Mogensen et al. 2009) and is used often for koji production (also under the names *Aspergillus kawachii* and *Aspergillus awamori*) (Fujimoto et al. 1993; Hong et al. 2013a; Fujii et al. 2016). *A. niger* sensu stricto is the most commonly used species in biotechnology (Andersen et al. 2011; Frisvad et al. 2011). An often examined typical strain of *A. niger* is ATCC 1015.

Unlike the situation in *A. flavus*, which has a taxonomically accepted domesticated form *A. oryzae*, the domesticated form of *A. niger*, *A. awamori* (Nakazawa 1907, Sakaguchi et al. 1951; Raper and Fennell 1965; Murakami 1979; Al-Musallam 1980), has not been accepted as a valid name, probably because of a mistaken neotypification. Perrone et al. (2011) used the name *A. awamori* for a taxon that was isolated from *Welwitschia mirabilis*, but since the ex-type isolate (CBS 557.65) was not from a koji environment, that species was renamed *A. welwitschiae* by Hong et al. (2013a). Other names such as *Aspergillus usamii* and *A. kawachii* have also been used for domesticated forms of *A. niger* or *A. luchuensis* (Hong et al. 2013b). However, none of these names have been officially taken up for the domesticated form of *A. niger*. The names *Aspergillus phoenicis* and *Aspergillus ficuum* predate *A. niger* and have therefore been rejected, and the name *A. niger* officially conserved because of the economical importance of the latter species (Kozakiewicz et al. 1992).

Average nucleotide identity (ANI) has become the gold standard for taxonomic confirmation of prokaryotes. Two species having > 95% ANI are considered the same species (Rodriguez and Konstantinidis 2014). Although ANI is not widely used in eukaryotes and there are no studies done to layout an ANI-based species framework in fungi, ANI values can still be used to determine the relatedness of two strains or species and can give a better resolution of phylogenetic tree-based inferences (Goris et al. 2007). ANI can also discriminate between closely related populations, and it provides a higher resolution than other sequence analyses, at least in bacteria (Rodriguez and Konstantinidis 2014).

**Fig. 2:**
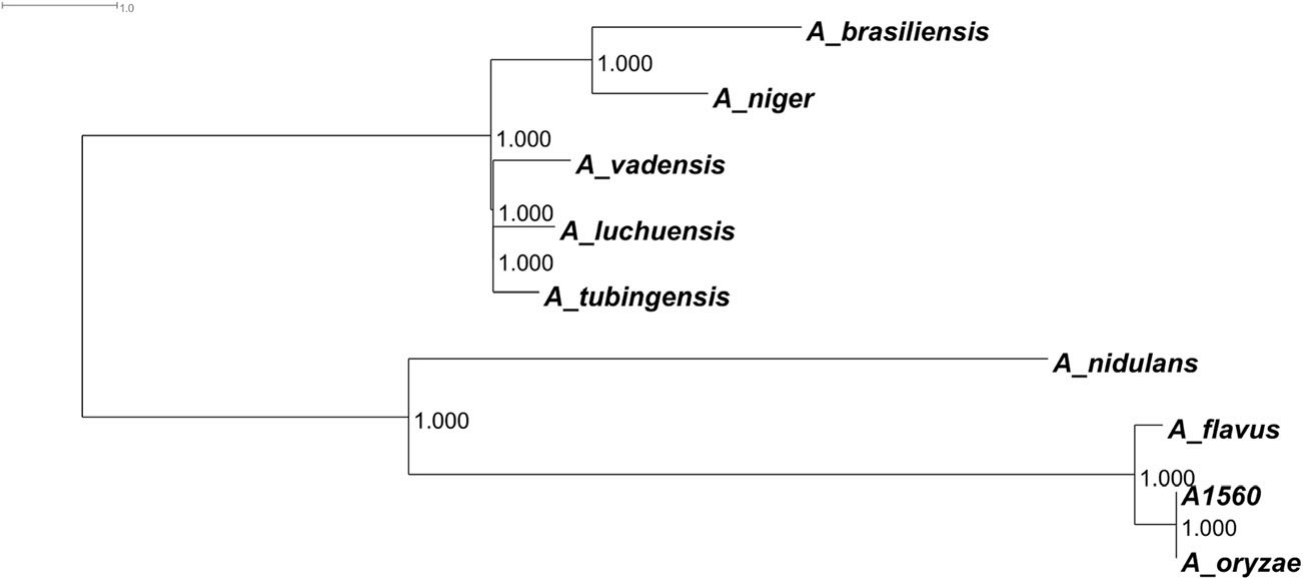
Phylogram based on whole genome sequences of available *Aspergillus* species within the *Nigri* and *Flavi* clades. The phylogram was made using kSNP (version 3.1; Gardner et al. 2015) which computes a core SNP matrix from all the genomes and then executed FastTree (Price et al. 2009) with the maximum likelihood option to compute the tree. The tree was then midpoint-rooted and rendered as a Phylogram using Dendroscope (Huson and Scornavacca 2012)

We performed comparative genomics within species of the *Nigri* clade for which the genome sequence is available using ANI that showed a relatively high identity (85% or higher) between different species in this clade, while a lower level (~ 76%) was obtained when comparing to species outside the clade like *A. oryzae* or *Aspergillus nidulans* (Table 1). Remarkably, a higher ANI was obtained when comparing *A. tubingensis* and *A. luchuensis* (~ 93%) and a slightly lower ANI when comparing *A. tubingensis* with *A. vadensis* or *A. luchuensis* with *A. vadensis* (~ 92%). These three species appear to be more closely related (Table 1), and they all produce asperazines (Nielsen et al. 2009). A phylogenetic tree based on the above-mentioned genome comparison displays the closer relationship between these three species and the clustering of *A. niger* and *Aspergillus brasiliensis* (Fig. 2).

**Table 1:** Reciprocal average nucleotide identity (ANI, Goris et al. 2007) of relevant *Aspergillus* species. A standalone version of the software was downloaded from http://enve-omics.ce.gatech.edu/ani/. Pair-wise comparisons of different combinations were performed using R script and phyton programming

Strain	*A. oryzae* A1560	*A. oryzae* RIB40	*A. flavus* NRRL3357	*A. niger* CBS513.88	*A. brasiliensis* CBS101740	*A. tubingensis* CBS134.48	*A. luchuensis* NBRC 4314	*A. vadensis* CBS 113365	*A. nidulans* FGSCA4
*A. oryzae* A1560	100.00|0.00	99.94|0.85	99.14|1.75	77.77|5.76	77.63|5.22	77.46|5.48	77.24|4.90	77.20|5.08	76.28|4.68
*A. oryzae* RIB40	99.97|0.34	100.00|0.00	99.16|1.61	79.00|7.19	78.09|5.61	77.95|5.64	77.42|5.21	77.60|5.39	76.31|4.72
*A. flavus* NRRL3357	99.18|1.52	99.18|1.49	100.00|0.00	77.44|5.15	77.51|5.02	77.54|5.39	77.41|4.99	77.14|4.98	76.28|4.72
*A. niger* CBS513.88	77.84|6.01	78.35|6.66	77.45|5.31	100.00|0.00	85.67|5.18	86.87|5.23	86.78|5.19	86.89|5.23	77.02|4.95
*A. brasiliensis* CBS101740	77.59|5.09	77.61|5.14	77.49|5.00	85.67|5.18	100.00|0.00	85.18|5.13	85.08|5.10	85.17|5.13	77.16|5.37
*A. tubingensis* CBS134.48	77.43|5.41	77.49|5.40	77.53|5.40	86.85|5.24	85.17|5.14	100.00|0.00	93.25|4.44	92.54|4.76	76.91|4.95
*A. luchuensis* NBRC 4314	77.30|4.90	77.53|5.33	77.38|4.95	86.77|5.19	85.09|5.09	93.28|4.39	100.00|0.00	92.12|4.63	76.85|4.94
*A. vadensis* CBS 113365	77.10|5.04	77.17|5.00	77.12|4.98	86.86|5.26	85.16|5.13	92.53|4.78	92.07|4.72	100.00|0.00	77.04|4.93
*A. nidulans* FGSCA4	76.30|4.73	76.31|4.73	76.25|4.72	77.00|4.97	77.15|5.35	76.84|4.99	76.79|4.95	77.05|4.93	100.00|0.00

Recently, wild-type *A. niger* has been considered as a class 2 microorganism by the German authorities (BAUA see previous texts) because of its potential mycotoxin production and pathogenicity to humans and animals. It is important to discriminate between (1) mycotoxin production as a health hazard during food manufacture and spoilage and corn silage and (2) the growing number of reports of opportunistic pathogens that have resulted in disease, normally in immunocompromised patients. In fact, the baker’s yeast (*Saccharomyces cerevisiae*) can also be considered as a pathogen since it has been associated with disease in severely immunocompromised patients. Perhaps the concept of what constitutes a “pathogen” needs a comprehensive revision and it is not solely related to the taxonomy of the microbe (Casadevall and Pirofski 2003).

### *Aspergillus oryzae*

*A. oryzae* is regarded by most taxonomists as the domesticated form of *A. flavus* (Blochwitz 1929; Wicklow 1984; Klich and Pitt 1988; Georgianna et al. 2009; Rokas 2009; Varga et al. 2011b; Gibbons et al. 2012; Houbraken et al. 2014; Frisvad et al. 2019). Wicklow (1984) claims that domestication (in rice fermentations) has resulted in the following phenotypic differences: conidia in *A. oryzae* are smoother and slightly larger (to adapt to the rice habitat), amylase production is higher, the conidiophore stipes are longer, the mycelium is more floccose, and the conidium color *en masse* is light brownish green rather than yellow grass green as compared to *A. flavus* (Fig. 1). While there are no genotypic differences between *A. oryzae* and *A. flavus* (Thom and Church 1921; Raper and Fennell 1965; Murakami 1971; Christensen 1981; Pitt et al. 1983; Wicklow 1984; Klich and Pitt 1985, 1988; Geiser et al. 1998, 2000; Gibbons et al. 2012; Powell et al. 2008; Varga et al. 2011; Gilbert et al. 2018; Frisvad et al. 2019), there are several morphological and physiological differences between the two species as listed previously. Furthermore *Aspergillus oryzae* cannot produce aflatoxins, aspergillic acid, and flavimine, that are otherwise present in most strains of *Aspergillus flavus* (Thom and Church 1921; Raper and Fennell 1965; Murakami 1971; Christensen 1981; Wicklow 1984; Klich and Pitt 1985, 1988; Pitt et al. 1983; Varga et al. 2011; Frisvad et al. 2018; Fig. 1). Klich and Mullaney (1987) were able to distinguish between strains of *A. oryzae* and *A. flavus* by DNA restriction enzyme fragment polymorphisms. Nearly all strains of *A. flavus* produce a bright orange reverse on the medium AFPA (*Aspergillus flavus parasiticus* agar), while *A. oryzae* strains produce a cream-colored reverse (Bothast and Fennell 1974; Hamsa and Ayres 1977; Pitt et al. 1983).

In accordance with this, genome sequencing of *A. flavus* (Nierman et al. 2015; Faustinelli et al. 2016) and strains of *A. oryzae* (Machida et al. 2005; Galagan et al. 2005; Umemura et al. 2012, 2013a,b; Zhao et al. 2012, 2013a,b, 2014a,b) have shown that these two species are very similar. Interestingly, the first sequenced strain of *A. oryzae* may be an *A. flavus* “sensu stricto.” The isolate RIB40 produces large globose sclerotia (Rank et al. 2012; Fig. 1) and was isolated from a broad bean, Kuriyamacho, Kyoto, Japan, in a field, not from a fermentation factory. Based on the first identification as *A*. *oryzae* var. *brunneus*, it has brownish conidia and, therefore, resemble *A. oryzae*. RIB40 does not produce aflatoxin as it contains disabling mutations in the gene cluster (Tominaga et al. 2006). It has been shown that *A. flavus* isolates gradually lose their ability to produce spores, sclerotia, and aflatoxin-producing capability after several serial transfers (Torres et al. 1980; Horn and Dorner 2001; Chang et al. 2007). The production of large globose sclerotia is characteristic for *A. flavus* sensu stricto (Geiser et al. 2000), and only few strains of *A. flavus* (for example NRRL 3251) produce small sclerotia (Hesseltine et al. 1970; Saito and Tsuruta 1993), while other strains with small sclerotia belong to the species *Aspergillus minisclerotigenes*, *Aspergillus aflatoxiformans*, *Aspergillus austwickii*, and *Aspergillus cerealis* (Varga et al. 2011; Frisvad et al. 2018). Overall, none of the characterized true *A. oryzae* isolates produce aflatoxins. For *A. flavus*, the situation is more complex since some isolates, including the ex-type strain (NRRL1957), do not produce aflatoxin. However, aflatoxin production has been shown for a large number of *A. flavus* including NRRL3357.

Genome sequencing has allowed several comparative studies to be carried out (Abe et al. 2006; Payne et al. 2006; Kobayashi et al. 2007; Rokas et al. 2007; Machida et al. 2008). *A. oryzae* is used extensively in enzyme production at industrial scale (Barbesgaard et al. 1992; Jørgensen 2007) and as a successful expression host for production of secondary metabolites (Sakai et al. 2008; Liu et al. 2015; Minami et al. 2016; He et al. 2018). In practice, sequence barcodes for *A. oryzae* include the following: (1) ITS (accession no. EF661560); (2) BenA (β-tubulin, accession no. EF661483); (3) CaM (calmodulin accession no. EF661506); and (4) RPB2 (RNA polymerase B2, accession no. EF661438) and for *A. flavus*: (1) ITS: (AF027863); (2) BenA (EF661485); (3) CaM (EF661508); and RPB2 (EF661440). Remarkably, the barcodes are not sufficient to effectively separate *A. flavus* and *A. oryzae*. More elaborate molecular techniques are required to distinguish these species (Godet and Munaut 2010). ANI analysis showed a very high degree of sequence homology, well above 99%, between RIB40 and other *A. oryzae* strains used in industrial enzyme production like A1560 (synonym IFO 4177), while a slightly lower percentage is observed when comparing *A. flavus* and *A. oryzae* (Table 1). The use of the % identity between *A. oryzae* RIB40, A1560 or *A. flavus* NRRL3357 (99.9% versus 99.1%) does not allow a direct species discrimination based on ANI. Furthermore, ANI between *A. oryzae* and species from the *Nigri* section display an ANI value below 80%. Members of the *Nigri* section display an ANI of 85% or higher. Lower ANI (approx. 75%) is obtained when comparing either *A. oryzae*/*A. flavus* to *A. nidulans* or species from the *Nigri* section to *A. nidulans*. Overall, as in the case of *A. niger*, the above-mentioned data demonstrate that genome homology data alone cannot be used for taxonomical purposes and need to be complemented by phenotypic properties.

### *Trichoderma reesei*

*Trichoderma reesei* (anamorph) has also been named *Hypocrea jecorina* (teleomorph and holomorph), but with the new nomenclatural system used after 2011, *Trichoderma reesei* is considered the correct name for this fungus (Samuels et al. 1998; Samuels et al. 2012, Fig. 1). Most of the industrial strains have a single common ancestor, RUT-C30, which displays a blue-green color on solid medium (Fig. 1). The genome sequence has also been reported for this species (Martinez et al. 2008). The *T. reesei* type strain is QM6a.

## Mycotoxins are a very limited group of fungal secondary metabolites

Fungal secondary metabolites can be defined as outward-directed, small differentiational molecules of restricted taxonomic distribution that are genetically encoded by clustered genes and accumulated and normally secreted. Secondary metabolites are a very heterogeneous chemical group of low molecular weight compounds that include antimicrobials, antioxidants, pigments, hormones, and metal chelators. A great number of these compounds have therefore a very significant potential application.

In general, any competition-selected fungal species has the potential to produce hundreds of individual secondary metabolites coded by up to 90 biosynthetic gene clusters (Clevenger et al. 2017; Lind et al. 2017). The major biosynthetic classes of secondary metabolites are polyketides, non-ribosomal peptides, terpenes, and shikimic acid-derived compounds, but many compounds are hybrids of these classes. The genes coding for the enzymes involved in the biosynthesis of these compounds are associated in gene clusters. The genomes of *A. niger*, *A. oryzae*, and *T. reesei* include 78, 75, and 27 gene clusters for secondary metabolite biosynthesis, respectively (Lind et al. 2015; Zeilinger et al. 2016; Wasil et al. 2018), although these numbers may vary depending on the strain and the software package used. Furthermore, each biosynthetic gene cluster may be responsible for the production of a large number of precursors, shunt products, and final products. For example *Aspergillus oryzae* was reported to produce many members (26) of the cyclopiazonic acid biosynthetic family of compounds (Liu et al. 2018), including cyclopazonic acids, speradines, cyclopiamides, and asporydines. With the development of new genome mining approaches (Kjærbølling et al. 2018) and algorithms such as antiSMASH (Blin et al. 2017), the prediction of secondary metabolite encoding gene clusters has become easier. On the other hand, the chemical modifications based on important accessory tailoring genes on the core structure of secondary metabolites may be more difficult to predict from sequences (Bertrand et al. 2018) and often require full structure elucidation. In this context, it is important to note that majority of gene clusters are not expressed under standard cultivation and that no fungal species synthesizes all potential secondary metabolites at any given time. As mentioned previously, production of secondary metabolites does not normally occur under production-relevant growth conditions where no species competition or nutrient starvation threat is used.

Mycotoxins are a very limited group of fungal secondary metabolites. Regarding biotechnology, mycotoxins are important if they pose a safety concern in the industrial application of fungi for enzyme or bulk metabolite production as well as in other areas like food spoilage and in building environments. There have been numerous definitions of the word mycotoxin (Bennett and Inamdar 2015; Taevernier et al. 2016), but a strict consensus definition that we endorse is the following: Mycotoxins are secondary metabolites genetically encoded by clustered genes and produced by fungi. These mycotoxins are acutely or chronically toxic and pose health hazards or death in humans and other vertebrates when acquired in small amounts via a natural route (orally, by inhalation, or via the skin). This definition is a combination of that of Jarvis and Miller (2005), Frisvad (2011), Bennett and Inamdar (2015), and Taevernier et al. (2016). Taevernier et al. (2016) suggested that a quantitative level of cell cytotoxicity on preferably human cell cultures with an IC_50_ (the concentration required for 50% of cell viability) of less than 1000 μM could be used to determine whether a fungal secondary metabolite was considered a mycotoxin or not. We cannot accept this definition as such molecules may be cytotoxic, while not necessarily being toxic when acquired via a natural route. Earlier claims of mycotoxicity were based on other toxicity data, such as toxicity including cancerogenicity after intraperitoneal or subcutaneous injection (Dickens and Jones 1961; Cole and Cox 1981; Lu et al. 2017), but this too is not a natural route of intake. For example, patulin and penicillic acid were originally claimed to be cancerogenic based on subcutaneous injection (Dickens and Jones 1961), but Enomoto and Saito (1972) rightly mention that experimental production of cancer should be confirmed in animals by oral administration of mycotoxin.

The safe use of fungal strains is recognized in official classifications of biological agents into risk groups; e.g., BAUA (German Federal Institute for Occupational Safety and Health) classifies *A. niger* and *A. oryzae* as risk group 2 biological agents. Importantly, BAUA recognizes that strains belonging to these species may still be classified as risk group 1 biological agents if documentation of safety and/or history of safe use is provided.

In the following sections, we describe the mycotoxins that are potentially produced by the three industrial organisms and relevant related species. Only mycotoxins with a documented effect are described. All other secondary metabolites are described in the section on secondary metabolite potential and are not considered mycotoxins according to the definition herein.

### Mycotoxins potentially produced by *Aspergillus niger*

*Aspergillus niger* has been claimed to produce a very large number of mycotoxins and other secondary metabolites (Table 2; Nielsen et al. 2009). Apart from a large number of volatiles and small organic acids (Wani et al. 2010; Priegnitz et al. 2015; Costa et al. 2016), *A. niger* sensu stricto can produce very few mycotoxins but a large number of other secondary metabolites. In many cases, fungi identified as *A. niger* were indeed *A. tubingensis* or other closely related species (Table 2).

**Table 2:** Mycotoxins from *Aspergillus niger* (and its sibling species *A. welwitschiae*) (Nielsen et al. 2009)

Mycotoxin	Reference	Comment
Fumonisin B_2_	Frisvad et al. (2007, 2011)	This mycotoxin has been found in more than 75% of strains examined of *A. niger* (Frisvad et al. 2011)
Fumonisin B_4_	Mogensen et al. (2010); Månsson et al. (2010)	
Fumonisin B_6_	Månsson et al. (2010)	
Ochratoxin A	Abarca et al. (1994)	This mycotoxins has been found in less than 10% of the strains of *A. niger* examined (Frisvad et al. 2011)
Oxalic acid	Raistrick and Clark (1919); Yassin et al. (2015)	Nearly all strains of *A. niger* produce oxalic acid

### Fumonisins

Fumonisins are strongly reduced polyketides with two added tricaballyllic acid groups and an amino group added from a non-ribosomal peptide. They are mycotoxins associated with multiple human and animal diseases, as they are produced in large amounts in cereals by common *Fusarium* species (Braun and Wink 2018; Cendoya et al. 2018). Fumonisins induce leukoencephalomalacia in horses, nephro- and hepato-toxicity in rodents, and pulmonary toxicity in pigs, and they have been classified as International Agency for Research on Cancer (IARC) type 2B carcinogens in humans (esophageal cancer) (Cendoya et al. 2018). However, *Aspergillus niger* and its sibling species *A. welwitschiae* (originally named *A. awamori*) also produce fumonisins of the B_2_, B_4_, and B_6_ types (Frisvad et al. 2007; 2010) and may produce fumonisins in cereals and grapes (Logrieco et al. 2009; Mogensen et al. 2010; Munkvold et al. 2018). Several industrial strains have the capability to produce fumonisins (Frisvad et al. 2010; Han et al. 2017), so it is important to use strains that do not produce these mycotoxins. Current *A. niger* production strains have been developed that either have been selected due to the lack of fumonisin production or contain a deletion of the fumonisin gene cluster (unpublished results).

The impact of fumonisins on human health remains poorly understood (Voss and Riley 2013). It has been known for long that fumonisins are hepatotoxic, nephrotoxic, atherogenic (induces formation of plaque in arteries), immunosuppressive, and embryotoxic in experimental animal systems (Nair 1998). Structurally, fumonisin B1 shows similarity to the cellular sphingolipids, and this similarity has been shown to disturb the metabolism of sphingolipids leading to accumulation of sphinganine in cells and tissues. The cellular mechanisms behind fumonisin B_1_-induced toxicity include the induction of oxidative stress, apoptosis, and cytotoxicity, as well as alterations in cytokine expression (Stockmann-Juvala and Savolainen 2008). Mechanistically, the toxicity of fumonisin B_2_ and B_3_ is relatively poorly understood, but a comparison of the toxicities of fumonisin B_1_, B_2_, and B_3_ individually and in combination has shown that all three are toxic, but with fumonisin B_1_ being the most toxic of the three (Henry and Wyatt 2001).

### Ochratoxin

Ochratoxin A (OTA) is a mycotoxin that is a common contaminant of a wide variety of food products. The molecular structure comprises a chlorinated polyketide dihydroisocoumarin ring linked to phenylalanine and, as shown in different producing fungal species, a polyketide synthase (PKS) is a major part of the biosynthetic pathway (Wang et al. 2016; Massi et al. 2016; Gallo et al. 2017; Gill-Serna et al. 2018). OTA inhibits protein synthesis and energy production, induces oxidative stress, cell apoptosis and necrosis, and DNA adduct formation, and is mostly recognized as a nephrotoxin (Heussner and Bingle 2015; Közégi and Poór 2016). It is classified as an IARC type B2 carcinogen in human beings.

### Oxalic acid

Oxalic acid is a strong dicarboxylic acid. Oxalic acid is a reducing agent and its conjugate base, known as oxalate, is a chelating agent for metal cations. Typically, oxalic acid occurs as the dihydrate. Excessive ingestion of oxalic acid or prolonged skin contact can be dangerous. Oxalic acid is hepatotoxic, but it will only have a negative effect in quite high doses (Jahn 1977). *Aspergillus niger* infections are often accompanied with oxalosis (Kredics et al. 2008; Oda et al. 2013), and in one case, calcium oxalate produced by *A. niger* in the lungs caused hyperoxaluria in the kidneys (Vaideeswar and Sakhdeo 2009), so both kidneys and the liver can be affected. However, such cases are rare and will only happen in severely immunocompromised patients. Otomycoses are often caused by *Aspergillus tubingensis* rather than *A. niger* (Kredics et al. 2008).

### Improved safety of *A. niger* industrial strains

As mentioned previously, *A. niger* has the potential to produce ochratoxin, fumonisin, and oxalic acid. Industrial strains have been developed by classical mutagenesis and by deletion of the genes involved in the biosynthesis (Susca et al. 2014).

### Mycotoxins from *Aspergillus oryzae* and *A. flavus*

*A. oryzae* and its closely related species *A. flavus* can produce a very limited number of mycotoxins (Table 3). Their macroscopic similarity has contributed to a disparity of reports on the potential production of mycotoxins from either species. Mycotoxins produced by these species are described in the following texts with attention to knowledge about the potential production of these compounds by either species and reports that describe production in wrongly assigned species.

**Table 3:** Mycotoxins reported from *Aspergillus flavus* and its domesticated form *A. oryzae*

Metabolite	Reference	Comment
Aflatoxins B_1_, B_2_, B_2α_, B_3_ and precursors Aflatoxins G_1_ and G_2_ have been found in few strains of *A. flavus* from South Korea.	Hartley et al. (1963); Asao et al. (1963); van der Merwe et al. (1963); van Dorp et al. (1963); Asao et al. 1965; Burkhart and Forgacs 1968; Dutton and Heathcote (1968); Rodricks et al. (1968); Waiss et al. (1968); Heathcote and Dutton (1969); Holker et al. (1966); Cole et al. (1970); Schroeder and Kelton (1975); Frisvad et al. (2019); Rodríguez et al. (2012)	Only found in some strains of *Aspergillus flavus* and never found in *A. oryzae*
Cyclopiazonic acid and iso-α-cyclopiazonic acid, β-cyclopiazonic acid (= bissecodehydrocyclopiazonic acid), α-cyclopiazonic acid imine, 2-oxocyclopiazonic acid, cyclopiamide A, cyclopiamide E & H, speradine A, B, C, D, E, F, H, I, 3-hydroxy-speradine A, cAATrp, and asperorydine A-M	Ohmomo et al. (1973) (misidentified as *A. versicolor*; Domsch et al. 2007); Luk et al. (1977); Orth (1977); Gallagher et al. (1978); Tokuoda et al. (2008); Hu et al. (2014a,b); Ma et al. (2015); Uka et al. (2017); Liu et al. (2018)	Cyclopiazonic acid has been found in several strains of both *A. flavus* and *A. oryzae*
β-Nitropropionic acid	Bush et al. (1951); Nakamura and Shimoda (1954); Iwasaki and Kosikowski (1973); Orth (1977); He et al. (2016)	Found in some strains of both *A. oryzae* and *A. flavus*

*A. oryzae* is a domesticated species originating probably from *Aspergillus flavus*, and the two species can not be distinguished by DNA sequence differences. Since *A. oryzae* is domesticated, it can only be expected to be found in fermentation environments. Any *A. oryzae* recovered in nature can only be found there if it has escaped such a fermentation plant, and based on its adaptation to the fermentation environment, it must be expected to be a poor competitor in cereals, oilseeds, and nuts, where *A. flavus* is a very competitive species (Wicklow 1984).

### Aflatoxins

The aflatoxins (B_1_ and B_2_ primarily) are polyketides that have been found in many strains of *Aspergillus flavus*, albeit not the culture ex type of *A. flavus* (Varga et al. 2009). Aflatoxin (AFL) has been reported from strains of *Aspergillus oryzae*, but these data are based on misidentified strains or misidentified mycotoxins or contaminated cultures (Varga et al. 2009). It has been shown that strains of *Aspergillus oryzae* sensu stricto cannot produce AFL, as a result of the lack of essential parts of the gene cluster, e.g., deletion of the *aflR* gene involved in induction of biosynthesis (Cary and Ehrlich 2006; Chang et al. 2007; Lee et al. 2006a,b; Tominaga et al. 2006; Takahashi et al. 2008, 2012; Kiyota et al. 2011; Hong et al. 2013b; Lee et al. 2014; Tao and Chung 2014). Therefore, AFL production can be excluded in *A. oryzae* sensu stricto. Furthermore, current industrial strains contain a deletion of the whole AFL gene cluster, providing additional safety in enzyme production. Type G aflatoxins (aflatoxin G_1_ and G_2_) have rarely been reported from *Aspergillus flavus*. In some cases, the G type aflatoxins were produced by *A. parasiticus* and other species from section *Flavi* (Varga et al. 2011), rather than isolates that confidently can be allocated to *A. flavus* sensu stricto. Saldan et al. (2018) reported on aflatoxin G_1_ production by *A. flavus* ATCC 9643, but this strain may not be an *A. flavus* sensu stricto. Five Korean strains of *A. flavus* sensu stricto were reported to produce the G-type aflatoxins (Frisvad et al. 2019).

### Cyclopiazonic acids

Cyclopiazonic acid (= α-cyclopiazonic acid) (CPA) is an indol tetrameric acid, hybrid polyketide/non-ribosomal peptide/DMAT (dimethylallyl terpene unit) compound that was isolated from *A. flavus* originally by Luk et al. (1977) and Gallagher et al. (1978) but has since been found repeatedly in *A. flavus* (Varga et al. 2011b). It was originally isolated from a fungus identified as *Penicillium cyclopium*, but the strains of *Penicillium*-producing cyclopiazonic acid were *Penicillium griseofulvum* and *Penicillium commune* (Frisvad 1989; Frisvad et al. 2004). CPA has also been isolated repeatedly from *Aspergillus oryzae* (Orth 1977; Ohmomo et al. 1973, erroneously reported as *A. versicolor*; see Domsch et al. 2007; Frisvad 1989; Tokuoda et al. 2008; Shaaban et al. 2014). It is possible to remove the CPA gene cluster and thus avoid CPA production in biotechnological processes (Kato et al. 2011). *A. oryzae* and *A. flavus* can produce a large number of secondary metabolites related to CPA including iso-α-cyclopiazonic acid, β-cyclopiazonic acid (= bissecodehydrocyclopiazonic acid), α-cyclopiazonic acid imine, 2-oxocyclopiazonic acid, cyclopiamide (A), cyclopiamide E & H, speradine A, B, C, D, E, F, H, I, 3-hydroxy-speradine A, cAATrp, and asperorydine A-M (Ohmomo et al. 1973; Holzapfel et al. 1990; Hu et al. 2014a,b; Ma et al. 2015; Tokuoka et al. 2015; Xu et al. 2015; Uka et al. 2017; Liu et al. 2018) from *A. oryzae* and *A. flavus*, but speradine A is also produced by *Aspergillus tamarii* (Tsuda et al. 2003). Some of the strains reported as *A. oryzae* producing these tetramic acids have been isolated from marine sources, so they may in fact be *A. flavus.* However, the speradines are related to CPA, produced by many strains of both *A. flavus* and *A. oryzae*, and so speradines are not unlikely secondary metabolites in *A. oryzae*. There have been some problems with the naming of speradine B that is a different speradine in *Penicillium dipodomyicola* (Wang et al. 2015) than that from *A. flavus*, so some of the speradines need to be renamed.

### β-nitropropionic acid

β-nitropropionic acid (BNP) is one of the real mycotoxins reported from authentic *Aspergillus oryzae* strains, but also from *A. flavus* strains (Bush et al. 1951; Nakamura and Shimoda 1954; Iwasaki and Kosikowski 1973; Orth 1977). It has caused sugarcane disease in children eating sugarcane infected with *Nigrospora* spp. that produce β-nitropropionic acid also (Liu et al. 1989; Ming 1995; Fu et al. 1995; Johnson et al. 2000; Fernagut et al. 2002; He et al. 1995). The genetic basis for production of BNP is not completely understood. Therefore, BNP levels are monitored in industrial enzyme productions.

### Mycotoxins from *Trichoderma reesei*

It seems that chemotaxonomy is working excellently at the species level in *Trichoderma* (Kang et al. 2011). In the latter paper, *T. reesei* was not included, and it is only few mycotoxins that are ascribed to *T. reesei* (Zeilinger et al. 2016) (Table 4). Reported mycotoxins from *T. reesei* (claimed to be a mutant of QM 9414 and called P-12) include trichodermin (Watts et al. 1988), but this ability to produce trichodermin by *T. reesei* has been rejected by Nielsen et al. (2005). The latter authors claimed that only *Trichoderma brevicompactum* can produce trichodermin, and possibly also *Trichoderma arundinaceum* (Zeilinger et al. 2016). There are also some trichothecene genes in *Trichoderma gamsii* and *Trichoderma asperellum*, but such genes have not been observed in *T. reesei* (Zeilinger et al. 2016). Also, the mycotoxin gliotoxin has been mentioned as a potential secondary metabolite in *Trichoderma*, because a gene cluster seems to be present in the genome of this fungus (Zeilinger et al. 2016). However, gliotoxin has never been detected in any culture of *T. reesei* (Martinez et al. 2008; Kubicek and Druzhinina 2016). *T. reesei* thus seems to be unable to produce mycotoxins.

**Table 4:** Mycotoxins reported from *Trichoderma reesei*

Metabolite	Reference	Comment
Gliotoxin	Zeilinger et al. (2016)	Actual gliotoxin production was not shown
Trichodermin	Watts et al. (1988); Nielsen et al. (2005)	Culture could have been contaminated, but claimed to be derived from QM 9414 as strain P-12; the claim that *T. reesei* produces this mycotoxin may also be based on insufficient analytical chemical methods

### Toxicity of fungal mycotoxins relevant for *A. niger*, *A. oryzae*, and *T. reesei*

Mycotoxins often affect different vertebrate species very differently. However, to enable a comparison of the relative toxicity of the mycotoxins potentially produced by *A. niger*, *A. oryzae*, and *T. reesei*, an overview of acute oral toxicity is provided (Table 5). In the enzyme industry, it is ensured that production strains based on *A. niger*, *A. oryzae*, and *T. reesei* do not produce mycotoxins when grown at large scale.

**Table 5:** Acute oral toxicity of mycotoxins potentially expressed by *Aspergillus niger*, *A. oryzae*, and *Trichoderma reesei*

Species	Metabolite	LD50 (acute oral toxicity, mg/kg)		Reference
		Rat	Mouse	
*Aspergillus niger*	Fumonisin B2	> 46.4^a^	–	McKean et al. (2006)
	Fumonisin B4			
	Fumonisin B6			
	Oxalic acid	375	–	Vernot et al. (1977)
	Ochratoxin A	20	46	Purchase and Theron (1968) Kayoko (1985)
*Aspergillus oryzae*	Cyclopiazonic acid	36	13	Purchase (1971); Nishie et al. (1985)
	ß-Nitropropionic acid	60	68	Burdock et al. (2001) Blumenthal et al. (2004)
	Kojic acid^b^	1800	5100	SCCS (2012)

^a^The listed value is for fumonisin B1 as the exact values for B2, B4, and B6 have not been determined

^b^Kojic acid is not a mycotoxin and is included for comparison purposes only

As shown previously, *A. niger* can produce the mycotoxins ochratoxin A, fumonisins B_2_, B_4_, and B_6_, and oxalic acid, and *A. oryzae* can produce the mycotoxins cyclopiazonic acid and β-nitropropionic acid, and *T. reesei* has not been convincingly shown to produce any mycotoxins.

### Improved safety of *A. oryzae* industrial strains

As mentioned previously, *A. oryzae* strains are not able to produce aflatoxins due to the presence of disabling mutations in the gene cluster. Modern industrial strains have been developed that contain a large DNA deletion. This region includes the aflatoxin gene cluster and genes involved in the biosynthesis of cyclopiazonic acid (CPA, Christensen et al. 2000). Thus, during industrial enzyme production using strains derived from A1560 containing the chromosomal deletion, the presence of neither aflatoxin nor CPA is a concern.

## Secondary metabolite potential

Fungal secondary metabolites are very diverse and include compounds with a wide range of applications (e.g., antibiotics, cancer treatment, immunosuppressing drugs, pigments, antioxidants).

Like many other fungi, *Aspergillus* species are capable of producing a very large number of drugs and drug-lead compounds. Among the best known for medical applications are the antibiotic penicillin to combat bacterial infections, the cholesterol-lowering mevinolin from *Aspergillus terreus*, the anticancer compound fumagillin from *Aspergillus fumigatus*, the antifungal echinocandin from *Aspergillus pachycristatus* and mulundocandin from *Aspergillus mulundensis* (Baltz et al. 2010; Houbraken et al. 2014; Zeiliger et al. 2015; Bills et al. 2016; Park et al. 2017a).

Fungi produce a large number of other secondary metabolites. Among them, fungal pigments such as polyketide-derived azaphilones are used to add color and as antioxidants in food. *Aspergillus* species are used to produce yellow and brown pigments like fumigatin (Hanson 2008). Additionally, red pigments have been reported in, e.g., an *A. flavus* strain (Gurupavithra et al. 2017). Carotenes are important terpenoid pigments and antioxidants that are produced in many bacteria, fungi, algae, and plants. Interestingly, carotene is produced by few *Aspergillus* species and not by *Trichoderma reesei* (Avalos and Limon 2015).

### Secondary metabolites described in *A. niger*

*Aspergillus niger* has been claimed to produce a very large number of secondary metabolites (Table 6; Nielsen et al. 2009) including isoflavones which are actually plant metabolites (Umezawa et al. 1975; Nielsen et al. 2009). Apart from many volatiles and small organic acids (Wani et al. 2010; Priegnitz et al. 2015; Costa et al. 2016), *A. niger* sensu stricto can produce a variety of other secondary metabolites. In many cases, fungi identified as *A. niger* were indeed *A. tubingensis* or other closely related species (Table 2).

**Table 6:** Secondary metabolites reported from *A. niger* and closely related species

Secondary metabolite	Reference	Comment
Small acids: Glyoxylic acid, glycolic acid, hydropyruvic acid, parasorbic acid, sorbic acid, ascorbic acid, fumaric acid, gluconic acid, citric acid, glutaric acid, phenylacetic acid, phenoxyacetic acid, p-methoxyphenylacetic acid, 4-hydroxymandelic acid, D-galactonic acid	Nielsen et al. (2009); Cairns et al. (2018)	
Anominine and other aflavinines	Frisvad et al. (2014)	Found in sclerotia of *A. niger*
Asperamide A & B	Zhang et al. (2007a,b,c,d)	
Asperenone	Jefferson (1967a,b); Yu et al. (1967); Pattenden (1969, 1970); Rabache et al. (1974); Rao et al. (2002)	Also referred to as asperyellone and asperrubrol
Aspergetide	Lee et al. (2015)	
Aspergillin	Ray and Eakin (1975)	
Aspernigrin A, B, C, D	Hiort et al. (2004); Ye et al. (2005); Zhou et al. (2016)	
Azanigerones A-F	Zabala et al. (2012)	
Bicoumanigrin	Hiort et al. (2004)	
Carlosic acid, agglomerin F, carlosic acid methyl ester	Yang et al. (2014)	Produced by *A. brasiliensis*
Chlovalicin	Uchoa et al. (2017)	Identity of producer not convincingly confirmed
Cycloleucomelon and atromentin	Hiort et al. (2004)	
Cyclo (trans-4-hydroxy-L-Pro-L-Leu), cyclo (L-Pro-L-Phe), cyclo (trans-4-hydroxy-L-Pro-L-Phe, Cyclo (L-Pro-L-Tyr), cyclo (L.Pro-L-val), cyclo (L-Pro-L-Leu)	Uchoa et al. (2017)	Identity of producer not convincingly confirmed
Funalenone	Inokoshi et al. (1999)	
Gibberellic acid, gibberellin, indoleacetic acid	Cihangir (2002); Ates et al. (2006); Lubna et al. (2018)	Producer is probably *A. tubingensis*
JBIR-86 and JBIR-87	Takagi et al. (2010); Henrikson et al. (2011)	
Malformin A1, A2, B1, B2, B3, B5, C	Curtis and Tanaka (1967); Yoshizawa et al. (1975); Sugawara et al. (1990); Kim et al. (1993); Zhou et al. (2016); Uchoa et al. (2017)	
Kotanin, desmethylkotanin, orlandin	Cutler et al. (1979); Sørensen et al. (2009); Hüttel and Müller (2007); Girol et al. (2012); Mazzaferro et al. (2015)	
Maltoryzin	Abdelghany et al. (2017)	Identity of producer not convincingly confirmed
4-Methoxybenzyl-7-phenylacetamido-3-vinyl-3-cephem-4-carboxylate	Bandara et al. (2015)	
Nafuredin	Ui et al. (2001)	
Naphtho-γ-pyrones (asperpyrone A-E, aurasperone A-H, 10,10′-bifonsecin, 6’-O-demethylnigerone, 8’-O-demethylnigerone, 8’-O-demethylisomigerone, dianhydroaurasperone C, 6,9-dibromoflavasperone, flavasperone, fonsecin, fonsecine B = fonsecin monomethyl ether, fonsecinone A-D, 2-hydroxydihydronigerone, isoaurasperone A,F, isonigerone, nigerasperone A-C, nigerone, rubasperone A-G, rubrofusarin, rubrofusarin B = heminigerone, rubrofusarin-6-O-α-D-ribofuranoside, (R)-10-(3-succimidyl)-TMC-256A1, TMC-256A1, B1, C1, C2)	Bouras et al. (2005, 2007); Lu et al. (2014); Choque et al. (2015); Happi et al. (2015); Li et al. (2013); Leutou et al. (2016); Li et al. (2016); Zhou et al. (2016)	Naphtho-γ-pyrones can be active against antibiotic resistant bacteria, have CNS repressant effects, inhibit Taq DNA polymerase, inhibit xanthine oxidase, inhibit acyl-CoA:cholesterol acyltransferase. Some produced only by *A. carbonarius* or *A. tubingensis*
Nigerasterol A & B	Liu et al. (2013)	Only produced by *A. tubingensis*
Nigerazine A & B	Iwamoto et al. (1983, 1985)	The producer strain was probably a *A. tubingensis*
Nigerloxin	Rao et al. (2002); Sing et al. (2016)	Identity of the producer strain is questionable
Nigragillin and aspernigerin	Caesar et al. (1969); Alvi et al. (2000); Shen et al. (2006); Frisvad et al. (2014); Bandara et al. (2015)	
Nygerone A and B	Henrikson et al. (2009)	
Penicillin/penicillin-like	Foster and Karow (1945)	Not yet confirmed
Pestalamide C	Bandara et al. (2015)	A tensidol
“Product B”	Lv et al. (2015)	Structure not known
Protocatechuic acid	Lv et al. (2014)	Small acid
Pseurotin A & D	Uchoa et al. (2017)	Identity of producer not convincingly confirmed
Pyranonigrin A-E, S	Hiort et al. (2004); Schlingmann et al. (2007); Miyake et al. (2008); Awakawa et al. (2013)	
Pyrophen	Barnes et al. (1990)	The producer strain was probably an *A. tubingensis*
Tensidol A and B	Fukuda et al. (2006); Henrikson et al. (2011)	
Tensyuic acid A-F	Hasegawa et al. (2007)	
Ustiloxin like cyclic ribosomal peptides	Nagano et al. (2016)	
Yanuthones (A-E, 22-deacetylyanuthone, 1-hydroxyyanuthone A-C)	Bugni et al. (2000); Holm et al. (2014); Petersen et al. (2015)	

Asperazine and similar diketopiperazine heterodimers (Varoglu et al. 1997; Li et al. 2015) are not produced by *A. niger*, but consistently by *A. tubingensis*, *A. vadensis*, and *A. luchuensis* (Nielsen et al. 2009; Varga et al. 2011a; Hong et al. 2013). However, such re-identifications from *A. niger* to *A. tubingensis* mean that co-occurrring metabolites are not necessarily produced by *A. niger*. For example, an asperazine- and asperazine A-producing isolate of *A. tubingensis* also produced cyclo(D-Phe-L-Trp), cyclo(L-Trp-L-Trp), walterolactone A, campyrones A-C, and kojic acid. According to our data, campyrones A-C are only produced by strains of *A. tubingensis*, and not by *A. niger* (but see Talontsi et al. 2013). Varoglu and Crews (2000) reported on asperic acid, hexylitaconic acid, malformin C, and pyrophen production by an asperazine-producing fungus, which should also be identified as *A. tubingensis*. Several of these compounds have later been found in *A. tubingensis* including 2-methylene-3-(6-hydroxyhexyl)-butanedioic acid, 2-carboxymethyl-3-hexyl-maleic acid anhydride, 2-methylene-3-hexyl-butanedioic acid (Almassi et al. 1994), demethylkotanin, TMC-256A1, TMC-256-C1 with an asperazine derivative (Ovenden et al. 2004), ergosterimide, 5,7-dihydroxy-2-[1-(4-methoxy-6-oxo-6H-pyran-2-yl)-2-phenylethylamino]-[1,4]naphthoquinone, asperamide A & B, aspergillusol, asperpyrone A & C, dianhydroauransperone C, fonsecinone A-D, isopyrophen, nigerasperone A–C, aurasperone A–B, pyrophen, cyclo(L-Trp-L-Ile), cyclo(L-Trp-L-Phe), cyclo(L-trp-L-Tyr) (Zhang et al. 2007a,b,c,d, 2010) asperic acid, campyrone A & C, tubigenoid anhydride A, 2-carboxymethyl-3-hexylmaleic anhydride (Koch et al. 2014), 6-isovaleryl-4-methoxy-pyran-2-one, asperpyrone A, campyrone A and rubrofusarin B (Ma et al. 2016), nigerapyrone A-E and asnipyrone A & B, and nigerasterols (Liu et al. 2011, 2013), and malformin A1, cyclo(Gly-L-Pro) and cyclo(Ala-Leu) (Tan et al. 2015). Gibberellic acid reported from *A*. “*niger*” NRRL 2270 (Ates and Gökdere 2006) is rather produced by *A. tubingensis* (this strain has indeed been reidentified as such) (Frisvad et al. 2011). A strain of *Pestalotiopsis theae* was probably overgrown by a strain of *A. tubingensis*, and thus, further secondary metabolites from *A. tubingensis* include pastalazine A & B and pestalamide A–C together with asperazine, aspernigrin A, and carbonarone A (see Ding et al. 2008).

A strain identified as *A. niger* was reported to produce asperiamide B and C (Wu et al. 2008), but it also produces the aflatoxin precursors averufin and nidurufin, so this strain was probably an *A. flavus*.

### Small acids of *Aspergillus niger*

Oxalic acid, gluconic acid, and citric acid are small chelating organic acids derived from the citric acid cycle, but since they are secreted and accumulated may be characterized as secondary metabolites (Poulsen et al. 2012; Niu et al. 2016). These are by far the small organic acids produced in the highest amounts, but other acids can be produced by *A. niger* (Table 5).

### Aflavinines

Aflavinines are indoloterpenes biosynthesized from tryptophan and dimethylallyl units. They are only produced in sclerotia of *A. niger* (Frisvad et al. 2014). Such sclerotia are not produced on ordinary laboratory media, except if they are induced by the presence of small dried fruits, such as raisins (Frisvad et al. 2014). Most aflavinines are antiinsectan, but are not known to be toxic towards vertebrates (Gloer et al. 1988).

### Asperamides

Asperamides are sphingolipids and unusual cerebrosides (Zhang et al. 2007a,b,c,d). Such sphingolipids appear to be pretty widespread in fungi, but their function in fungi is often unknown. The similar flavusides from *A. flavus* are antibacterial (Yang et al. 2011).

### Asperenones

The terpenes asperenones, asperyellones, and asperrubrols are carotenoid-like secondary metabolites. Asperenone is a human platelet aggregation inhibitor, and a strain of *A. niger* has been optimized for higher production of this bioactive compound (Chidananda et al. 2008).

### Aspergitides

Aspergitides are NRP-derived tetrapeptides which are potentially anti-inflammatory (Lee et al. 2015). These hydrophobic tetrapeptides have some similarity with fungisporins and nidulanins which appear to be generally present in *Aspergillus* and *Penicillium* species (Ali et al. 2014; Klitgaard et al. 2015; Hautbergue et al. 2017).

### Aspergillin

Aspergillin is a green polyketide (Ray and Eakin 1975) that may be connected with the production of the black pigment in the spores of *A. niger*. Other (yellow) pigments, such as funalenone and naphtho-γ-pyrones, are also connected with black melanin (Jørgensen et al. 2011).

### Aspernigrins

The aspernigrins, carbonarones, nygerones, pestalamides, pyrophen, and tensidols are all related 2-benzylpyridin-4-one-containing metabolites of non-ribosomal peptide (NRP) and polyketide origin. They have several effects such as inhibiting HIV virus, being antifungal, or having neuroprotective effects (Hiort et al. 2004; Ye et al. 2005; Ding et al. 2008, Bandara et al. 2015; Zhou et al. 2016). They have been isolated from *Aspergillus* section *Nigri* isolates and from fungi claimed to be *Cladosporium* (Ye et al. 2005) and *Pestalotiopsis theae* (Ding et al. 2008). The latter two fungi appear to have been overgrown by *Aspergillus niger* and *Aspergillus tubingensis*, respectively, as all secondary metabolites from these fungi have only been found in *Aspergillus* section *Nigri* (Nielsen et al. 2009).

### Azanigerones

The azanigerones A–F needed chromatin remodeling in order to be produced by *Aspergillus niger* (Zabala et al. 2012). These compounds are polyketides, and little is known of their activity. However, like other azaphilones, they can probably bind amino acids, but no nitrogen-containing derivatives have been found yet.

### Cycloleucomelone

Cycloleucomelone, leucomelone, and atromentin are shikimic acid-derived secondary metabolites that have been found in basidiomycetes (Turner 1971; Turner and Aldridge 1983) but also species in *Aspergillus* section *Nigri* (Hiort et al. 2004; Nielsen et al. 2009). These types of compounds may have radiation-protective characteristics, and they are widespread in *Aspergillus* (Frisvad and Larsen 2015). The analogous (heteroisoextrolites) terphenyllins are for example produced by members of *Aspergillus* section *Candidi* and aspulvinones by *Aspergillus* section *Terrei* (Turner 1971; Turner and Aldridge 1983; Frisvad and Larsen 2015).

### Funalenone and naphtho-γ-pyrones

These polyketides have some genes in common with the pksA gene for production of the black pigment in *Aspergillus niger* (Jørgensen et al. 2011). Some naphtho-γ-pyrones have been claimed to be toxic (Ghosal et al. 1979), but they are not mycotoxins according to the definition accepted here. In fact, they can be exploited industrially as they have anti-oxidant, anti-cancer, anti-microbial, anti-HIV, anti-hyperuricuric, and anti-tubercular effects (Choque et al. 2015).

### Malformins

Malformins are NRP cyclic peptides that originally were cited as toxic (Anderegg et al. 1976; Kobbe et al. 1977; Cole and Cox 1981), but they are not within the definition of mycotoxins in a strict sense, as malformin A has an oral LD_50_ of more than 50 mg/kg body weight in male mice. The toxicity data of Anderegg et al. (1976) and Kobbe et al. (1977) were based on malformin injection, which is not a natural route of intake. Furthermore, malformins have never been detected after mycotoxicosis caused by *A. niger*. Malformins are very promising anti-cancer agents, however (Park et al. 2017b).

### Nafuredin

Nafuredin is a polyketide terpene-derived secondary metabolite and is an inhibitor of anaerobic electron transport in pig roundworm, but it has very low effect on mammalian enzymes (Ui et al. 2001). It is a promising antihelminthic drug lead candidate.

### Nigerasterols

Nigerasterols are terpene-derived sterols that display potent activity against tumor cell lines (Liu et al. 2013). There are as yet no data on vertebrate toxicity. The fungus (MA-132) was identified only by using ITS sequences, so it may be another species in *Aspergillus* section *Nigri* than *A. niger* that produces nigerasterols.

### Nigerazines, aspernigerin, and nigragillins

The nigerazines, nigragillins, and aspernigerin are all related NRP-derived secondary metabolites. They are weakly insecticidal, and nigerazine B inhibits the root growth of lettuce seedlings (Caesar et al. 1969; Iwamoto et al. 1983). They have not been reported as mycotoxins.

### Nigerloxin

Nigerloxin is derived from an inhibitor of soy bean lipoxygenase and rat lens aldose reductase (Rao et al. 2002a,b). It is a polyketide NRP hybrid. It is a strong antioxidant and is anti-diabetic and of low toxicity (Rao et al. 2005; Suresha and Srinivasan 2013; Vasantha et al. 2018).

### Pseurotins

Pseurotins are NRP polyketide hybrid secondary metabolites that have neuritogenic (Komagata et al. 1996), antibiotic (Mehedi et al. 2010; Pinheiro et al. 2013), anti-inflammatory (Shi et al. 2015), chitin-synthase inhibitor (Wenke et al. 1993), and antileishmanial and anticancer (Martinez-Luis et al. 2012) characteristics. Pseurotin A & D was reported to be produced together with chlovalicin (Uchoa et al. 2017) probably coded by an intertwined gene clusters, as is the case for *Aspergillus fumigatus*, where pseurotin A and fumagillin, chemically closely related to chlovalicin, are coded by an intertwined gene cluster (Wiemann et al. 2013; Kishimoto et al. 2017). However, psurotins have not been reported from any other isolate of *A. niger* (Nielsen et al. 2009), so the two metabolites may be produced by another species in *Aspergillus* section *Nigri*.

### Pyranonigrins

The pyranonigrins A–K are NRP-PK derived antioxidant secondary metabolites from *A. niger* (Hiort et al. 2004; Miyake et al. 2007; Kishimoto et al. 2017). There are several pyranonigrins isolated from *Aspergillus niger*, including pyranonigrin A–K (Kishimoto et al. 2017).

### TAN-1612

The polyketide tetracyclic compound TAN-1612=BMS-192548 has been isolates from *Aspergillus tubingensis* WB 2346 and *A. niger* ATCC 1015 (Li et al. 2011). It is a neuropeptide Y receptor and neurokinin-1 receptor inhibitor (Kodukula et al. 1995; Shu et al. 1995).

### Tensyuic acids

The tensyuic acids are itaconic acid-derived secondary metabolites with anti-protozoan and antibacterial activities (Hasegawa et al. 2007; Matsumara et al. 2008).

### Yanuthones

The yanuthones are meroterpenoids with a 6-methyl salicylic acid precursor and terpene units attached (Holm et al. 2014; Petersen et al. 2015; Nielsen et al. 2017). There are no toxicity data for yanuthones, but they have antifungal activity (Petersen et al. 2015).

### Secondary metabolites described in *A. oryzae* and *A. flavus*

*A. flavus* and *A. oryzae* can produce many secondary metabolites (Table 7). These can be subdivided into biosynthetic families. It is very interesting to note that, e.g., ustiloxin B and ustilaginoidin C, have both been isolated from the rice false smut pathogen *Villosiclava virens* (= *Ustilaginoidea virens*) even though they are not biosynthetically related. However, these two types of secondary metabolites have also been found in *Aspergillus flavus* (Umemura et al. 2014; Tsukui et al. 2015; Yoshimi et al. 2016). This is remarkable as both unrelated fungi occur on rice. One speculation could be that the gene clusters for both ustiloxins and ustilaginoidins were horizontally transferred from one fungus to the other during evolution. Ustilaginoidins are bis-naphtho-γ-pyrones (even called “mycotoxins” in the paper of Meng et al. 2015 and ustiloxins for toxic cyclic peptides by Tsukui et al. 2015). Like the heteroisoextrolite (Frisvad and Larsen 2015) analogues in *Aspergillus* section *Nigri* (normally also called naphtho-γ-pyrones, Nielsen et al. 2009; Lu et al. 2014; Choque et al. 2015), the ustilaginoidins are probably also involved in the formation of the green conidium color of *Aspergillus* section *Flavi* as it is known for the involvement of naphtho-γ-pyrones in black pigmentation in *Aspergillus* section *Nigri* isolates (Chiang et al. 2011; Jørgensen et al. 2011; Frisvad et al. 2014; Niu et al. 2016). Other important secondary metabolites are described subsequently. Additionally, secondary metabolites that have been erroneously assigned to *A. flavus* or *A. oryzae* are also listed (Table 8).

**Table 7:** Primary and secondary metabolites reported from *Aspergillus flavus* and its domesticated form *A. oryzae* apart from aflatoxins and CPA-related compounds

Metabolite	Reference	Comment
Antioxidants: γ-tocopherol, d-tocopherol, genistin, daizein, genistein and 3-hydroxyanthranilic acid	Esaki et al. (1996); Matsuo (1997)	These are plant metabolites from *Glycine max* (soya) and not produced by the fungus, however the vitamins (tocopherols) could also be produced by *A. flavus* and *A. oryzae*
Asperfuran (= arthrographol)	Pfefferle et al. (1990); Ayer and Nozawa (1990)	
Aspergillic acid	White and Hill (1943); Dutcher (1947); Dunn et al. (1949); Hummel, 1956; Nakamura 1960; MacDonald (1961) ;Assante et al. (1981); Lebar et al. (2018); Saldan et al. (2018)	Aspergillic acids are strong iron chelators (Assante et al. 1981), and not produced by *A. oryzae*, but by *A. flavus*
Aspergillomarasmin and anhydromarasmic acid	Plattner and Clauson-Kaas (1945); Hardegger et al. (1963); Haenni et al. (1962; 1965); Robert et al. (1962); Lallouette (1962); Lederer (1962)	The related phytotoxin lycomarasmin is produced by *Fusarium* species
Asperopterin A & B	Matsuura et al. (1972)	Nucleobase derived
Aspirochlorin = oryzachlorin, dechloroaspirochlorine and O,O-dimethylaspirochlorine, trithioaspirochlorine	Kato et al. (1969); Berg et al. (1976); Sakata et al. (1982, 1983, 1987,b); Klausmeyer et al. (2005); Rank et al. (2012); Chankhamjon et al. (2014)	Original production strains classified as *A. tamarii*, *A. oryzae* and *A. flavus*
Biotin	Fukui et al. (1955a,b)	Vitamine
Bromoaspirochlorine	Sakata et al. (1987a,b)	Aspirochlorin biosynthetic family of compounds
Canadensolide	Sakata et al. (1982)	
Citric acid	Sakaguchi et al. (1953)	While *A. niger* can accumulate large amounts of citric acid, *A. flavus* only produce low amounts
Drim-9(11)-en-8-ol (R and S)	Wada et al. (1983); Leite et al. (1986); Domínguez et al. (1991); Shishido et al. (1991); Armstrong et al. (1996); Jansen and de Groot (1991; 2004)	
Flufuran, 5-(hydroxymethyl)-2-furancarboxylic acid, vanillic acid, 2-furanol, 2-(4.hydrophenyl)-ethanol, 3,4-dihydroxybenzoic acid	Evidente et al. (2009); Saldan et al. (2018)	
Fumaric acid	Sakaguchi et al. (1953)	Small acid
*l*-Glutamic acid	Kinoshita et al. (1961)	Amino acid
Heptelidic acid (= koningic acid), gliocladic acid, trichoderonic acid, hydroheptelidic acid	Lee et al. (2016); Skóra et al. (2017); Nishimura et al. (2018)	
Inositol	Fukui et al. (1955a,b)	Sugar alcohol
α-Ketoglutaric acid	Sakaguchi et al. (1953)	Small acid
Kojic acid, methyl kojic acid, dimethyl kojic acid	Saito (1907); Yabuta (1922); Tamiya (1927); Birkinshaw et al. (1931); Jennings and Williams (1945); Parrish et al. (1966); Morton et al. (1945); Marston (1949); Kistner (1962); Bentley (2006); Yang et al. (2011)	Production strains classified as *A. effusus*, *A. luteovirescens* or *A. lutescens*
Kojic acid-2 (BGY-F)	Zeringue et al. (1999)	Bright green flourescent molecule
Kojistatin A = CPI-4, CPI 1–3, CPI 5	Sato et al. (1996); Yamada et al. (1998)	
Lactic acid	Sakaguchi et al. (1953)	Small acid
*l*-Malic acid	Sakaguchi et al. (1953); Abe et al. (1961)	Small acid
Orange-red pigment	Manonmani and Sreektaniah (1984)	Unknown structure
Oryzacidin	Shimoda (1951)	C_8_H_13_O_5_N, an antibiotic
Oryzachlorin = Aspirochlorine = A 30641	Kato et al. (1969)	See Aspirochlorin
Pantothenic acid	Fukui et al. (1955a,b)	Vitamine
Penicillin	*A. oryzae*: Waksman and Bugie (1943); Foster and Karow (1945); Marui et al. (2010) *A. flavus*: White (1940); Bush and Goth (1943); McKee and MacPhillamy (1943); McKee et al. (1944); Waksman and Bugie (1943); Bush et al. (1945); Dey (1945); Adler and Wintersteiner (1948); Guida (1948)	
Pyrodoxine	Fukui et al. (1955a,b)	Vitamine
Riboflavin	Pontovich (1943); Zalesskaya et al. (1950); Mogi et al. (1952); Higuchi (1956)	Vitamine
Sporogene AO1	Tanaka et al. (1984a,b); Tamogami et al. (1996)	
Succinic acid	Srinisavan and Ramakrishnan (1952); Sakaguchi et al. (1953)	Small acid
Thiamine	Fukui et al. (1955a,b)	Vitamine
Ustilaginoidin C	Brown et al. (2003)	Conidium pigment
Violacetin	Kobayashi (1966)	Probably a mistake, most likely originated from a contaminating Actinomycete
Vitamine B_12_ (cyanocobalamine) and K3	Sakai (1953); Ramakrishnan and Sathe (1956)	Vitamine, production strain *A. oryzae* var. *microsporus*
Ustiloxin B	Umemura et al. (2013); Nagano et al. (2016); Ye et al. (2016); Yoshimi et al. (2016)	No production in *A. oryzae* RIB40

**Table 8:** Secondary metabolites *erroneously* ascribed to *Aspergillus flavus* or *A. oryzae*

Metabolite	Reference	Comment
Aflatoxins in *A. oryzae*	El-Hag and Morse (1976) (see Fennell, 1976); El-Kady et al. (1994); Atalla et al. (2003)	Aflatoxin production reported from NRRL 1988 was refuted by Fennell (1976). The culture was a mixed culture with a strain of *Aspergillus parasiticus*. Later reports on aflatoxin production by *A. oryzae* were erroneous (Varga et al. 2009)
Aflatoxin G1 in *A. flavus*	Saldan et al. (2018)	Aflatoxin G_1_ has only been found very rarely in *A. flavus* but has been found more often in other species in section *Flavi* (Frisvad et al. 2019)
Asperaculin A	Son et al. (2018)	Identity of strain (KCCM 12698) and compound dubious, compound only tentatively assigned
Asperentin = cladosporin, asperentin 8-O-methylether, asperentin 6-O-methyl ether, 5′-hydroasperentin	Grove (1972a, 1973a)	Producer strain is *A. pseudoglaucus* (Chen et al. 2017)
Asperflavin, anhydroasperflasvin, 5,7-dihydroxy-4-methylphthalide	Grove (1972b)	Producer strain is *A. pseudoglaucus* (Chen et al. 2017)
Asporyzin A, B and C	Qiao et al. (2010a); Nozawa et al. (1988); Kimura et al. (1992)	Producer strain is *A. niveus*, *A. cejpii* or *A. striatus*
Austalide F & H	Son et al. (2018)	Identity of strain and compound dubious, compounds tentatively assigned
Aspyrone	Saldan et al. (2018)	Identity of strain and compound dubious
Betaine	Saldan et al. (2018)	Identity of strain and compound dubious
Chrysogine	Saldan et al. (2018)	Chrysogine has not been found in *A. flavus*, but in other members of *Aspergillus* section *Flavi* (Frisvad et al. 2019)
Cyclopenol	Zhuravleva et al. (2016)	Producer strain was probably *Aspergillus amoenus*
Deacetoxyscirpenol (DON)	Rahssaparpoor (2014)	Misidentification of compound. Producer strain was claimed to be *A. flavus*
Deacetylparasiticolide A	Saldan et al. (2018)	Identity of strain and compound dubious, parasiticolides have not been found in *A. flavus*, but in other members of *Aspergillus* section *Flavi* (Frisvad et al. 2018)
Decumbenone B	Zhuravleva et al. (2016)	Producer strain is probably *Aspergillus amoenus*
5,7-Dihydroxy-4-methylisobenzofuran-1-(3H)-one	Grove (1972a,b); Kobayashi et al. (1990)	*A. pseudoglaucus* is the actual producer of this compound
Dihydroxymethoxycoumarin & ketone-citreoisocoumarin	Son et al. (2018)	Identity of strain and compound dubious; compound tentatively assigned
Emindole SB	Qiao et al. (2010a); Nozawa et al. (1988); Kimura et al. (1992)	Producer strain is *A. niveus*, *A. cejpii* or *A. striatus*
Emeniveol	Qiao et al. (2010a); Nozawa et al. (1988); Kimura et al. (1992)	Producer strain is *A. niveus*, *A. cejpii* or *A. striatus*
Gliotoxin	Lewis et al. (2005); Kupfahl et al. (2008)	Data not substantiated (Patron et al. (2007); Manzanares-Miralles (2016); Vidal-Garcia et al. (2018)
Gregatin B	Saldan et al. (2018)	Identity of strain and compound dubious
Hexylitaconic acid	Son et al. (2018)	Identity of strain and compound dubious; compound tentatively assigned by MS
4-hydroxy-asperentin, 5′-hydroxyasperentin 8-methyl ether	Grove (1973b)	*A. pseudoglaucus* is the actual producer of this compound
Hydroxysydonic acid	Saldan et al. (2018)	Identity of strain and compound dubious
Isoflavipucine	Mituzani et al. (2016)	Producer strain is *A. flavipes*, not *A. flavus*
JBIR-03	Qiao et al. (2010a); Nozawa et al. (1988); Kimura et al. (1992)	Producer strain is *A. niveus*, *A. cejpii* or *A. striatus*
Kipukacin J	Zhuravleva et al. (2016)	Producer strain is *Aspergillus amoenus*
Maltoryzin	Iizuka and Iida (1962); Bakhali et al. (2013)	Assigned to *A. flavus* var. *microsporis* but the producer strain is probably *A. clavatus* (Varga et al. 2007)
Mycophenolic acid	Kobayashi et al. (1990)	*A. pseudoglaucus* is the actual producer of this compound (Chen et al. 2017)
Nicotinic acid	Saldan et al. (2018)	Identity of strain and compound dubious
Nivalenol, deoxynivalenol, T-2 toxin	Elsahrkawy and Abbas, 1991; Atalla et al. (2003)	Apparently both fungus and mycotoxin were misidentified in this work, the substrate was contaminated, or the trichotecenes were biotransformed (also the case for *A. niger*)
Ochratoxin A and B	Atalla et al. (2003)	Apparently both fungus and mycotoxin were misidentified in this work
Pentahydroxy-anthraquinone	Son et al. (2018)	Identity of strain and compound dubious; compound tentatively assigned
Omoflavipucine	Mituzani et al. (2016)	Producer strain is *A. flavipes*, not *A. flavus*
Phomaligin A	Saldan et al. (2018)	Identity of strain and compound dubious
Spinulosin	Saldan et al. (2018)	Identity of strain and compound dubious
Sterigmatocystin in *A. oryzae*	Atalla et al. (2003)	Apparently both fungus and mycotoxin were misidentified in this work
(3S,6S)-Terramide A and B	Garson et al. (1986)	Listed as being produced also by *A. flavus*, in addition to *A. terreus* in AntiBase, no references could be found to the possible fact that *A. flavus* can produce terramides
Terrein	Saldan et al. (2018)	Identity of strain and compound dubious
Taxol	El-Sayed et al. (2018)	Both producer and secondary metabolite production needs to be verified
Violacetin	Kobayashi (1966); Aiso et al. (1955)	Violacetin is a *Streptomyces* secondary metabolite, not of fungal origin
Zearalenone	Atalla et al. (2003)	Apparently both fungus and mycotoxin were misidentified in this work

### Aflatrems

Aflatrem and β-aflatrem and their precursors are indoloterpenes that have been found in sclerotia of *Aspergillus flavus* (Gallagher and Wilson 1980; Gallagher et al. 1980a,b; Valdes et al. 1985; Tanaka et al. 1989; TePaske et al. 1992; Zhang et al. 2004; Duran et al. 2007; Nicholson et al. 2009; Ehrlich and Mack 2014; Tang et al. 2015; Gilbert et al. 2016). *Aspergillus oryzae* RIB 40 was found to produce the 13-desoxypaxilline precursor to aflatrem (Rank et al. 2012), and aflatrem has been heterologously expressed in *A. oryzae* NSAR1 (Tagami et al. 2014). However, if RIB40 is indeed a real *A. flavus*, *A. oryzae* sensu stricto isolates are not be able to produce sclerotia and sclerotial metabolites such as aflatrem.

### Aflavarins

Aflavarins are polyketides found in the sclerotia of *Aspergillus flavus* (TePaske et al. 1992). These polyketides have not yet been found in any *A. oryzae* strain. Leporins, also found in *A. leporis* (TePaske et al. 1991), have been found in *A. flavus* (Cary et al. 2015), but they are not expected to be produced by *A. oryzae*.

### Aflavinins

The aflavinins are sclerotium-borne indoloterpenes first isolated from *A. flavus* (Gallagher et al. 1980a,b; Cole et al. 1981; Wicklow and Cole 1982; Gloer et al. 1988). These indoloterpenes and aflavazol were also isolated from the sclerotia of *A. oryzae* RIB40 (TePaske et al. 1990; Rank et al. 2012). The aflavinins isolated from *A. flavus* (possibly *A. minisclerotigenes* or *A. aflatoxiformans*) include aflavinine, dihydroxyaflavinine, monohydroxyaflavinine, and monohydroxyisoaflavinine (Nozawa et al. 1989; Tang et al. 2015).

### Asperfuran

Asperfuran is a dihydrobenzofuran compound that was isolated from *Aspergillus oryzae* “HA 302-84” (Pfefferle et al. 1990), but it has also been isolated under the name of arthrographol from *Arthrographis pinicola* (Ayer and Nozawa 1990) and as asperfuran from *Penicillium* species (Yamaji et al. 1999; Frisvad et al. 2004, 2006). Asperfuran is antifungal, but there are no reports on toxicity of this compound. Asperfuran production by authentic strains of *A. oryzae* has later been confirmed, and it has also been detected in *Aspergillus sojae* (Varga et al. 2011b).

### Aspergillic acids

These iron-chelating compounds have been used for discrimination between *A. flavus* and *A. oryzae*, in that *Aspergillus oryzae* sensu stricto has been claimed not to produce any of these pyrazine compounds. Testing *Aspergillus flavus* sensu stricto and *Aspergillus oryzae* sensu stricto has shown that it is only the former that can produce aspergillic acids (Bothast and Fennell 1974; Hamsa and Ayres 1977; Pitt et al. 1983; Assante et al. 1981; Liljegren et al. 1988; Varga et al. 2011b). However, compounds in this class have been reported from *A. oryzae*, including mutaaspergillic acid (Nakamura and Shiro 1959a,b; Nakamura 1961; Sugiyama et al. 1967; Ohta and Ohta 1983), hydroxyaspergillic acid (Nakamura and Shiro 1959a,b; Dutcher 1958 (as *A. flavus*); MacDonald 1962; Ohta and Ohta 1983; Sano et al. 2007), VI-2 (Ueno et al. 1977), A-2 (Sano et al. 2007), and aspergillic acid (Nishimura et al. 1991). Aspergillic acids have been evaluated for toxicity (Sasaki et al. 1968; MacDonald 1973; Perry et al. 1984), but Sano et al. (2007) suggest that the toxicity of aspergillic acids is so low that it can be present in fermented foods used for consumption. The strains producing aspergillic acid, indicated by the medium AFPA (*Aspergillus flavus parasiticus* agar), are probably representing *Aspergillus flavus* sensu stricto, but because of issues with potential aflatoxin production, they are called *A. oryzae* “short stipes.” The indicative red-orange color is caused by reaction of ferric ions with aspergillic acids (Assante et al. 1981) with none of these strains have been reported to produce aflatoxins (Sano et al. 2007).

### Aspergillomarasmins

Aspergillomarasmin A, anhydroaspergillomarasmin A, and anhydromarasmic acid are polyamino acid compounds/phytotoxins related to lycomarasmin from *Fusarium* (Plattner and Clauson-Kaas 1945; Hardegger et al. 1963), but they have also been found in *Aspergillus oryzae* or *A. flavus* (*A.* “*flavus oryzae*”) (Haenni et al. 1962; Haenni et al. 1965; Robert et al. 1962). Aspergillomarasmin A is very interesting as it inhibits metallo-beta-lactamases and could thus help in overcoming bacterial resistance to penicillin (King et al. 2014; Koteva et al. 2016). There are no data of toxicity of these compounds yet.

### Asperopterins

Asperopterin A and B are compounds containing a pteridin ring system that were isolated from *Aspergillus oryzae* “T-17” (Kaneko and Sanada 1969; Matsuura et al. 1972; Hanaka et al. 2012) and have since been synthesized (Sugimoto et al. 1986; Hanaka and Yamamoto 2013). Unfortunately, the original producer strain is not available, and there are no toxicity data available for the asperopterins. These compounds are blue fluorescing, so if they are produced by *A. oryzae* sensu stricto, these may be the compounds that could have been erroneously detected and identified as aflatoxins.

### Aspirochlorines

Aspirochlorine is a halogenated diketopiperazine with a central disulfide bridge that was first isolated from *Aspergillus oryzae* IAM-2613 under the name oryzachlorin (Kato et al. 1969). The compound has also been chemically synthesized (Miknis and Williams 1993; Wu et al. 2000). However, oryzachlorin was later shown to be the same as aspirochlorine and A30641 (Sakata et al. 1982, 1983, 1987a, 1987b). It was isolated under the name A30641 from *Aspergillus tamarii* NRRL 8101, where it was co-occurring with canadensolide (Berg et al. 1976), as was also the case of a strain identified as *A. flavus* (Sakata et al. 1982). A strain of the latter was not available for more detailed studies. Another strain identified as *A. flavus* (“MDH-1420”) was shown to produce aspirochlorin and the related compound tetrathioaspirochlorine, and evidence for presence of the trithio analogue also (Klausmeyer et al. 2005). Furthermore, a bromoaspirochlorin, dechloroaspirochlorine, and O,O-dimethylaspirochlorine have been reported (Sakata et al. 1987). Aspirochlorin has been shown to be a highly selective and potent inhibitor of protein synthesis (Monti et al. 1999) and an effective inhibitor of fungi, bacteria, viruses, and murine tumor cells (Monti et al. 1999; Chankhamjon et al. 2014). For these reasons and because epipolythiodiketopiperazines are generally toxic, the latter authors called aspirochlorin for a mycotoxin. The related mycotoxin gliotoxin was reported from 4 and 13% of clinical *Aspergillus flavus* strains (Lewis et al. 2005; Kupfahl et al. 2008), but there is some doubt whether these data are correct (Patron et al. 2007; Manzanares-Miralles et al. 2016; Vidal-Garcia et al. 2018). On the other hand, Shaaban et al. (2014) isolated the reduced form of gliotoxin from *A*. “*oryzae*” MMAO1, and this latter isolate could be *Aspergillus flavus* sensu stricto. Gliotoxin-producing isolates have not been available for the scientific community (Varga et al. 2011b). Aspirochlorin is a product of many species in section *Flavi*: *A. avenaceus*, *A. caelatus*, *A. oryzae*, *A. parvisclerotigenus*, *A. sojae*, and *A. tamarii* (Varga et al. 2011b).

### Asporyergosterol

Asporyergosterol and several other sterols were isolated from isolated from *Aspergillus oryzae* “cf-2” = CCTCC M 2010045, isolated from a marine alga (Qiao et al. 2010b). The strain isolated could equally well be another *Aspergillus*, as *A. oryzae* in principle cannot be isolated from natural sources. An oxylipin and several sterols were isolated from *A. flavus*, isolated from an alga by the same authors (Qiao et al. 2011). The isolate also produced emeniveol and similar compounds and could probably in reality be *A. cejpii*, *A. niveus*, or *A. striatus*.

### Avenaciolides and canadensolides

Canadensolides are formed via condensation of an acetate derived chain with a TCA cycle intermediate (Brookes et al. 1963; Turner 1971; Tanabe et al. 1973). It was first isolated from “*Penicillium*” *canadense* (McCorkindale et al. 1968), but it has been reported once from *A. flavus* (Sakata et al. 1982), and the related avenaciolide has been reported from *A. avenaceus* in *Aspergillus* section *Flavi* (Brookes et al. 1963; Tanabem et al. 1973; Varga et al. 2011b). The avenaciolides are also present in *Aspergillus glaber* and *A. stramenius* from *Aspergillus* section *Fumigati* (Ellis et al. 1964; Samson et al. 2007), and the avenaciolides are active against methicillin-resistant Staphylococci (Chang et al. 2015). Avenaciolide is also a specific inhibitor of glutamate transport in rat liver mitochondria (McGivan and Chappell 1970). Isoavenaciolide has been reported as an anti-cancer agent (Al-Tel et al. 2009). However, there are no indications that avenaciolide is a mycotoxin.

### Csypyrones

Type III polyketides are rare among fungi, but more common in plants and bacteria (Juvvadi et al. 2005; Hashimoto et al. 2014; Shimizu et al. 2017). *Aspergillus oryzae* can, however, produce csypyrone B1, B2, and B3 and 3,5-dihydroxybenzoic acid (Seshime et al. 2005, 2010a,b; Hashimoto et al. 2013). Interestingly, *Aspergillus niger* produces protocatechuic acid, also a type III polyketide (Lv et al. 2014). Other fungi that can produce type III polyketides are and *Botrytis cinerea* (Hashimoto et al. 2014). There are no toxicity data for these secondary metabolites.

### Drim-9(11)-en-8-ol (R and S)

This sesquiterpene compound has been isolated from *A. oryzae* strains that also produce sporogen AO1 and similar compounds, but very little is known on the bioactivity of this compound (Wada et al. 1983; Leite et al. 1986; Domíngues et al. 1991; Shishido et al. 1991; Armstrong et al. 1996; Jansen and de Groot 1990; 2004).

### Flufuran

Flufuran, other related furans, and small molecular weight secondary metabolites, including 4-hydroxybenzoic acid, were isolated from *Aspergillus oryzae* and *A. flavus* (Evidente et al. 2009; Lee et al. 2016; Saldan et al. 2018). Flufuran has antifungal activity (Evidente et al. 2009).

### Heptelidic acids

Heptelidic acid (=koningic acid), hydroheptelidic acid, gliocladic acid, and trichoderonic acid are sesquiterpenes that have antibiotic and anticancer properties (Itoh et al. 1989; Nakazawa et al. 1997; Kim and Lee 2009). Heptelidic acid has been reported from both *Aspergillus oryzae* and *A. flavus* (Lee et al. 2016; Skóra et al. 2017).

### Kojic acids

Kojic acid was the first compound to be isolated from *Aspergillus oryzae* (Yabuta 1912, 1922; Birkinshaw et al. 1931; Jennings and Williams 1945; Parrish et al. 1966; Morton et al. 1945; Marston 1949; Bentley 2006). A dimer of kojic acid has been structure-elucidated as the bright greenish yellow flourescence pigment from *Aspergillus flavus* (Zeringue et al. 1999). Koji acid is common for nearly all species in *Aspergillus* section *Flavi* (Varga et al. 2011b). However, kojic acid is not regarded as a mycotoxin (Bentley 2006). The gene cluster coding for kojic acid production is known (Terabayashi et al. 2010). 7-O-acetylkojic acid has also been isolated from *A. flavus* (Sun et al. 2014).

### Kojistatins

An isolate of an industrial strain of *Aspergillus oryzae* (ATCC 20386 and FERM-15834) produced kojistatin A = CPI-4 and related cystein protease inhibitors, called CPI 1-5 (Sato et al. 1996; Yamada et al. 1998). The kojistatins are nonribosomal peptide–polyketide hybride molecules. There are no data on the toxicity of these compounds.

### Maltoryzin

The polyketide maltoryzin was reported from a strain of *A. oryzae* var. “*microsporis*” isolated from malting barley (Iizuka and Iida 1962). However, the fungus could also be an *Aspergillus clavatus*, which is very common in malting barley (Lopez Diaz and Flannigan 1997). *A. oryzae* or *A. flavus* has not been reported from malting barley. On the other hand, Bakhali et al. (2015) reported on maltoryzin production by *A. flavus* from walnuts.

### Miyakamides

Miyakamides A_1_, A_2_, B_1_, and B_2_ (Shiomi et al. 2002), and oryzamide A_1–2_ (Rank et al. 2012) have been reported from both *Aspergillus flavus* “var. *columnaris*” FKI-0739 and *A. oryzae* RIB40 and are NRPs. The miyakamides are antimicrobial compounds, but there are no toxicity data on these compounds. Since the *A. flavus* strain FKI-0739 produced hydroxyaspergillic acid also (Shiomi et al. 2002), it was probably an *A. flavus* sensu stricto. As discussed earlier, RIB40 may also in reality be an *A. flavus* sensu stricto.

### Oryzaeins

The polyketides oryzaein A–D, tabaisocoumarin A, caudacoumarin C, versicolol B, and exserolide D and F are antiviral and cytotoxic isocoumarin derivatives isolated from a fungus identified as *A. oryzae* isolated from the rhizome of the marine *Paris polyphylla* var. *yunnanensis* (Zhou et al. 2016), and thus, the producing strain is probably an *A. flavus* sensu stricto. However, compounds with isochroman chromophores have been found in extracts of some *A. oryzae* (Frisvad JC, “personal data”). The four oryzaeins had moderate to weak inhibitory effect against some human tumor cell lines (Zhou et al. 2016), but their actual toxicity is unknown.

### Oryzines

Oryzines are maleidrides biosynthetically produced from acyl CoA thiolester and from oxaloacetic acid (Wasil et al. 2018). RIB 203, the producing strain, is from sake-koji and thus represents a real *A. oryzae*. There are no data on the bioactivity of these compounds as yet.

### Parasiticolides

Parasiticolide A = astellolide A is a sesquiterpene that was first found in *Aspergillus parasiticus* from section *Flavi* (Hamasaki et al. 1975) and *Aspergillus stellatus* from section *Nidulantes* (Gould et al. 1981), and later parasiticolide A, dideacetylparasiticolide A, and 14-deacetyl parasiticolide A were isolated from *A. oryzae* RIB40 (Rank et al. 2012). Ren et al. (2015) found astellolides A, B, C–E, and F–I in *Aspergillus oryzae* QXPV-4 isolated from the insect *Coccinella septempunctata*. The origin of QXPV-4 indicates that this was also an *A. flavus* sensu stricto, rather than an *A. flavus*. Shinohara et al. (2016) also found parasiticolides = astellolides in *A.oryzae* RIB40: 14-deacetyl astellolide A = 14-deacetyl parasiticolide A (already found by Rank et al. 2012), and 14-deacetyl astellolide B. Depending on the opinion of the taxonomic status of *A. flavus* and *A. oryzae*, parasiticolides are secondary metabolites of one of these species or both.

### Penicillins

Penicillins are non-ribosomally synthesized tripeptides (NRP) that have been reported from *A. oryzae* (Waksman and Bugie 1943; Foster and Karow 1945; Marui et al. 2010) and *A. flavus* originally as flavicidin (Bush and Goth 1943; McKee and MacPhillamy 1943; McKee et al. 1944; Waksman and Bugie 1943; RG Benedict, unpublished in Raper 1946). This important antibiotic is not regarded as a mycotoxin, but it is unwanted in industrial fermentations due to its wide use to treat microbial infections.

### Pseurotins

Pseurotins are hybrid NRP/PKS compounds that have been found in *A. leporis* and *A. nomius* from *Aspergillus* section *Flavi* (Varga et al. 2011b) and were also reported from *A*. “*oryzae*” MMAO1 (Shaaban et al. 2014) and *A. flavus* (Rodríguez et al. 2015). The pseurotins are not regarded as mycotoxins, but these compounds should be examined in more detail, as they have neuritogenic (Komagata et al. 1996), antibiotic (Mehedi et al. 2010; Pinheiro et al. 2013), anti-inflammatory (Shi et al. 2015), chitin-synthase inhibitor (Wenke et al. 1993), and antileishmanial and anticancer (Martinez-Luis et al. 2012) characteristics.

### Sporogens

Sporogen AO1 (=13-desoxyphomenone) is a sesquiterpenoid that was isolated from *Aspergillus oryzae* NOY-2, but the strain is not available to the scientific community. This compound induces conidiation in a less sporulating strain (Tanaka et al. 1984,b). Sporogen AO1 has later been found in strains of *A. flavus* (Frisvad and Larsen, unpublished). Phomenone, related to sporogen AO1, is a potent inhibitor of protein synthesis (Moule et al. 1977) and has moderate toxicity to shrimps (Capasso et al. 1984). Phomenone was recently shown to stimulate pro-inflammatory responses in murine cells and thus may exacerbate allergic reactions if inhaled (Rand et al. 2017). There are no direct data showing that these compounds are mycotoxins, but they are not unlike the mycotoxin PR-toxin in structure (Cole and Cox 1981; Moule et al. 1977; Capasso et al. 1984). Many of the sporogens are phytotoxins (Daengrot et al. 2015).

### TMC-2A, -2B and -2C

The NRP-derived peptide-like compounds TMC-2A, -2B, and 2C were isolated from a strain identified as *A. oryzae* A374 = FERM P-14934 (Nonaka et al. 1997; Asai et al. 1997). From the description of the strain, and as it was isolated from soil, it appears that the strain is rather an *Aspergillus tamarii*, as the conidia were large, distinctly roughened, and brown. These peptide-like compounds may be used as lead compounds to find better rheumatoid arthritis inhibitors, but toxicity data have not been presented.

### Tryptophenalins

A fungus identified as *A. oryzae* (MMAO1) was isolated from rice hulls, and this fungus produced a dimeric diketopiperazine compound, ditryptophenaline, 7,9-dihydroxy-3-(1H-indol-3-ylmethyl)-8-methoxy-2,3,11,11a-tetrahydro-6H-pyrazino[1,2-b]isoquinoline-1,4-dione, cyclo-(Trp-Tyr), cyclo-(Pro-Val), α-cyclopiazonic acid, (bismethylthio)gliotoxin, pseurotin A, kojic acid, linoleic acid, and uridine (Shaaban et al. 2014). Since the isolate was from rice hulls in a domesticated field, it was probably an *A. flavus*, but it could also be an *A. nomius* since pseurotin A has only been found once in *A. flavus* (see Varga et al. 2011b; Rodríguez et al. 2015). Ditryptoleucine, related to ditryptophenaline from *A. flavus* (Springer et al. 1977) was isolated from *A. oryzae* RIB40 (Rank et al. 2012). The toxicity of the diketopiperazines cyclo-(Trp-Tyr), cyclo-(Pro-Val) is unknown. The monomer of ditryptophenaline, cyclo-N-methylphenyl-alanyltryptophanyl has also been isolated (Kozlovskii et al. 1990).

### Ustilaginoidins

The polyketide ustilaginoidin C was isolated as a suggested conidium pigment from *A. parasiticus* (Brown et al. 2003), and a compound with the same chromophore has been isolated from *A. flavus* (Frisvad, JC, unpublished data), so it could be representing the general naphtho-γ-pyrone pigment type produced in *Aspergillus* section *Flavi*. There are no toxicity data on these compounds.

### Ustiloxin B

This ribosomally produced cyclic peptide (RIPS, ribosomally produced peptides) compound was isolated from *Aspergillus flavus* and *A. oryzae* (Umemura et al. 2013, b, 2014; Ye et al. 2016; Yoshimi et al. 2016). The ustiloxins are phytotoxins first isolated from *Villosiclava virens* (= *Ustilaginoidea virens*), and they exibit potent antimitotic activity and inhibit microtubule assembly (Koiso et al. 1994), and they have also been called mycotoxins (Koiso et al. 1992). The ustiloxins are not established as mycotoxins. *A. oryzae* RIB40 does not produce ustiloxin B, probably because of the large deletion of the ustR gene encoding a transcriptional regulation that regulates ustiloxin B production (Umemura et al. 2014).

### Secondary metabolites that are not produced by *A. flavus* or *A. oryzae*

Due to the close relatedness between *A. flavus* and *A. oryzae* as well as their similarity to other species, some reports have misleadingly described production of secondary metabolites in *A. flavus* or *A. oryzae* that they do not produce (Table 8).

### Secondary metabolites described in *T. reesei*

Peptaibol non-ribosomal peptides (peptaibiotics) and similar peptides are produced by many *Trichoderma* species (Zeilinger et al. 2016), but it is only paracelsin A, C, and D in this class that have been reported from *T. reesei* (Brückner and Graf 1983; Brückner et al. 1984; Pócsfalvi et al. 1997; Przybylski et al. 1984). The paracelsins were reported from an authentic strain of *T. reesei* (QM 9414 (mutant of QM 6a) = ATCC 26421 = CBS 392.92 and the wild ex type strain from cotton duck shelter, Bougainville Island QM 6a (= ATCC 13631 = CBS 383.78). Paracelsins are linear peptides containing a high level of uncommon amino acids, alphaaminoisobutyric acid (Aib), and isovaline (Iva), together with an acetylated N-terminal amino acid and a C-terminal amino alcohol (Pócsfalvi et al. 1997). These compounds have shown antimicrobial activity. There are no data on the toxicity of the paracelsins.

The sorbicillin biosynthetic family compounds have been reported from *Trichoderma* sp. USF 2690 (Abe et al. 2001) (strain not available in any culture collection), and it is only mentioned to be a product of *T. reesei* in the Antibase secondary metabolite database. The Trichodermatides (A–D) are produced by a fungus claimed to be a marine *T. reesei* (Sun et al. 2008; Shigehisa et al. 2015), but the culture is unavailable in culture collections, and may be one of the many other known *Trichoderma* species. *T. reesei* may also produce some other non-ribosomal peptides, including intracellular and extracellular siderophores (Zeilinger et al. 2016). Siderophores such as ferricrocin have not been claimed to be toxic. Among the polyketides, the genes for a conidium pigment related to aurofusarin and bikaverin have been reported (Zeilinger et al. 2016). This polyketide compound (not structure elucidated) is probably a precursor for the green pigment (melanin) in the conidia of *T. reesei*, and generally, these conidium pigments have not been claimed to be toxic. *T. reesei* have PKS gene clusters for production of other polyketides, which are not unlike those for citrinin and fumonisins (Baker et al. 2012), but neither citrinin nor fumonisins have been detected in *T. reesei*. In conclusion, the only secondary metabolites that appear to be naturally produced by *T. reesei* are the paracelsins.

Based on genome sequencing data (Schmoll et al. 2016), several potential toxic secondary metabolites may be produced under special conditions. Such secondary metabolites have not been detected yet in *T. reesei*, however. Genome sequencing showed that there are 8 NRKS, 11 PKS, 2 NRPS-PKS hybrid, and 12 terpenoid synthase encoding genes (Schmoll et al. 2016; Zeilinger et al. 2016). The *LaeA* and VELVET regulatory genes are important for the expression of secondary metabolites in *T. reesei*, but nevertheless, only few of the putative gene clusters for secondary metabolites seem to be actually expressed.

## Conclusions

*Aspergillus oryzae* produce few recognized mycotoxins, and they are only produced by few strains. If they are produced, there are genetic means of inactivating the biosynthetic pathways, so isolates of the species can be exploited for production of enzymes and as a transformation host for industrially relevant secondary metabolites or enzymes. Some isolates of *Aspergillus niger* can produce three types of mycotoxins, ochratoxin A, fumonisin B_2_ (B_4_ and B_6_), and oxalic acid. Again, genetic means have been employed to inactivate the gene clusters for ochratoxins and fumonisins, while accumulation of the less toxic oxalic acid can be avoided by chosing an optimal substrate or use optimal procedures for the industrial products. *Trichoderma reesei* cannot produce any recognized mycotoxins and is one of the most important enzyme producers in the industry. All three species can produce interesting secondary metabolites, of which some are drug lead candidates and others, such as citric acid, are important bulk chemicals that are produced by fermentation.

## References

[CR1] Abarca ML, Bragulat MR, Castella G, Cabanes FJ (1994). Ochratoxin A production by strains of *Aspergillus niger* var. *niger*. Appl Environ Microbiol.

[CR2] Abdelghany TM, El-Nagger MA, Ganash MA, Al Abboud MA (2017). PCR identification of *Aspergillus niger* with using natural additives for controlling and detection of malformins and maltoryzine production by HPLC. BioNanoSci.

[CR3] Abe K, Gomi K, Hasegawa F, Machida M (2006). Impact of *Aspergillus oryzae* genomics on industrial production of metabolites. Mycopathologia.

[CR4] Abe S, Saito T, Takayama KI, Furuya A (1961) L-Malic acid by fermentation. British patent no. 884,029, December 6

[CR5] Abe N, Sugimoto O, Arakawa T, Tanji K, Hirota A (2001). Sorbicillinol, a key intermediate of bisorbicillinoid biosynthesis in *Trichoderma* sp. USF-2690. Biosci Biotechnol Biochem.

[CR6] Adler M, Wintersteiner O (1948). A reinvestigation of flavacidin, the penicillin produced by *Aspergillus flavus*. J Biol Chem.

[CR7] Aiso K, Arai T, Shidara I, Kurihara H, Morita Y (1955). A new broad spectrum antibiotic, violacetin. J Antibiot.

[CR8] Ali H, Ries MI, Lankhorst PP, van der Hoeven RAM, Schouten OL, Noga M, Hankemeier T, van Peij RAL, Vreeken RJ, Driessen AJM (2014). A non-canonical NRPS is involved in the synthesis of fungisporin and related hydrophobic cyclic tetrapeptides in *Penicillium chrysogenum*. PLoS ONE.

[CR9] Almassi F, Ghisalberti EL, Rowland CY (1994). Alkylcitrate-derived metabolites from *Aspergillus niger*. J Nat Prod.

[CR10] Al-Musallam A (1980). Revision of the black *Aspergillus* species.

[CR11] Al-Tel TH, Al-Qawasmeyh R, Sabri SS, Voelter W (2009). Differential use of anhydropyranosides for eantiomere routes to bis-g-butyrolactones: a new approach to the frameworks of antibiotic and anticancer agents isoavenaciolide and ethisolide. J Org Chem.

[CR12] Anderegg Robert J., Biemann Klaus, Buechi George, Cushman Mark (1976). Malformin C, a new metabolite of Aspergillus niger. Journal of the American Chemical Society.

[CR13] Andersen MR, Salazar MP, Schaap PJ, van de Vondervoort PJI, Culley D, Thykaer J, Frisvad JC, Nielsen KF, Albang R, Albermann K, Berka RM, Braus GH, Braus-Stromeyer SA, Corrochano LM, Dai Z, van Dijck PWM, Hofmann G, Lasure LL, Magnusson JK, Meijer SL, Nielsen JB, Nielsen ML, van Ooyen AJJ, Panther KS, Pel HJ, Poulsen L, Samson RA, Stam H, Tsang A, van den Brink JM, Atkins A, Aerts A, Shapiro H, Pangilinan J, Salamov A, Lou Y, Lindquist E, Lucas S, Grimwood J, Grigoriev IV, Kubicek CP, Martinez D, van Peij NNME, Roubos JA, Nielsen J, Baker S (2011). Comparative genomics of citric-acid producing *Aspergillus niger* ATCC 1015 versus enzyme-producing CBS 513.88. Genome Res.

[CR14] Armstrong V, Cortes M, Lopez J (1996). A revision of the absolute configuration of drim-9(11)-en-8-alfa-ol. Nat Prod Lett.

[CR15] Asai Y, Nonaka N, Nishio M, Okamura K, Date T, Sugita T, Ohnuki T, Komatsubara S (1997). TMC-2A, -2B and -2C, new dipeptidyl peptidase IV inhibitors produced by *Aspergillus oryzae* A374. II. Isolation and structure elucidation. J Antibiot.

[CR16] Asao T, Büchi G, Abdel-Kader MM, Chang SB, Wick EL, Wogan GN (1963). Aflatoxins B and G. J Am Chem Soc.

[CR17] Asao T, Büchi G, Abdel-Kader MM, Chang SB, Wick EL, Wogan GN (1965). The structures of aflatoxins B and G_1_. J Am Chem Soc.

[CR18] Assante G, Camarda L, Locci R, Merlini L, Nasini G, Papadopoulos E (1981). Isolation and structure of red pigments from *Aspergillus flavus* and related species, growth on a differential medium. J Agric Food Chem.

[CR19] Atalla MM, Hassanein NM, El-Beih AA, Youssef YAG (2003). Mycotoxin production in wheat grains by different Aspergilli in relation to different relative humidities and storage periods. Nahrung.

[CR20] Ates S, Gökdere M (2006). Effect of silicone oil on gibberellic acid production by *Gibberella fujikuroi* and *Aspergillus niger*. Appl Biochem Microbiol.

[CR21] Avalos J, Limon MC (2015). Biological roles of fungal carotenoids. Curr Genet.

[CR22] Awakawa T, Yang X-L, Wakimoto T, Abe I (2013). Pyranonigrin E: a PKS-NRPS hybrid metabolite from *Aspergillus niger* identified by genome mining. ChemBioChem.

[CR23] Ayer WA, Nozawa K (1990). Taxonomy and chemistry of a new fungus from bark beetle infested *Pinus contorta var. latifolia*. 2. Arthrographol, the metabolite inhibitory to *Ophiostoma clavigerum*. Can J Microbiol.

[CR24] Baker SE (2006). *Aspergillus niger* genomics: past, present and into the future. Med Mycol.

[CR25] Baker SE, Perrone G, Richardson NM, Gallo A, Kubicek CP (2012). Phylogenomic analysis of polyketide synthase encoding genes in *Trichoderma*. Microbiology-SGM.

[CR26] Bakhali A, El-Samawaty AMA, Abd El-Rahim MA, Yassin MA, El-Naggar MA, Muhammed MH (2015) Toxigenic fungal biota associated with walnut in Saudi Arabia. J Pure Appl Microbiol 7:1079–1086

[CR27] Baltz RH, Demain AL, Davies JE (eds) (2010) Manual of industrial microbiology and biotechnology. 3rd ed, ASM Press, Washington DC

[CR28] Bandara HMSKH, Kumar NS, Jayasinghe L, Masubuti H, Fujimoto Y (2015). A 3-vinyl cephem derivative, a useful intermediate in the synthesis of cepham antibiotics, from *Aspergillus awamori* associated with banana fruit. Nat Prod Commun.

[CR29] Barbesgaard P, Heldt-Hansen HP, Diderichsen B (1992). On the safety of *Aspergillus oryzae*: a review. Appl Microbiol Biotechnol.

[CR30] Barnes CL, Steiner JR, Torres E, Pacheco R, Marquez H (1990). Structure and absolute configuration of pyrophen, a novel pyrone derivative of L-phenylalanine from *Aspergillus niger*. Int J Pept Prot Res.

[CR31] Bennett JW, Inamdar AA (2015). Are some fungal volatile organic compounds (VOCs) mycotoxins?. Toxins.

[CR32] Bentley R (2006). From *miso, sake* and *shoyu* to cosmetics: a century of science for kojic acid. Nat Prod Rep.

[CR33] Berg DH, Massing RP, Hoehn MM, Boeck LD, Hamill RL (1976). A30641, a new epidithiodiketopiperazine with antifungal activity. J Antibiot.

[CR34] Bertrand RL, Abdel-Hameed M, Sorensen JL (2018). Lichen biosynthetic gene clusters part II: homology mapping suggests a functional diversity. J Nat Prod.

[CR35] Bills Gerald F, Yue Qun, Chen Li, Li Yan, An Zhiqiang, Frisvad Jens C (2015). Aspergillus mulundensis sp. nov., a new species for the fungus producing the antifungal echinocandin lipopeptides, mulundocandins. The Journal of Antibiotics.

[CR36] Birkinshaw JH, Charles JHV, Lilly CH, Raistrick H (1931). The biochemistry of microorganisms VII. Kojic acid (5-hydroxy-2-hydroxymethylpyrone). Phil Trans R Soc London.

[CR37] Blin K, Wolf T, Chevrette MG, Lu X, Schwalen CJ, Kautsar SA, Duran HGS, de los Santos ELC, Kim HU, Nave M, Dickschat JS, Mitchell DA, Shelest E, Breitling R, Takano E, Lee SY, Weber T, Medema MH (2017). AntiSMASH 4.0—improvements in chemistry prediction and gene cluster boundary identification. Nucl Acids Res.

[CR38] Blochwitz A (1929). Die Gattung *Aspergillus*. Neue spezies. Diagnosen. Synonyme. Ann Mycol.

[CR39] Blumenthal CZ (2004). Production of toxic metabolites in *Aspergillus niger*, *Aspergillus oryzae* and *Trichoderma reesei*: justification of mycotoxin testing in food grade enzymes derived from the three fungi. Reg Toxicol Pharmacol.

[CR40] Bothast RJ, Fennell DI (1974). A medium for rapid identification and enumeration of *Aspergillus flavus* and related organisms. Mycologia.

[CR41] Bouras N, Mathieu F, Coppel Y, Lebrihi A (2005). Aurasperone F—a new member of the naphtho-gamma-pyrone class isolated from a cultures microfungus, *Aspergillus niger* C-433. Nat Prod Res.

[CR42] Bouras N, Mathieu F, Coppel Y, Strelkov SE, Lebrihi A (2007). Occurrence of naphtho-gamma-pyrones- and ochratoxin A-producing fungi in French grapes and characterization of new naphtho-gamma-pyrone polyketide (aurasperone G) isolated from *Aspergillus niger* C-433. J Agric Food Chem.

[CR43] Braun MS, Wink M (2018). Exposure, occurrence, and chemistry of fumonisins and their cryptic derivatives. Compr Rev Food Sci Food Saf.

[CR44] Brookes D, Tidd BK, Turner WB (1963). Avenaciolide, an antifungal lactone from *Aspergillus avenaceus*. J Chem Soc.

[CR45] Brown DW, Hauser FM, Tommasi R, Corlett SS, Salso JJ (2003). Structural elucidation of a putative conidial pigment from *Aspergillus parasiticus*. Tetrahedron Lett.

[CR46] Brückner H, Graf H (1983). Paracelsin, a peptide antibiotic containing α-aminobutyric acid, isolated from *Trichodeerma reesei* Simmons. Part A. Experientia.

[CR47] Brückner H, Graf H, Bokel M (1984). Paracelsin; characterization by NMR spectroscopy and circular dichroism, and hemolytic properties of a peptaibol antibiotic from the cellulolytically active mold *Trichoderma reesei*. Part B. Experientia.

[CR48] Bugni TS, Abbanat D, Bernan VS, Maiese WM, Gereenstein M, van Wagoner RM, Ireland CM (2000). Yanuthones: novel metabolites from a marine isolate of *Aspergillus niger*. J Org Chem.

[CR49] Burdock GA, Carabin IG, Madhusudan GS (2001). Safety assessment of β-nitropropionic acid: a monograph in support of an acceptable daily intake in humans. Food Chem.

[CR50] Burkhardt HJ, Forgacs J (1968). O-methylsterigmatocystin, a new metabolite from *Aspergillus flavus*, Link ex Fries. Tetrahedron.

[CR51] Bush MT, Goth A (1943). Flavicin: an antibacterial substance produced by an *Aspergillus flavus*. J Pharmacol Exptl Therap.

[CR52] Bush MT, Goth A, Dickison HL (1945). Flavicin II: an antibacterial substance produced by an *Aspergillus flavus*. J Pharmacol Exp Ther.

[CR53] Bush MT, Touster O, Brockman JE (1951). The production of β-nitropropionic acid by a strain of *Aspergillus flavus*. J Biol Chem.

[CR54] Caesar F, Jansson K, Mutschler E (1969). Über Nigragillin, ein neues Alkaloid aus der *Aspergillus niger*-Gruppe. 1. Mitteilung. Isolierung and Strukturaufklärung des Nigragillins und eines Dioxopiparazine. Pharm Acta Helv.

[CR55] Cairns TC, Nai C, Meyer V (2018). How a fungus shapes biotechnology: 100 years of *Aspergillus niger* research. Fung Biol Biotechnol.

[CR56] Capasso R, Iacobellis NS, Bottalico A, Randazzo G (1984). Structure-toxicity relationships of the eremophilane phomenone and PR-toxin. Phytochemistry.

[CR57] Cary JW, Ehrlich KC (2006). Aflatoxigenicity in *Aspergillus*: molecular genetics, phylogenetic relationship and evolutionary implications. Mycopathologia.

[CR58] Cary JW, Uka V, Han Z, Buyst D, Harris-Coward PY, Ehrlich KC, Wei Q, Bhatnagar D, Down PF, Martens SL, Calvo AM, Martins JC, Vanheaecke L, Coenye T, de Saeger S, di Mavungu JD (2015). An *Aspergillus flavus* secondary metabolic gene cluster containing a hybrid PKS-NRPS is necessary for synthesis of the 2-pyridones, leporins. Fung Genet Biol.

[CR59] Casadevall A, Pirofski L-A (2003). The damage-response framework of microbial pathogenesis. Nat Rev Microbiol.

[CR60] Cendoya E, Chiotta ML, Zachetti V, Chulze SN, Ramirez ML (2018). Fumonisins and fumonisin producing *Fusarium* occurrence in wheat and wheat by products: a review. J Cer Sci.

[CR61] Chang C.M, Chern J, Chen M-Y, Huang K-F, Chen CH, Yang YL, Wu SH (2015) Avenaciolides: potenatial Mur A-targeted inhibitors against peptidoglycan biosynthesis in methicillin-resistant *Staphylococcus aureus* (MRSA). J Am Chem Soc 137:267–275 10.1021/ja510375f 25521652

[CR62] Chang P-K, Wilkinson JR, Horn BW, Uy J, Bhatnagar D, Cleveland TE (2007). Genes differentially expressed by *Aspergillus flavus* strains after loss of aflatoxin production by serial transfers. Appl Microbiol Biotechnol.

[CR63] Chankhamjon P, Hoettger-Schmidt D, Scherlach K, Urbansky B, Lackner G, Kalb D, Dahse H-M, Hoffmeister D, Hertweck C (2014). Biosynthesis of the halogenated mycotoxin aspirochlorin in koji mold involves a cryptic amino acid conversion. Angew Chem Int Ed.

[CR64] Chen AJ, Hubka V, Frisvad JC, Visagie CM, Houbraken J, Meijer M, Varga J, Rasine D, Jurjević Ž, Kubátová A, Sklenář F, Samson RA (2017). Polyphasic taxonomy of *Aspergillus* section *Aspergillus* (formerly *Eurotium*) and its occurrence in indoor environment and food. Stud Mycol.

[CR65] Chiang Y-M, Meyer KM, Praseuth M, Baker SE, Bruno KS, Wang CCC (2011). Characterization of a polyketide synthase in *Aspergillus niger* whose product is a precursor for both dihydroxynaphthalene (DHN) melanin and naphtho-γ-pyrone. Fungal Genet Biol.

[CR66] Chidananda C, Kumar CM, Sattur AP (2008). Strain improvement of *Aspergillus niger* for the enhanced production of asperenone. Ind J Microbiol.

[CR67] Choque E, El Rayess Y, Reaynal J, Mathieu F (2015). Fungal naphtho-γ-pyrones—secondary metabolites of industrial interest. Appl Microbiol Biotechnol.

[CR68] Christensen M (1981). A synoptic key and evaluation of the species in the *Aspergillus flavus* group. Mycologia.

[CR69] Christensen BE, Mølgaard H, Kaasgaard S, Lehmbeck J, Mølgaard MDLH (2000) Producing a polypeptide of interest such as a hormone or enzyme, comprising cultivating a mutant of a parent Aspergillus cell which produces less of at least one toxin of interest compared to the parent cell under the same conditions. Patent, WO200039322, July 6.

[CR70] Cihangir N (2002). Stimulation of the gibberellic acid synthesis by *Aspergillus niger* in submerged culture using a precursor. World J Microbiol Biotechnol.

[CR71] Clevenger KD, Bok JW, Ye R, Miley GP, Verdan MH, Velk T, Chen C, Yang KH, Robey MT, Gao P, Lamprecht M, Thomas PM, Islam MN, Palmer JM, Wu CC, Keller NP (2017). A scalable platform to identify fungal secondary metabolites and the gene clusters. Nat Chem Biol.

[CR72] Cole RJ, Cox RH (1981). Handbook of toxic fungal metabolites.

[CR73] Cole RJ, Dorner JW, Springer JP, Cox RH (1981). Indole metabolites from a strain of *Aspergillus flavus*. J Agric Food Chem.

[CR74] Cole RJ, Kirksey JW, Schroeder HW (1970). Dihydro-O-methylsterigmatocystin, a new metabolite from *Aspergilus flavus*. Tetrahedron Lett.

[CR75] Costa CP, Silva DG, Rudnitskaya A, Almeida A, Rocha SM (2016). Shedding light on *Aspergillus niger* exometabolome. Sci Rep.

[CR76] Curtis RW, Tanaka H (1967). Production of malformin by *Aspergillus awamori*. Appl Microbiol.

[CR77] Cutler HG, Crumley FG, Cox RH, Hernandez O, Cole RJ, Dorner JW (1979). Orlandin: a nontoxic fungal metabolite with plant growth inhabiting properties. J Agric Food Chem.

[CR78] Daengrot C, Rukachairisikul V, Tansakul C, Thongpanchang T, Phongpaichit S, Bowornwiriyapan K, Sakayaroj J (2015). Eremophilane sesquiterpenes and diphenyl thioethers from the soil fungus *Penicillium copticola* PSU-RSPG138. J Nat Prod.

[CR79] Dey NC (1945). A preliminary note on the antibacterial substance from *Aspergillus flavus*. Curr Sci (India).

[CR80] Dickens F, Jones HEH (1961). Carcinogenic activity of a series of reactive lactones and related substances. Br J Cancer.

[CR81] Ding G, Jiang L, Guo L, Chen X, Zhang H, Che Y (2008). Pestalazines and pestalamides, bioactive metabolites from the plant pathogenic fungus *Pestalotiopsis theae*. J Nat Prod.

[CR82] Domíngues G, Hueso-Rodríguez JA, de la Torre MC, Rodríguez B (1991). Synthesis and absolute configuration of drim-9(11)-en-8-ols from *Aspergillus oryzae*. Tetrahedron Lett.

[CR83] Domsch KH, Gams W, Anderson T-H (2007). Compendium of soil fungi.

[CR84] Dunn G, Newbold GT, Spring FS (1949). Synthesis of flavacol, a metabolic product of *Aspergillus flavus*. J Chem Soc.

[CR85] Duran RM, Cary JW, Calvo AM (2007). Production of cyclopiazonic acid, aflatrem, and aflatoxin by *Aspergillus flavus* is regulated by *veA*, a gene necessary for sclerotial formation. Appl Microbiol Biotechnol.

[CR86] Dutcher JD (1947). Aspergillic acid: an antibiotic substance produced by *Aspergillus flavus*. I. General properties, formastion of desoxyaspergillic acid and structural conclusions. J Biol Chem.

[CR87] Dutcher JD (1958). Aspergillic acid: an antibiotic substance produced by *Aspergillus flavus*. III. The structure of hydroxyaspergillic acid. J Biol Chem.

[CR88] Dutton MF, Heathcote JG (1968). Structure biochemical properties and origin of aflatoxins B_2a_ and G_2a_. Chem Ind.

[CR89] Ehrlich KC, Mack BM (2014). Comparison of expression of secondary metabolite biosynthesis cluster genes in *Aspergillus flavus*, *A. parasiticus*, and *A. oryzae*. Toxins.

[CR90] El-Hag N, Morse RE (1976). Aflatoxin production by a variant of *Aspergillus oryzae* (NRRL strain 1988) on cowpeas (*Vigna sinensis*). Science.

[CR91] El-Kady I, El-Maraghy S, Zohri A (1994). Mycotoxin producing potential of some isolates of *Aspergillus flavus* and *Eurotium* groups from meat-products. Microbiol Res.

[CR92] Ellis JJ, Stodola FH, Vesonder RF, Glass CA (1964). A C_15_H_22_O_4_ compound produced by the fungus *Aspergillus fischeri* var. *glaber*. Nature.

[CR93] Elsahrkawy SH, Abbas HK (1991). Metabolism of T-2 toxin by *Mucor* and *Aspergillus* sp. Acta Pharm Jugos.

[CR94] El-Sayed Ashraf S.A., Safan Samia, Mohamed Nabil Z., Shaban Lamis, Ali Gul Shad, Sitohy Mahmoud Z. (2018). Induction of Taxol biosynthesis by Aspergillus terreus , endophyte of Podocarpus gracilior Pilger, upon intimate interaction with the plant endogenous microbes. Process Biochemistry.

[CR95] Enomoto M, Saito M (1972). Carcinogens produced by fungi. Annu Rev Microbiol.

[CR96] Esaki H, Onozaki H, Kawakishi S, Ozawa T (1996). New antioxidant isolated from tempeh. J Agric Food Chem.

[CR97] Evidente A, Cristinzio G, Punzo B, Andolfi A, Testa A, Melck D (2009). Flufuran, an antifungal 3,5-disubstituted furan produced by *Aspergillus flavus* Link. Chem Biodiver.

[CR98] Faustinelli PC, Wang XM, Palencia ER, Arias RS (2016). Genome sequences of eight *Aspergillus flavus* spp., and one *A. parasiticus* sp., isolated from peanut seeds in Georgia. Genome Announc.

[CR99] Fennell DI (1976). *Aspergillus oryzae* (NRRL strain 1988): a clarification. Science.

[CR100] Fernagut PO, Diguet E, Stefanova N, Biran M, Wenning GK, Canioni P, Bioulac B, Tison F (2002). Subacute systemic 3-nitropropionic acid intoxication induced a distinct motor disorder in adult C57B1/6 mice: behavioural and histopathological characterization. Neuroscience.

[CR101] Foster JW, Karow EO (1945). Microbiological aspects of penicillin. VIII. Penicillin from different fungi. J Bacteriol.

[CR102] Frisvad JC (1989). The connection between the penicillia and aspergilli and mycotoxins with special emphasis on misidentified isolates. Arch Environ Contam Toxicol.

[CR103] Frisvad JC, De Saeger S (2011). Rational for a polyphasic approach in the identification of mycotoxigenic fungi. Determining mycotocins and mycotoxigenic fungi in food and feed.

[CR104] Frisvad JC, Hubka V, Ezekiel CN, Hong S-B, Nováková A, Chen AL, Arzanlou M, Larsen TO, Sklenár F, Mahakarnchanakul W, Samson RA, Houbraken J (2019). Taxonomy of *Aspergillus* section *Flavi* and their production of aflatoxins, ochratoxins and other mycotoxins. Stud Mycol.

[CR105] Frisvad JC, Larsen TO (2015). Chemodiversity in the genus *Aspergillus*. Appl Microbiol Biotechnol.

[CR106] Frisvad JC, Larsen TO, Dalsgaard PW, Seifert KA, Louis-Seize G, Lyhne EK, Jarvis BB, Fettinger JC, Overy DP (2006). Four psychrotolerant species with high chemical diversity consistently producing cycloaspeptide A, *P. jamesonlandense* sp. nov., *P. ribium* sp. nov., *P. soppii* and *P. lanosum*. Int J Syst Evol Microbiol.

[CR107] Frisvad JC, Larsen TO, Thrane U, Meijer M, Varga J, Samson RA, Nielsen KF (2011). Fumonisin and ochratoxin production in industrial *Aspergillus niger* strains. PLoS ONE.

[CR108] Frisvad JC, Petersen LM, Lyhne EK, Larsen TO (2014). Formation of sclerotia and production of indoloterpenes by *Aspergillus niger* and other species in section *Nigri*. PLoS ONE.

[CR109] Frisvad JC, Smedsgaard J, Larsen TO, Samson RA (2004). Mycotoxins, drugs and other extrolites produced by species in *Penicillium* subgenus *Penicillium*. Stud Mycol.

[CR110] Frisvad JC, Smedsgaard J, Samson RA, Larsen TO, Thrane U (2007). Fumonisin B_2_ production by *Aspergillus niger*. J Agric Food Chem.

[CR111] Fu Y, He F, Zhang S, Jiao X (1995). Consistent striatal damage in rats induced by 3-nitropropionic acid and cultures of *Arthrinium* fungus. Neurotoxicol Teratol.

[CR112] Fujii R, Ugai T, Ichonose H, Hatakeyama M, Kosaki T, Gomio K, Fujii I, Minami A, Oikawa H (2016). Reconstitution of biosynthetic intermediates by expression host. Biosci Biotechnol Biochem.

[CR113] Fujimoto Y, Miyagawa H, Tsurushima T, Irie H, Okamura K, Ueno T (1993). Structures of antafumicins A and B, novel antifungal substances produced by the fungus *Aspergillus niger* NH-401. Biosci Biotechol Biochem.

[CR114] Fukuda T, Hasegawa Y, Hagimori K, Yamaguchi Y, Masuma R, Tomoda H, Omura S (2006). Tensidols, new potentiators of antifungal miconazol activity, produced by *Aspergillus niger* FKI-2342. J Antibiot.

[CR115] Fukui S, Tani Y, Kishibe T (1955). The role of B vitamins in sake brewing, II, The nature of a growth-stimulating factor produced by *Aspergillus oryzae* for *Saccharomyces sake* and its relationship to pantothenic acid. J Ferment Technol.

[CR116] Fukui S, Tani Y, Kishibe T (1955). The role of B vitamins in sake brewing, IV. Biosynthesis and decompositions of B vitamins by *Saccharomyces sake* and *Aspergillus oryzae*. J Ferment Technol.

[CR117] Galagan JE, Calvo SE, Cuomo C, Ma L-J, Wortman JR, Batzoglou S, Lee S-I, Bastürkmen M, Spevak CC, Clutterbuck J, Kapitonov V, Jurka J, Scazzocchio C, Farman M, Butler J, Purcell S, Harris S, Braus GH, Draht O, Busch S, D’Enfert C, Bouchier C, Goldman GH, Bell-Pedersen D, Griffith-Jones S, Doonan JH, Yu J, Vienken K, Pain A, Freitag M, Selker EU, Archer DB, Penalva MA, Oakley BR, Momany M, Tanaka T, Kumagai T, Asai K, Machida M, Nierman WC, Denning DW, Caddick M, Hynes M, Paoletti NM, Fischer R, Miller B, Dyer P, Sachs MS, Osmani SA, Birren BW (2005). Sequencing of *Aspergillus nidulans* and comparative analysis with *A. fumigatus* and *A. oryzae*. Nature.

[CR118] Gallagher RT, Clardy J, Wilson BJ (1980). Aflatrem, a tremorgenic toxin from *Aspergillus flavus*. Tetrahedron Lett.

[CR119] Gallagher RT, McCabe T, Hirotsu K, Clardy J, Nicholson J, Wilson BJ (1980). Aflavinine, a novel indole-mevanolate metabolite from tremorgen-producing *Aspergillus flavus* species. Tetrahedron Lett.

[CR120] Gallagher RT, Richard JL, Stahr HM, Cole RJ (1978). Cyclopiazonic acid production by aflatoxigenic and non-aflatoxigenic strains of *Aspergillus flavus*. Mycopathologia.

[CR121] Gallagher RT, Wilson BJ (1980). Aflatrem, the tremorgenic mycotoxin from *Aspergillus flavus*. Mycopathologia.

[CR122] Gallo A, Ferrara M, Perrone G (2017). Recent advances on the molecular aspects of ochratoxin A biosynthesis. Curr Op Food Sci.

[CR123] Gardner SN, Slezak T, Hall BG (2015). kSNP3.0: SNP detection and phylogenetic analysis of genomes without genome alignment or reference genome. Bioinformatics.

[CR124] Garson MJ, Jenkins SM, Staunton J, Chaloner PA (1986). Isolation of some new 3,6-dialkyl-1,4-dihydroxypiperazine-2,5-diones from *Aspergillus terreus*. J Chem Soc Perkin Trans I.

[CR125] Geiser DM, Dorner JW, Horn BW, Taylor JW (2000). The phylogenetics of mycotoxin and sclerotium production in *Aspergillus flavus* and *Aspergillus oryzae*. Fungal Genet Biol.

[CR126] Geiser DM, Pitt JI, Taylor JW (1998). Cryptic speciation and recombination in the aflatoxin-producing fungus *Aspergillus flavus*. Proc Nat Acad Sci USA.

[CR127] Georgianna D, Fedorova ND, Yu J, Machida M, Rokas A, Baker S, Dean R, Brown D, Dolexal A, Bhatnagar D, Cleveland T, Wortman J, Maiti R, Joardar V, Amedeo P, Denning D, Woloshuk C, Nierman W, Payne G (2009). Comparative genomics of *Aspergillus flavus* and *A. oryzae* revealed nearly identical genomes but differences in gene expression. Phytopathol.

[CR128] Ghosal S, Boswas K, Chakrabarti DK (1979). Toxic naphtho-γ-pyrones from *Aspergillus niger*. J Agric Food Chem.

[CR129] Gibbons JG, Salichos L, Slot JC, Rinker DC, McGary KL, King JG, Klich MA, Tabb DL, McDonald WH, Rokas A (2012). The evolutionary imprint of domestication on genome variation and function of the filamentous fungus *Aspergillus oryzae*. Curr Biol.

[CR130] Gilbert MK, Mack BM, Moore GG, Downey DL, Lebar MD, Joarder V, Losada L, Yu JJ, Nierman WC, Bhatnagar D (2018). Whole genome comparison of *Aspergillus flavus* L-morphotype strain NRRL 3357 (type) and S-morphotype strain AF70. PLoS ONE.

[CR131] Gilbert MK, Mack BM, Wei Q, Bland JM, Bhatnagar D, Cary JW (2016). RNA sequencing of an *nsdC* mutant reveals global regulation of secondary metabolite clusters in *Aspergillus flavus*. Microbiol Res.

[CR132] Gill-Serna J, García-Díaz M, González-Jaén M, Vázquez C, Patino B (2018). Description of an orthologous cluster of ochratoxin A biosynthetic genes in *Aspergillus* and *Penicillium* species. Int J Food Microbiol.

[CR133] Girol CG, Fisch KM, Heinekamp T, Günther S, Hüttel W, Piel J, Brakhage AA, Müller M (2012). Regio- and stereoselective oxidative phenol coupling in *Aspergillus niger*. Angew Chem Int Ed.

[CR134] Gloer JB, TePaske MR, Sima JS, Wicklow DT, Dowd PF (1988). Antiinsectan aflavinine derivatives from the sclerotia of *Aspergillus flavus*. J Org Chem.

[CR135] Godet M, Munaut F (2010). Molecular strategy for identification in *Aspergillus* section *Flavi*. FEMS Microbiol Lett.

[CR136] Goris J, Konstantinidis KT, Klappenbach JA, Coenye T, Vandamme P, Tiedje JM (2007). DNA-DNA hybridization values and their relationship to whole-genome sequence similarities. Int J Syst Evol Microbiol.

[CR137] Gould RO, Simpson TJ, Walkinshaw MD (1981). Isolation and X-ray crystal structures of astellolides A and B, sesquiterpenoid metabolites of *Aspergillus variecolor*. Tetrahedron Lett.

[CR138] Grove JF (1972). New metabolic products of *Aspergillus flavus*. Part. I. Asperentin, its methyl esters, and 5′-hydroxyasperentin. J Chem Soc Perkin Trans.

[CR139] Grove JF (1972). New metabolic products of *Aspergillus flavus*. Part. II. Asperflavin, anhydroasperflavin, and 5,7-dihydroxy-4-methylphthalide. J Chem Soc Perkin Trans.

[CR140] Grove JF (1973). New metabolic products of *Aspergillus flavus*. Part. III. Biosynthesis of asperentin. J Chem Soc Perkin Trans.

[CR141] Grove JF (1973). New metabolic products of *Aspergillus flavus*. Part. IV. 4′-Hydroxyasperentin and 5′-hydroxyasperentin 8-methyl ether. J Chem Soc Perkin Trans.

[CR142] Guida VO (1948). Activades antibioticas do *Aspergillus flavus*. Sobre diversas bacterias. Bol Soc Paulista Med Vet (Sao Paolo).

[CR143] Gurupavithra S, Rajalakshmi A, Jayachitra A (2017). Optimization of fermentation conditions for red pigment production from *Aspergillus flavus* under submerged cultivation analyse its antioxidant properties. Indo Am J P Sci.

[CR144] Haenni AL, Lederer E, Barbier M (1962) Chimique biologique - sur la structure de l'aspergillomarasmine A. Compt Rend Hebd Scean Acad Sci 255:1476–1480

[CR145] Haenni AL, Robert M, Vetter W, Roux L, Barbier M, Lederer E (1965). Structure chimique des aspergillomarasmins A et B. Helv Chim Acta.

[CR146] Hamasaki T, Kuwano H, Isono K, Hatsuda Y, Fukuyama K, Tsukihara T, Katsube Y (1975) New metabolite parasiticolide A, from *Aspergillus parasiticus*. Agric Biol Chem 39:749–751

[CR147] Hamsa TAP, Ayres JC (1977). A differential medium for the isolation of *Aspergillus flavus* from cottonseed. J Food Sci.

[CR148] Han X-M, Jiang HR, Xu J, Zhang J, Li F-Q (2017). Dynamic fumonisin B2 production by *Aspergillus niger* intended used in food industry in China. Toxins.

[CR149] Hanaka T, Ejiri K, Yamamoto H (2012). First synthesis of a natural isoxanthopterin glycoside, asperopterin A. Heterocycles.

[CR150] Hanaka T, Yamamoto H (2013). First synthesis of asperopterin A, an isoxanthopterin glycoside from *Aspergillus oryzae*. Pteridines.

[CR151] Hanson JR (2008). The chemistry of fungi.

[CR152] Happi GM, Kouam SF, Talontsi FM, Nkenfou CN, Longo F, Zuhlke S, Dounla-Meli C, Spiteller M (2015). A new dimeric naphtho-gamma-pyrone from an endophytic fungus *Aspergillus niger* AKRN associated with the roots of *Entandrophragma congoense* collected in Cameroun. Z Naturforsch Sect B J Chem Sci.

[CR153] Hardegger E, Liecht P, Jackman LM, Boller A, Plattner PA (1963). Welkstoffe und Antibiotika. 24. Mitteilung. Die Konstitution des Lycomarasmins. Helv Chim Acta.

[CR154] Hartley RD, Nesbitt BF, O’Kelley J (1963). Toxic metabolites of *Aspergillus flavus*. Nature.

[CR155] Hasegawa Y, Fukuda T, Hagimori K, Tomoda H, Omura S (2007). Tensyic acids, new antibiotics produced by *Aspergillus niger* FKI-2342. Chem Pharm Bull.

[CR156] Hashimoto M, Nonaka T, Fujii I (2014). Fungal type III polyketide synthases. Nat Prod Rep.

[CR157] Hashimoto M, Seshime Y, Kitamoto K, Uchhiyama N, Goda Y, Fujii I (2013). Identification of csypyrone B2 and B3 as the minor products of *Aspergillus oryzae* type III polyketide synthase CsyB. Biomed Med Chem Lett.

[CR158] Hautbergue T, Puel O, Tadrist S, Meneghetti L, Pean M, Delaforge M, Debrauwer L, Oswald IP, Jamin EL (2017). Evidencing 98 secondary metabolites of *Penicillium verrucosum* using substrate isotopic labelling and high-resolution mass spectrometry. J Chromatogr B Anal Technol Biomed Life Sci.

[CR159] He Y, Tain J, Chen X, Sun W, Zhu H, Li Q, Lei L, Yao G, Xue Y, Wang J, Li H, Zhang Y (2016). Fungal naphtho-γ-pyrones: potent antibiotics for drug-resistant microbial pathogens. Sci Rep.

[CR160] He Y, Wang B, Chen W, Cox RJ, He J, Chen F (2018). Recent advances in reconstructing microbial secondary metabolites biosynthesis in *Aspergillus* spp. Biotechnol Adv.

[CR161] He FS, Zhang S, Qian FY, Zhang CL (1995). Delayed dystonia with striatal CT lucencies induced by a mycotoxin (3-nitropropionic acid). Neurology.

[CR162] Heathcote JG, Dutton MF (1969). New metabolites of *Aspergillus flavus*. Tetrahedron.

[CR163] Henrikson JC, Ellis TK, King JB, Cichewicz RH (2011). Reappraising the structures and distribution of metabolites from the black aspergilli containing uncommon 2-benzyl-4H-pyran-4-one and 2-benzylpyridin-4(1H)-one systems. J Nat Prod.

[CR164] Henrikson JC, Hoover AR, Joyner PM, Cichewicz RH (2009). A chemical epigenetics approach for engineering the in situ biosynthesis of a cryptic natural product from *Aspergillus niger*. Org Biomol Chem.

[CR165] Henry MH, Wyatt RD (2001). The toxicity of fumonisin B_1_, B_2_ and B_3_, individually and in combination, in chicken embryos. Poultry Sci.

[CR166] Hesseltine CW, Shotwell OL, Smith M, Ellis JJ, Vandegraft E, Shannon G (1970). Production of various aflatoxins by strains of the Aspergillus flavus series. In: Herzberg M (ed) Proceedings of the first joint U.S.–Japan conference on toxic micro-organisms. Mycotoxins. Botulism. UJNR Joint Panels on Toxic Micro-organisms and the U.S. Department of the Interior, Washington D.C., USA, 202–210

[CR167] Heussner Alexandra, Bingle Lewis (2015). Comparative Ochratoxin Toxicity: A Review of the Available Data. Toxins.

[CR168] Higuchi R (1956). Biosynthesis and destruction of flavin mononucleotide and flavine adenine dinucleotide by *Aspergillus oryzae*. I. Comparison of riboflavine and its derivatives biosynthesized by some species of *Aspergillus oryzae*. Vitamins (Kyoto).

[CR169] Hiort J, Maksimenka K, Reichert M, Perović-Ottstadt S, Lin WH, Wray V, Steube K, Schaumann K, Weber H, Proksch P, Ebel R, Müller WEG, Bringmann G (2004). New natural products from the sponge-derived fungus *Aspergillus niger*. J Nat Prod.

[CR170] Holker JSE, Kagal SA, Mulheirn LJ, White PM (1966). Some new metabolites of *Aspergillus versicolor* and a revised structure for averufin. J Chem Soc Chem Commun.

[CR171] Holm DK, Petersen LM, Klitgaard A, Knudsen PB, Jarzynska ZD, Nielsen KF, Gotfredsen CH, Larsen TO, Mortensen UH (2014). Molecular and chemical characterization of the biosynthesis of the 6-MSA-derived meroterpenoid yanuthone D in *Aspergillus niger*. Chem Biol.

[CR172] Holzapfel Cedric W., Bredenkamp Martin W., Snyman Renske M., Boeyens Jan C.A., Allen Christine C. (1990). Cyclopiamide, an isoindolo[4,6-cd]indole from Penicillium cyclopium. Phytochemistry.

[CR173] Hong S-B, Lee M, Kim D-H, Chung S-H, Shin H-D, Samson RA (2013). The proportion of non-aflatoxigenic strains of the *Aspergillus flavus/oryzae* complex from Meju by analyses of the aflatoxin biosynthetic genes. J Microbiol.

[CR174] Hong S-B, Lee MM, Kim D-H, Varga J, Frisvad JC, Perrone G, Gomi K, Yamada O, Machida M, Houbraken J, Samson RA (2013). *Aspergilllus luchuensis*, an industrially important black *Aspergillus* in East Asia. PLoS ONE.

[CR175] Horn BW, Dorner JW (2001). Effect of competition and adverse culture conditions on aflatoxin production by *Aspergillus flavus* through successive generations. Mycologia.

[CR176] Houbraken J, de Vries RP, Samson RA (2014). Modern taxonomy of biotechnologically important *Aspergillus* and *Penicillium* species. Adv Appl Microbiol.

[CR177] Hu X, Xia Q-W, Zhao Y-Y, Zheng Q-H, Liu Q-Y, Chen L, Zhang Q-Q (2014). Speradines B-E, four novel tetracyclic oxindole alkaloids from the marine-derived fungus *Aspergillus oryzae*. Heterocycles.

[CR178] Hu X, Xia Q-W, Zhao Y-Y, Zheng Q-H, Liu Q-Y, Chen L, Zhang Q-Q (2014). Speradines F-H, three new oxindole alkaloids from the marine-derived fungus *Aspergillus oryzae*. Chem Pharm Bull.

[CR179] Hummel BCW (1956). Isolation and partial characterization of flavicidic acid, phytotoxic metabolite of *Aspergillus flavus*. Dissertation Abstr.

[CR180] Huson DH, Scornavacca C (2012). Dendroscope 3: an interactive tool for rooted phylogenetic trees and networks. Syst Biol.

[CR181] Hüttel W, Müller M (2007). Regio- and steroselective intermolecular oxidative phenol coupling in kotanin biosynthesis by *Aspergillus niger*. ChemBioChem.

[CR182] Iizuka A, Iida M (1962). Maltoryzin, a new toxic metabolite produced by a strain of *Aspergillus oryzae* var. *microsporus* isolated from the poisonous malt sprout. Nature.

[CR183] Inokoshi J, Shiomi K, Masuma R, Tanaka H, Yamada H, Omura S (1999). Funalenone, a novel collagenase inhibitor produced by *Aspergillus niger*. J Antibiot.

[CR184] Itoh Y, Takahashi S, Haneishi T, Arai M (1989). Structure of heptelidic acid, a new sesquiterpene antibiotic from fungi. J Antibiot.

[CR185] Iwamoto T, Hirota A, Shima S, Sakai H, Isogai A (1985). Nigerazine A, an isomer of nigerazine B, from *Aspergillus niger*. Agric Biol Chem.

[CR186] Iwamoto T, Shima S, Hirota A, Isogai A, Sakai H (1983). Nigerazine B, a new metabolite from *Aspergillus niger* screening, isolation, and chemical and biological properties. Agric Biol Chem.

[CR187] Iwasaki T, Kosikowski FV (1973). Production of β-nitropropionic acid in foods. J Food Sci.

[CR188] Jahn F (1977). Toxicity and production of oxalic acid of an *Aspergillus niger* strain from mold hay. Wien Tierartl Monatschr.

[CR189] Jansen BJM, de Groot A (1990). The occurrence and biological activity of drimane sesquiterpenoids. Nat Prod Rep.

[CR190] Jansen BJM, de Groot A (2004). The occurrence and biological activity of drimane sesquiterpenoids. Nat Prod Rep.

[CR191] Jarvis BB, Miller JD (2005). Mycotoxins as harmful indoor air contaminants. Appl Microbiol Biotechnol.

[CR192] Jefferson WE (1967). Steroids and other factors influencing the accumulation of asperenone and fermentation acids by *Aspergillus niger* in replacement cultures. Biochem.

[CR193] Jefferson WE (1967). The isolation and characterization of asperenone, a new phenylpolyene from *Aspergillus niger*. Biochemistry.

[CR194] Jennings MA, Williams TI (1945). Production of kojic acid by *Aspergillus effusus* Tiraboschi. Nature.

[CR195] Johnson JR, Robinson BL, Ali SF, Binienda Z (2000). Dopamine toxicity following long term exposure to low doses of 3-nitropropionic acid (3-NPA) in rats. Toxicol Lett.

[CR196] Jørgensen TR (2007). Identification and toxigenic potential of the industrally important fungi, *Aspergillus oryzae* and *Aspergillus sojae*. J Food Prot.

[CR197] Jørgensen TR, Park J, Arentshorst M, van Welzen AM, Lamers G, vanKuyk PA, Damvel RA, van den Hondel CAM, Nielsen KF, Frisvad JC, Ram AFJ (2011). The molecular and genetic basis of conidial pigmentation in *Aspergillus niger*. Fungal Gen Biol.

[CR198] Juvvadi PR, Seshime Y, Kitamoto K (2005). Genomics reveal traces of fungal phenylpropanoid-flavanoid metabolic pathway in the filamentous fungus *Aspergillus oryzae*. J Microbiol.

[CR199] Kaneko Y, Sanada M (1969). Studies on the fluorescent substances produced by *Aspergillus* fungi. VIII. Purification and isolation of asperopterin B and chemical properties of asperopterin B and A. J Ferment Technol.

[CR200] Kang D, Kim J, Choi JN, Liu K-H, Lee CH (2011). Chemotaxonomy of *Trichoderma* spp. using mass spectrometry metabolite profiling. J Microbiol Biotechnol.

[CR201] Kato A, Saeki T, Suzuki S, Ando K, Tamura G, Arima K (1969). Oryzachlorin, a new antifungal antibiotic (Studies on antiviral and antitumor Antibiotics XVIII). J Antibiot.

[CR202] Kato N, Tokuoka M, Shinohara Y, Kaeatani M, Uramoto M, Seshime Y, Fujii I, Kitamoto K, Takahashi T, Takahashi S, Koyama Y, Osada H (2011). Genetic safeguard against mycotoxin cyclopiazonic acid production in *Aspergillus oryzae*. ChemBioChem.

[CR203] Kayoko M (1985). Reduction of ochratoxin a toxicity in mice treated with phenylalanine and phenobarbital. Toxicol Lett.

[CR204] Kim JH, Lee CH (2009). Heptelidic acid, a sesquiterpene lactone, inhibits apoptosis in human leukemia U937 cells. J Microbiol Biotechnol.

[CR205] Kim K-W, Sugawara F, Yoshida S, Murofuschi N, Takahashi N, Curtis RW (1993). Structure of malformin B, a phytotoxic metabolite produced by *Aspergillis niger*. Biosci Biotech Biochem.

[CR206] Kimura Y, Nishibe M, Nakajima H, Hamasaki T, Shigemitsu N, Sugawara F, Stout TJ, Clardy J (1992). Emeniveol; a new pollen growth inhibitor from the fungus, *Emericella nivea*. Tetrahedron Lett.

[CR207] King AM, Reid-Yu SA, Wang W, King DT, De Pascale G, Strynadka NC, Walsch TR, Coombes BK, Wright GD (2014). Aspergillomarasmine A overcomes metallo-β-lactamase antibiotic resistance. Nature.

[CR208] Kinoshita S, Akita S, Saito K (1961) L-glutamic acid. Japanese patent no. 3171, Apr. 14.

[CR209] Kishimoto S, Tsunematsu Y, Sato M, Watanabe K (2017). Elucidation of biosynthetic pathways of natural products. Chem Rec.

[CR210] Kistner HE Sr (1962) Sucrose fermentation of *Aspergillus flavus-oryzae*. U.S. patent no. 3,043,748, July 10.

[CR211] Kiyota T, Hamada R, Sakamoto K, Iwashita K, Yamada O, Mikami S (2011). Aflatoxin non-productivity of *Aspergillus oryzae* caused by loss of function of the *aflJ* gene product. J Biosci Bioeng.

[CR212] Kjærbølling I, Vesth TC, Frisvad JC, Nybo JL, Theobald S, Kuo A, Bowyer P, Matsuda Y, Mondo S, Lyhne EK, Kogle M, Clum A, Lipzen A, Salamov A, Ngan C, Daum C, Chiniquy J, Barry K, LaButti K, Haridas S, Simmons B, Magnuson J, Mortensen U, Larsen T, Grigoriev I, Baker S, Andersen MR (2018). Linking secondary metabolites to gene clusters through genome sequencing of six diverse *Aspergillus* species. Proc Nat Acad Sci, USA.

[CR213] Klausmeyer P, McCloud TG, Tucker KD, Cardellina JH, Shoemaker RH (2005). Aspirochlorine class compounds from *Aspergillus flavus* inhibit azole-resistant *Candida albicans*. J Nat Prod.

[CR214] Klich MA, Mullaney EJ (1987). DNA restriction enzyme gragment polymorphiosms as a tool for rapid differentiation of *Aspergillus flavus* from *Aspergillus oryzae*. Exp Mycol.

[CR215] Klich MA, Pitt JI, Samson RA, Pitt JI (1985). The theory and practice of distinguishing species of the *Aspergillus flavus* group. Advances in *Penicillium* and *Aspergillus* systematics.

[CR216] Klich MA, Pitt JI (1988). Differentiation of *Aspergillus flavus* from *A. parasiticus* and other closely related species. Trans Brit Mycol Soc.

[CR217] Klitgaard A, Nielsen JB, Frandsen RJN, Andersen MR, Nielsen KF (2015). Combining stabel isotope labelling and molecular networking for biosynthetic pathway characterization. Anal Chem.

[CR218] Kobayashi T (1966). Food poisoning. Chiba Daikage Fuhai Kenkyosho Hokoku.

[CR219] Kobayashi T, Abe K, Asai K, Gomi K, Juvvadi PR, Kato M, Kitamoto K, Takeuchi M, Machida M (2007). Genomics of *Aspergillus oryzae*. Biosci Biotechnol Biochem.

[CR220] Kobayashi K, Shimizu H, Itho M, Suginome H (1990). An efficient synthesis of 5,7-dihydroxy-4-methylisobenzofuran-1-(3H)-one, a metabolite of *Aspergillus flavus* and a key intermediate in the synthesis of mycophenolic acid. Bull Chem Soc Japan.

[CR221] Kobbe B, Cushman M, Wogan GN, Demain AL (1977). Production and bacterial activity of malformin C, a toxic metabolite of *Aspergillus niger*. Appl Environ Microbiol.

[CR222] Koch L, Lodin A, Herold I, Ilan M, Carmeli S, Yarden O (2014). Sensitivity of *Neurospora crassa* to a marine-derived *Aspergillus tubingensis* anhydride exhibiting antifungal activity that is mediated by the MAS1 protein. Mar Drugs.

[CR223] Kocsubé S, Perrone G, Magistà D, Houbraken J, Varga J, Szigeti G, Hubka V, Hong S-B, Frisvad JC, Samson RA (2016). *Aspergillus* is monophyletic: evidence from multiple gene phylogenies and extrolite profiles. Stud Mycol.

[CR224] Kodukula K, Arcuri M, Cutrone JQ, Hugill RM, Lowe SE, Pirnik DM, Shu Y-Z, Fernandes PV, Seethala R (1995). BMS-192548, a tetracyclic binding inhibitor of neuropeptide Y receptors, from *Aspergillus niger* WB2346. II. Taxonomy, fermentation, isolation and biological activity. J Antibiot.

[CR225] Koiso Y, Iwasaki S, Hanaoka K, Kobayashi T, Sonoda R, Fujita Y, Yaegashi H, Sato Z (1994). Ustiloxins, antimitotic cyclic peptides from false smut balls on rice panicles caused by *Ustilaginoidea virens*. J Antibiot.

[CR226] Koiso Y, Natori M, Iwasaki S, Sato S, Sonoda R, Fujita Y, Yaegashi H, Sato Z (1992). Ustiloxin: a phytotoxin and a mycotoxin from false smut balls on rice panicles. Tetrahedron Lett.

[CR227] Komagata D, Fujita S, Yamashita N, Saito S, Morino T (1996). Novel neuritogenic activities of pseurotin A and penicillic acid. J Antibiot.

[CR228] Koteva K, King AM, Capretta A, Wright GD (2016). Total synthesis and activity of the metallo-β-lactamase inhibitor aspergillomarasmin A. Angew Chem Ind Ed.

[CR229] Kozakiewicz Z, Frisvad JC, Hawksworth DL, Pitt JI, Samson RA, Stolk AC (1992). Proposals for nomina specifica conservanda and rijicienda in *Aspergillus* and *Penicillium* (Fungi). Taxon.

[CR230] Kőszegi Tamás, Poór Miklós (2016). Ochratoxin A: Molecular Interactions, Mechanisms of Toxicity and Prevention at the Molecular Level. Toxins.

[CR231] Kredics László, Varga János, Antal Zsuzsanna, Samson Robert A, Kocsubé Sándor, Narendran Venkatapathy, Bhaskar Madhavan, Manoharan Chockaiya, Vágvölgyi Csaba, Manikandan Palanisamy (2008). Black aspergilli in tropical infections. Reviews in Medical Microbiology.

[CR232] Kozlovskii AG, Solov’yeva TF, Bukhtiyarov YE, Shurukhin YV, Sakharovskii VG, Adanin VM, Nefedova MY, Pertsova RN, Tokarev VG, Golovleva LE (1990). Secondary metabolites of new soil strains of microscopic fungi from the genera *Aspergillus* and *Penicillium*. Microbiology (Moscow).

[CR233] Kubicek CP, Druzhinina IS, Paterson RRM, Lima N (2016). *Trichoderma* mycoses and mycotoxins. Molecular biology of food and water borne mycotoxigenic and mycotic fungi.

[CR234] Kupfahl C, Michalka A, Lass-Flörl C, Fischer G, Haase G, Ruppert T, Geginat G, Hof H (2008). Gliotoxin production by clinical and environmental *Aspergillus fumigatus* strains. Int J Med Microbiol.

[CR235] Lallouette P (1962) Extraction and purification of a growth factor. French patent no. 1,284,511 (June 18)

[CR236] Larsen TO, Smedsgaard J, Nielsen KF, Hansen ME, Frisvad JC (2005). Phenotypic taxonomy and metabolite profiling in microbial drug discovery. Nat Prod Rep.

[CR237] Lederer E (1962). Chimie biologique –sur la structure de l’aspergillomarasmine A. C R Hebd Seance Acad Sci.

[CR238] Lee M, Cho J-Y, Lee YG, Lee HJ, Lim S-I, Lee S-Y, Nam Y-D, Moon J-H (2016). Furan, phenolic, and heptelidic acid derivatives produced by *Aspergillus oryzae*. Food Sci Biotechnol.

[CR239] Lee JH, Jo EH, Hong EJ, Kim KM, Lee I (2014). Safety evaluation of filamentous fungi isolated from industrial Doenjang Koji. J Microbiol Biotechnol.

[CR240] Lee CZ, Liou GY, Yuan GF (2006). Comparison of aflR gene sequences of strains in *Aspergillus* section *Flavi*. Microbiology-SGM.

[CR241] Lee KC, Tam EWT, Lo KC, Tsang AKL, Lau CCY, To KKW, Chan JFW, Lam CW, Yuen KY, Lau SKP, Woo PCY (2015). Metabolomics analysis reveals specific novel tetrapeptide and potential anti-inflammatory metabolites in pathogenic *Aspergillus* species. Int J Mol Sci.

[CR242] Lee YH, Tominaga M, Hayashi R, Sakamoto K, Yamado O, Akito O (2006). *Aspergillus oryzae* strains with a large deletion of the aflatoxin biosynthetic homologous gene cluster differentiated by chromosomal breakage. Appl Microbiol Biotechnol.

[CR243] Leite MAF, Sarragiotto MH, Imamura PM, Marsaioli AJ (1986). Absolute configuration of drim-9(11)-en-8-ol from *Aspergillus oryzae*. J Org Chem.

[CR244] Leutou AS, Yun K, Son BW (2016). Induced production of 6,9-dibromoflavasperone, a new radical scavenging naphthopyranone in the marine-mitflat-derived fungus *Aspergillus niger*. Arch Pharm Res.

[CR245] Lewis RE, Wiederhold NP, Lionakis MS, Prince RA, Kontoyiannis DP (2005). Frequency and species distribution of gliotoxin-producing *Aspergillus* isolates recovered from patients at a tertiary-care cancer center. J Clin Microbiol.

[CR246] Li Y, Chooi Y-H, Sheng Y, Valentine JS, Tang Y (2011). Comparative characterization of fungal anthracenone and naphthacenedione biosynthetic pathways reveals an α-hydroxylation-dependent Claisen-like cyclization catalyzed by a dimanganese thioesterase. J Amer Chem Soc.

[CR247] Li D-H, Han T, Guan L-P, Bai J, Zhao N, Li Z-L, Wu X, Hua H-M (2016). New naphthopyrones from the marine-derived fungus *Aspergillus niger* 2HL-M-8 and their in vitro antiproliferative activity. Nat Prod Res.

[CR248] Li X-B, Li Y-L, Zhou J-C, Yuan H-Q, Wang X-N, Lou H-X (2015). A new diketopiperazine heterodimer from an endophytic fungus *Aspergillus niger*. J Asian Nat Prod Res.

[CR249] Li X-B, Xie F, Liu S-S, Li Y, Zhou JC, Liu YQ, Yuan H-Q, Lou H-X (2013). Naphtho-γ-pyrones from endophyte *Aspergillus niger* occurring in the liverwort *Heteroscyphus tener* (Steph.) Schiffn. Chem Biol.

[CR250] Liljegren K, Svendsen A, Frisvad JC (1988). Mycotoxins and exoenzyme production by members of *Aspergillus* section *Flavi*: an integrated taxonomic approach to their classification. Proc Jpn Assoc Mycotoxicol Suppl.

[CR251] Lind AL, Wisecaver JH, Lameiras C, Wiemann P, Palmer JM, Keller NP, Ridrogues F, Goldman GH, Rokas A (2017). Driver of genetic diversity in secondary metabolic gene clusters within a fungal species. PLOS Biol.

[CR252] Lind AL, Wisecaver JH, Smith TD, Feng X, Calvo AM, Rokas A (2015). Examining the evolution of the regulatory circuit controlling secondary metabolism and development in the fungal genus *Aspergillus*. PLoS Genet.

[CR253] Liu L, Bao L, Wang L, Ma K, Han J, Yang Y, Liu R, Ren J, Yin W, Wang W, Liu H (2018). Asperorydines A-M: Prenylated tryptophan-derived alkaloids with neurotropic effects from *Aspergillus oryzae*. J Org Chem.

[CR254] Liu D, Li X-M, Li C-S, Gao S-S, Shang Z, Proksch P, Huang C-G, Wang B-G (2011). Nigerapyrones A-H, α-pyrone derivatives from the marine mangrove-derived endophytic fungus *Aspergillus niger* MA-132. J Nat Prod.

[CR255] Liu D, Li X-M, Li C-S, Wang B-G (2013). Nigerasterols A and B, antiproliferative sterols from the mangrove-derived endophytic fungus *Aspergillus niger* MA-132. Helv Chim Acta.

[CR256] Liu XJ, Luo XY, Hu WJ, Natori S, Ueno Y (1989). *Arthrinium* spp and the etiology of deteriorated sugarcane poisoning. Mycotoxins and phycotoxins 88.

[CR257] Liu C, Tagami K, Minami A, Matsumoto T, Frisvad JC, Suzuki H, Ishikawa J, Gomi K, Oikawa H (2015). Reconstitution of biosynthetic machinery for highly elaborated indole diterpene penitrem. Angew Chem Int Ed.

[CR258] Logrieco A., Ferracane R., Haidukowsky M., Cozzi G., Visconti A., Ritieni A. (2009). Fumonisin B2production byAspergillus nigerfrom grapes and natural occurrence in must. Food Additives & Contaminants: Part A.

[CR259] Lopez Diaz TM, Flannigan B (1997). Production of patulin and cytochalasin E by *Aspergillus clavatus* during malting of barley and wheat. Int J Food Microbiol.

[CR260] Lu S, Tian J, Sun W, Meng J, Wang X, Fu X, Wang A, Lai D, Liu Y, Zhou L (2014). Bis-naphtho-γ-pyrones from fungi and their bioactivities. Molecules.

[CR261] Lu X-T, Zhang E-X, Yin S-T, Fan L-H, Hu H-B (2017). Methylseleninic acid prevents patulin-induced hepatotoxicity and nephrotoxicity via the inhibition of oxidative stress and inactivation of p53 and MAPKs. J Agric Chem.

[CR262] Lubna AS, Hamayun M, Gul H, Lee I-J, Hussain A (2018). *Aspergillus niger* CSR3 regulates plant hormones and secondary metabolites by producing gibberellins and indoleacetic acid. J Plant Interact.

[CR263] Luk KC, Kobbe B, Townsend JM (1977). Production of cyclopiazonic acid by *Aspergillus flavus* Link. Appl Environ Microbiol.

[CR264] Lv Y, Xiao J, Pan L (2014). Type III polyketide synthase is involved in the biosynthesis of protocatechuic acid in *Aspergillus niger*. Biotechnol Lett.

[CR265] Lv Y, Zhou F, Wang B, Pam L (2015). Morphological transitions under oxidative stress in response to metabolite formation in *Aspergillus niger*. Biotechnol Lett.

[CR266] Ma Y, Li T, Ma C (2016). A new pyrone derivative from an endophytic *Aspergillus tubingensis* of *Lycium ruthenicum*. Nat Prod Res.

[CR267] Ma X, Peng J, Wu G, Zhu T, Li G, Gu Q, Li D (2015). Speradines B-D, oxygenated cyclopiazonic acid alkaloids from the sponge-derived fungus *Aspergillus flavus* MXH-X104. Tetrahedron.

[CR268] MacDonald JC (1961). Biosynthesis of aspergillic acid. J Biol Chem.

[CR269] MacDonald JC (1962). Biosynthesis of hydroxyaspergillic acid. J Biol Chem.

[CR270] MacDonald JC (1973). Toxicity, analysis, and production of aspergillic acid and its analogs. Can J Biochem.

[CR271] Machida M, Asai K, Sano M, Tanaka T, Kumagai T, Terai G, Kusumoto K, Arima T, Akita O, Kashiwagi Y, Abe K, Gomi K, Horiuchi H, Kitamoto K, Kobayashi T, Takeuchi M, Denning DW, Galaghan JE, Nierman WC, Yu JJ, Archer DB, Bennett JW, Bhatnagar D, Cleveland TE, Fedorova ND, Gotoh O, Horikawa H, Hosoyama A, Ichinomaya M, Igarashi R, Iwashita K, Juvvadi PR, Kato M, Kato Y, Kin T, Kokubun A, Maede H, Maeyama N, Maruyama J, Nagasaki H, Nakajima T, Oda K, Okada K, Paulsen I, Sakamoto K, Sawano T, Takahashi M, Takase K, Terabayashi Y, Wortman JR, Yamada O, Yamagata Y, Anazawa H, Hata Y, Koide Y, Komori T, Koyama Y, Minetoki T, Suharnan S, Taanaka A, Isono K, Kuhara S, Ogasawara N, Kukuchi H (2005). Genome sequencing and analysis of *Aspergillus oryzae*. Nature.

[CR272] Machida M, Yamada O, Gomi K (2008). Genomics of *Aspergillus oryzae*: learning from the history of koji mold and exploration of its future. DNA Res.

[CR273] Manonmani HK, Sreektaniah KR (1984). Pigment production by a strain of *Aspergillus* sp. J Food Sci Technol.

[CR274] Månsson M, Klejnstrup ML, Phipps RK, Nielsen KF, Frisvad JC, Gotfredsen CH, Larsen TO (2010). Isolation and NMR characterization of fumonisin B_2_ and B_6_, a new fumonisin from *Aspergillus niger*. J Agric Food Chem.

[CR275] Manzanares-Miralles L, Sarikaya-Bayram Ö, Smith EB, Dolan SK, Bayram Ö, Jones GW, Doyle S (2016). Quantitative proteomcis revelas the mechanism and consequence of gliotoxin-mediates dysregulation of the methionine cycle in *Aspergillus niger*. J Proteomics.

[CR276] Marston RQ (1949). Production of kojic acid from *Aspergillus lutescens*. Nature.

[CR277] Martinez D, Berka RM, Henrissat B, Saloheimo M, Arvas M, Baker SE, Chapman J, Chertkov O, Coutinho PM, Cullen D, Danchin EG, Grigoriev IV, Harris P, Jackson M, Kubicek CP, Han CS, Ho I, Larrondo LF, de Leon AL, Magnuson JK, Merino S, Misra M, Nelson B, Putnam M, Robbertse B, Salamov AA, Scmoll M, Terry A, Thayer N, Westerhom-Parvinen A, Sloch CL, Yao J, Barbote R, Nelson MA, Detter C, Bruce D, Kuske CR, Xie G, Richardson P, Rokshar H, Lucas SM, Rubin EM, Dunn-Coleman N, Ward M, Brettin TS (2008). Genome sequencing and analysis of the biomass-degrading fungus *Trichoderme reesei* (syn. *Hyprocrea jecorina*). Nat Biotechnol.

[CR278] Martinez-Luis S, Cherigo L, Arnold E, Spadafora C, Gerwick WH, Cubila-Rios L (2012). Antiparasitic and anticancer constituents of the endophytic fungus *Aspergillus* sp. strain F1544. Nat Prod Commun.

[CR279] Marui J, Ohashi-Kunihiro S, Ando T, Nishimura M, Koike H, Machida M (2010). Penicillin biosynthesis in *Aspergillus oryzae* and its overproduction by genetic engineering. J Biosci Bioeng.

[CR280] Massi FP, Sartori D, Ferranti L d S, Iamanaka BT, Taniwaki MH, Vieira MKLC, Fungaro MHP (2016). Prospecting for the incidence of genes involved in ochratoxin and fumonisin biosynthesis in Brazilian strains of *Aspergillus niger* and *A. welwitschiae*. Int J Food Microbiol.

[CR281] Matsumaru Takanori, Sunazuka Toshiaki, Hirose Tomoyasu, Ishiyama Aki, Namatame Miyuki, Fukuda Takashi, Tomoda Hiroshi, Otoguro Kazuhiko, Ōmura Satoshi (2008). Synthesis and biological properties of tensyuic acids B, C, and E, and investigation of the optical purity of natural tensyuic acid B. Tetrahedron.

[CR282] Matsuo M (1997). In vivo antioxidant activity of Okara koji, a fermented okara, by *Aspergillus oryzae*. Biosci Biotech Biochem.

[CR283] Matsuura S, Yamamoto M, Kaneko Y (1972). The structure of the pteridine glycoside from *Aspergillus oryzae*. Bull Chem Soc Japan.

[CR284] Mazzaferro LS, Hüttel W, Fries A, Müller M (2015). Cytochrome P450-catalyzed region- and stereoselective phenol coupling of fungal natural products. J Am Chem Soc.

[CR285] McCorkindale NJ, Wright JLC, Brian PW, Clarke SM, Hutchinson SA (1968). Canadensolide – an antifungal metabolite of *Penicillium canadense*. Tetrahedron Lett.

[CR286] McGivan JD, Chappell JB (1970). Avenaciolide—a specific inhibitor of glutamate transport in rat liver mitochondria. Biochem J.

[CR287] McKean C, Tang L, Tang M, Billam M, Wang Z, Theodorakis CW, Kendall RJ, Wang JS (2006). Comparative acute and combinative toxicity of aflatoxin B_1_ and fumonisin B_1_ in animals and human cells. Food Chem Toxicol.

[CR288] McKee CM, MacPhillamy HB (1943). An antibiotic substance produced by submerged cultivation of *Aspergillus flavus*. Proc Soc Exptl Biol Med.

[CR289] McKee CM, Rake G, Houck CL (1944). Studies on *Aspergillus flavus*. II. The production and properties of a penicillin-like substance—flavididin. J Bacteriol.

[CR290] Mehedi MAU, Molla AH, Khondar P, Sultana S, Islam MA, Rashid MA, Chowdhury R (2010). Pseurotin A: an antibacterial secondary metabolite from *Aspergillus fumigatus*. Asian J Chem.

[CR291] Meng J, Sun W, Mao Z, Xu D, Wang X, Lu S, Lai D, Liu Y, Zhou L, Zhang G (2015). Main ustilaginoidins and their distribution in rice false smut balls. Toxins.

[CR292] Miknis GF, Williams RW (1993). Total synthesis of (+)-aspirochlorin. J Am Chem Soc.

[CR293] Minami A, Liu CW, Oikawa H (2016). Total biosynthesis of fungal indole terpenes using cell factories. Heterocycles.

[CR294] Ming L (1995). Moldy sugarcane poisoning—a case report with a brief review. J Toxicol Clin Toxicol.

[CR295] Mituzani S, Komori K, Taniguchi T, Monde K, Kuramochi K, Tsubaki K (2016). A bioinspired synthesis of (+)-rubramide, (+)-flavipucine, and (+)-isoflavipucine. Angew Chem Int Ed.

[CR296] MIYAKE Yoshiaki, ITO Chihiro, ITOIGAWA Masataka, OSAWA Toshihiko (2007). Isolation of the Antioxidant Pyranonigrin-A from Rice Mold Starters Used in the Manufacturing Process of Fermented Foods. Bioscience, Biotechnology, and Biochemistry.

[CR297] Miyake Y, Mocchizuki M, Ito C, Itoigawa M, Osawa T (2008). Antioxidative pyranonigrins in rice mold starters and their suppressive effect on the expression of blood adhesion molecules. Biosci Biotech Biochem.

[CR298] Mogensen JM, Frisvad JC, Thrane U, Nielsen KF (2010). Production of fumonisin B_2_ and B_4_ by *Aspergillus niger* on grapes and raisins. J Agric Food Chem.

[CR299] Mogensen JM, Varga J, Thrane U, Frisvad JC (2009). *Aspergillus acidus* from Puerh tea and black tea does not produce ochratoxin A and fumonisin B_2_. Int J Food Microbiol.

[CR300] Mogi M, Nakayima S, Iguchi N (1952). Fortified miso (fermented soybean paste). II. Riboflavin production by ultraviolet-induced mutants of *Aspergillus oryzae*. J. Ferment Technol.

[CR301] Mogi M, Nakayima S, Yoshida F (1951). Fortified miso (fermented soybean paste). I. Trial brewing of miso with riboflavine-producing strain of *Aspergillus oryzae*. J Ferment Technol.

[CR302] Monti F, Ripamonti F, Hawser SP, Islam K (1999). Aspirochlorine: a highly selective and potent inhibitor of fungal protein synthesis. J Antibiot.

[CR303] Morton HE, Kocholaty W, Junowicz-Kocholaty R, Kelner A (1945). Toxicity and antibiotic activity of kojic acid produced by *Aspergillus luteo-virescens*. J Bacteriol.

[CR304] Moule Y, Moreau S, Bousquet JF (1977). Relationship between the chemical structure and the biological properties of some ermophilane compounds related to PR-toxin. Chem Biol Interact.

[CR305] Munkvold GP, Weieneth L, Proctor RH, Busman M, Blandino M, Susca A, Logrieco A, Moretti A (2018). Pathogenicity of fumonisin-producing and nonproducing strains of *Aspergillus* species in section *Nigri* to maize ears and seedlings. Plant Dis.

[CR306] Murakami H (1971). Classification of the koji mould. J Gen Appl Microbiol.

[CR307] Murakami H (1979). Summary and description of species of the black aspergilli. Taxonomic studies on Japanese Industrial strains of the *Aspergillus* (Part 33). J Brew Soc Japan.

[CR308] Mushtaq S, Abbasi BH, Uzair B, Abbasi R (2018). Natural products as reservoirs of novel therapeutic agents. EXCLI J.

[CR309] Nagano N, Umemura M, Izumikawa M, Kawano J, Ishii T, Kikuchi M, Tomii K, Kumagai T, Yoshimi A, Machida M, Abe K, Shin-ya K, Asai K (2016). Class of cyclic ribosomal peptide synthetic genes in filamentous fungi. Fungal Genet Biol.

[CR310] Nair MG (1998). Fumonisin and human health. Ann Tropic Paed.

[CR311] Nakamura S (1960). Muta-aspergillic acid, a new growth inhibitant against hiochi-bacteria. Bull Agric Chem Soc Jpn.

[CR312] Nakamura S (1961). The structure of muta-aspergillic acid. Agric Biol Chem.

[CR313] Nakamura S, Shimoda C (1954). Studies on antibiotic substance oryzacidin, produced by *Aspergillus oryzae*. Part 5. Existence of β-nitropropionic acid. J Agr Chem Soc Japan.

[CR314] Nakamura S, Shiro T (1959). Studies on growth inhibition of hiochi-bacteria, specific saprophytes of sake. 4. Hydroxyaspergillic acid as a growth inhabitant against hiochi-bacteria. Bull Agric Chem Soc Jpn.

[CR315] Nakamura S, Shiro T (1959). Hydroxyaspergillic acid as growth inhabitant against hiochi-bacteria (Studies on growth inhibition of hiochi-bacteria, specific saprophytes of sake. 4). Bull Agric Chem Soc Jpn.

[CR316] Nakazawa R (1907) On the koji fungus. Aspergillus awamori. Rept Inst Gov Res Formosa 1

[CR317] Nakazawa M, Uehara T, Nomura Y (1997). Koningic acid (a potent glyceraldehyde-3-phosphate dehydrogenase inhibitor) induced fragmentation and condensation of DNA in NG108-15 cells. J Neurochem.

[CR318] Nicholson MJ, Koulman A, Monahan BJ, Pritchard BL, Payne GA, Scott B (2009). Identification of two aflatrem biosynthesis gene loci in *Aspergillus flavus* and metabolic engineering of *Penicillium paxilli* to elucidate their function. Appl Environ Microbiol.

[CR319] Nielsen KF, Gräfenham T, Zafari D, Thrane U (2005). Trichothecene production by *Trichoderma brevicompactum*. J Agric Food Chem.

[CR320] Nielsen JC, Grijseels S, Prigent S, Ji B, Dainat J, Nielsen KF, Frisvad JC, Workman M, Nielsen J (2017). Global analysis of biosynthetic gene clusters reveals vast potential of secondary metabolite production in *Penicillium* species. Nat Microbiol.

[CR321] Nielsen KF, Mogensen JM, Johansen M, Larsen TO, Frisvad JC (2009). Review of secondary metabolites and mycotoxins from the *Aspergillus niger* group. Anal Bioanal Chem.

[CR322] Nierman WC, Yu J, Fedorova-Adams ND, Losada L, Cleveland TE, Bhatnagar D, Bennett JW, Dean R, Payne GA (2015). Genome sequence of *Aspergillus flavus* NRRL 3357, a strain that causes aflatoxin contamination of food and feed. Genome Announc.

[CR323] Nishie K, Porter JK, Cole RJ, Dorner JW (1985). Neurochemical and pharmacological effects of cyclopiazonic acid, chlorpromazine and reserpine. Res Commun Psychol Psych Behav.

[CR324] Nishimura A, Okamoto S, Yoshizako F, Morishima I, Ueno T (1991). Stimulatory effect of acetate and propionate on aspergillic acid formation by *Aspergillus oryzae* A21. J Ferment Bioeng.

[CR325] Nishimura I, Shinohara Y, Oguma T, Koyama Y (2018). Survival strategy of the salt-tolerant lactic acid bacterium, *Tetragenococcus halophilus*, to counteract koji mold, *Aspergillus oryzae*, in soy sauce brewing. Biosci Biotechnol Biochem.

[CR326] Niu J, Arenthorst M, Nair PDS, Dai Z, Baker S, Frisvad JC, Nielsen KF, Punt PJ, Ram A (2016). Identification of a classical mutant in the industrial host *Aspergillus niger* by systems genetics: LaeA is required for citric acid production and regulates the formation of some secondary metabolites. G3: Gen Genom Genet.

[CR327] Nonaka N, Asai Y, Nishio M, Takahashi K, Okuda T, Tanaka S, Suita T, Ohnuki T, Komatsubara S (1997). TMC-2A, -2B and -2C, new dipeptidyl peptidase IV inhibitors produced by *Aspergillus oryzae* A374. I. Taxonomy of producing strain, fermentation, and biochemical properties. J Antibiot.

[CR328] Nozawa K, Nakajima S, Kawai K (1988). Isolation and structures of indoloterpenes, possible biosynthetic intermediates to the tremorgenic mycotoxin, paxilline, from *Emericella striata*. J Chem Soc Perkin Trans I.

[CR329] Nozawa K, Sekita S, Harada M, Udagawa S, Kawai K (1989). Isolation and structures of two new indoloterpenes related to aflavinine from a microsclerotium-producing strain of *Aspergillus flavus*. Chem Pharm Bull.

[CR330] Oda M, Saraya T, Wakayama M, Shibuya K, Ogawa Y, Ihui T, Yokoyama E, Inoue M, Shimayamada H, Fujiwara M, Ota T, Takizawa H, Goto H (2013) Calcium oxalate crystal deposition in a patient with aspergilloma due to *Aspergillus niger*. J Thorac Dis 5:E174–E178 10.3978/j.issn.2072-1439.2013.08.45 PMC3755682 23991333

[CR331] Ohmomo S, Sugita M, Abe M (1973). Isolation of cyclopiazonic acid, cyclopiazonic acid imine and bissecodehydrocyclopiazonic acid from the cultures of *Aspergillus versicolor* (Vuill.) Tiraboschi. J Agric Chem Soc Japan.

[CR332] Ohta A, Ohta M (1983). Synthesis of mutaaspergillic and dl-hydroxyaspergillic acids. Chem Pharm Bull.

[CR333] Orth R (1977). Mycotoxins of *Aspergillus oryzae* strains for use in the food industry as starters and enzyme producing strains. Ann Nutr Aliment.

[CR334] Ovenden SPB, Sberna G, Tait RM, Wildman HG, Patel R, Li B, Steffy K, Nguyen N, Meurer-Grimes BM (2004). A diketopiperazine dimer from a marine-derived isolates of *Aspergillus niger*. J Nat Prod.

[CR335] Park H-S, Jun S-C, Han K-H, Hong S-B, Yi J-H (2017). Diversity, application, and synthetic biology of industrially important *Aspergillus* fungi. Adv Appl Microbiol.

[CR336] Park SY, Oh HH, Park YL, Yu HM, Myung DS, Ch SB, Lee WS, Park D, Joo YE (2017). Malformin A1 treatment alters invasive and oncogenic phenotypes of human colorectal cancer cells through stimulation of the p38 singaling pathway. Int J Oncol.

[CR337] Parrish FW, Wiley BJ, Simmons EG, Lang L (1966). Production of aflatoxins and kojic acid by species of *Aspergillus* and *Penicillium*. Appl Microbiol.

[CR338] Patron NJ, Waller RF, Cozijnsen AJ, Straney DC, Gardiner DM, Nierman WC, Howlett BJ (2007). Origin and distribution of epipolythiodioxopiperazine (ETP) gene clusters in filamentous ascomycetes. BMC Evol Biol.

[CR339] Pattenden G (1969). Synthesis of asperenone, a new pigment from *Aspergillus niger* and *Aspergillus awamori*. Tetrahedron Lett.

[CR340] Pattenden G (1970). Synthesis of asperenone [all-trans(E)-8-methyl-13-phenyltrideca-4,6,8,10,12-pentaen-3-one], a pigment of *Aspergillus* species of fungi. J Chem Soc (C).

[CR341] Payne GA, Nierman WC, Wortman JR, Pritchard BL, Brown D, Dean RA, Bhatnagar D, Cleveland TE, Machida M, Yu Y (2006). Whole genome comparison of *Aspergillus flavus* and *A. oryzae*. Med Mycol.

[CR342] Pel HJ, van de Winde JH, Archer DB, Dyer PS, Hofmann G, Schaap PJ, Turner G, de Vries RP, Albang R, Alberman K, Andersen MR, Bendtsen JD, Benen JA, van den Berg M, Breetstraat S, Caddick MS, Contreras R, Cornell M, Coutinho PM, Dancnin EG, Debets AJ, Dekker P, van Dijck PW, van Dijk A, Dijkhuizen L, Driessen AJ, d’Enfert C, Geysens S, Goosen C, Groot GS, de Groot PW, Guillemette T, Henrissat B, Herweijer M, van den Homberg JP, van den Hondel CA, van der Heijden RT, van der Kaaij RM, Klis FM, Kools HJ, Kubicek CP, van Kuyk PA, Lauber J, Lu X, van der Marel MJ, Meulenberg R, Menke H, Mortimer MA, Nielsen J, Oliver SG, Olsthoorn M, Pal K, van Peij NN, Ram AF, Rinas U, Troubos JA, Sagt CM, Schmoll M, Sun J, Ussery D, Varga J, Vervecken W, van de Vondervoort PJ, Wedler H, Wosten HA, Zeng AP, van Oorwen AJ, Visser J, Stam H (2007). Genome sequencing and analysis of the versatile cell factory *Aspergillus niger* CBS 513.88. Nat Biotechnol.

[CR343] Perrone G, Stea G, Epifani F, Varga J, Frisvad JC, Samson RA (2011). *Aspergillus niger* contains the cryptic phylogenetic species *A. awamori*. Fungal Biol.

[CR344] Perry MJ, Makins JF, Adlard MW, Holt G (1984). Aspergillic acids produced by mixed cultures of *Aspergillus flavus* and *Aspergillus nidulans*. J Gen Microbiol.

[CR345] Petersen LM, Holm DK, Knudsen PB, Nielsen KF, Gotfredsen CH, Mortensen UH, Larsen TO (2015). Characterization of four new yanuthones from *Aspergillus niger*. J Antibiot.

[CR346] Pfefferle W, Anka H, Bross M, Steffan B, Vianden R, Steglich W (1990). Asperfuran, a novel antifungal metabolite from *Aspergillus oryzae*. J Antibiot.

[CR347] Pinheiro EAA, Carvalho JM, dos Santos DCP, Feitosa AD, Marinho PSB, Guilhon GMSP, de Souza ADL, da Silva FMA, Marinho AMD (2013). Antibacterial activity of alkaloids produced by endophytic fungus *Aspergillus* sp EJC08 isolated from medical plant *Bauhinia guianensis*. Nat Prod Res.

[CR348] Pitt JI, Hocking AD, Glenn DR (1983). An improved medium for the detection of *Aspergillus flavus* and *Aspergillus parasiticus*. J Appl Bacteriol.

[CR349] Plattner PA, Clauson-Kaas N (1945). Űber ein Welke erzeugendes Stoffwechselproducte von *Fusarium lycopersici* Sacc. Helv Chim Acta.

[CR350] Pócsfalvi G, Ritieni A, Ferranti P, Randazzo G, Vékey K, Malorni A (1997). Microheterogeneity characterization of a paracelsin mixture from *Trichoderma reesei* using high-energy collision-induced dissociation tandem mass spectrometry. Rap Commun Mass Spectrom.

[CR351] Pontovich VE (1943). *Aspergillus flavus* as a source of flavin. Biokhimiya.

[CR352] Powell Amy J, Conant Gavin C, Brown Douglas E, Carbone Ignazio, Dean Ralph A (2008). Altered patterns of gene duplication and differential gene gain and loss in fungal pathogens. BMC Genomics.

[CR353] Poulsen L, Andersen MR, Lantz AE, Thykaer J (2012). Identification of a transcription factor controlling pH-dependent organic response in *Aspergillus niger*. PLoS ONE.

[CR354] Price MN, Dehal PS, Arkin AP (2009). FastTree: computing large minimum evolution trees with profiles instead of a distance matrix. Mol Biol Evol.

[CR355] Priegnitz B-E, Brandt U, Pahirulzaman AK, Dickschat JS, Fleissner A (2015). The AngFus3 mitogen-activated protein kinase controls hyphal differentiation and secondary metabolism in *Aspergillus niger*. Euk Cell.

[CR356] Przybylski M, Dietrich I, Manz I, Brückner H (1984). Elucidation of structure and microheterogeneity of the polypeptide antibiotics paracelsin and trichotoxin A-50 by fast atom bombardment mass spectrometry in combination with selective in situ hydrolysis. Biomed Mass Spectrom.

[CR357] Purchase IFH (1971). The acute toxicity of the mycotoxin cyclopiazonic acid to rats. Toxicol Appl Pharmacol.

[CR358] Purchase IFH, Theron JJ (1968). The acute toxicity of ochratoxin A to rats. Food Cosmet Toxicol.

[CR359] Qiao M-F, Ji N-Y, Liu X-H, Li F, Xue Q-Z (2010). Asporyergosterol, a new steroid from an algicolous isolate of *Aspergillus oryzae*. Nat Prod Commun.

[CR360] Qiao M-F, Ji N-Y, Liu X-H, Li K, Zhu Q-M (2010). Indoloterpenes from an algicolous isolate of *Aspergillus oryzae*. Bioorg Med Chem Lett.

[CR361] Qiao M-F, Ji N-Y, Miao F-P, Yin X-L (2011). Steroids and an oxylipin from an algicolous isolate of *Aspergillus flavus*. Magn Res Chem.

[CR362] Rabache M, Neuman J, Lavollay J (1974). Phenylpolyenes d’*Aspergillus niger*: structure et proprietes de l’asperrubrol. Phytochem.

[CR363] Rahssaparpoor P (2014). An investigation on the patterns of deoxunivalenol (DON) mean values in *Aspergillus* isolates, based on subgenus and species correlations. Ind J Fund Appl Life Sci.

[CR364] Raistrick H, Clark AB (1919). On the mechanism of oxalic acid formation by *Aspergillus niger*. Biochem J.

[CR365] Ramakrishnan CV, Desai PJ (1956). Effect of addition of iron, cobalt, and ascorbic acid to the medium on the synthesis of ascorbic acid in molds. Curr Sci (India).

[CR366] Ramakrishnan CV, Sathe V (1956). Effect of vitamin K_3_ on inducing its biosynthesis in moulds. Sci Cult (Calcutta).

[CR367] Rand TG, Chang CT, McMullin DR, Miller JD (2017). Inflammation-associated gene expression in RAW 264.7 macrophages induced by toxins from fungi common on damp building materials. Toxicol in Vitro.

[CR368] Rank C, Klejnstrup ML, Petersen LM, Kildegaard S, Frisvad JC, Gotfredsen CH, Larsen TO (2012). Comparative chemistry of *Aspergillus oryzae* (RIB40) and *A. flavus* (NRRL 3357). Metabolites.

[CR369] Rao KCS, Divakar S, Babu KN, Rao AGA, Karanth NG, Sattur AP (2002). Nigerloxin, a novel inhibitor of aldose reductase and lipoxygenase with free radical scavenging activity from *Aspergillus niger* CFR-W-105. J Antibiot.

[CR370] Rao KCS, Divakar S, Rao AGA, Karanth NG, Suneetham WJ, Krishnakantha TP, Sattur AP (2002). Asperenone: an inhibitor of 15-lipoxygenase and of human platelet aggregation from *Aspergillus niger*. Biotechnol Lett.

[CR371] Rao K.C. Sekhar, Karanth N.G., Sattur A.P. (2005). Production of nigerloxin, an enzyme inhibitor and a free radical scavenger, by Aspergillus niger using solid state fermentation. Process Biochemistry.

[CR372] Raper KB (1946). The development of improved penicillin-producing molds. Ann NY Acad Sci.

[CR373] Raper KB, Fennell DI (1965). The genus *Aspergillus*.

[CR374] Ray AC, Eakin RE (1975). Studies on the biosynthesis of aspergillin by *Aspergillus niger*. Appl Microbiol.

[CR375] Ren R, Chen C-J, Hu S-S, Ge H-M, Zhu W-Y, Tan R-X, Jiao R-H (2015). Drimane sesquiterpenoids from the *Aspergillus oryzae* QXPC-4. Chem Biodiv.

[CR376] Robert M, Barbier M, Lederer E, Roux L, Biemann K, Vetter W (1962). Two new natural phytotoxins: Aspergillomarasmines A and B and their identity to lycomarasmine and its derivatives. Bull Soc Chim Fr.

[CR377] Rodricks JV, Lustig E, Campbell AD, Stoloff L (1968). Aspertoxin, a hydroxyl derivative of O-methylsterigmatocystin from aflatoxin producintg cultures of *Aspergillus flavus*. Tetrahedron Lett.

[CR378] Rodrigues BSF, Sahm BDB, Jimenez PC, Pinto FCL, Mafezoli J, Mattos MC, Rodrigues-Filho E, Pfenning LH, Abreau LM, Costa-Lotufo LV, Oliveira MCF (2015). Bioprospection of cytotoxic compounds in fungal strains recovered from sediments of the Brazilian coast. Chem Biodiv.

[CR379] Rodriguez RLM, Konstantinidis KT (2014). Bypassing cultivation to identify bacterial species. Microbe.

[CR380] Rodríguez A, Rodríguez M, Luque MI, Martín A, Córdoba JJ (2012). Real-tine PCR assays for detection and quantification of aflatoxin-producing molds in foods. Food Microbiol.

[CR381] Rokas A (2009). The effect of domestication on the fungal proteome. Trends Genet.

[CR382] Rokas A, Payne G, Fedorova ND, Baker SE, Machida M, Yu J, Georgianna DR, Dean RA, Bhatnagar D, Cleveland TE, Wortman JR, Maiti R, Joardar V, Denning DW, Nierman WC (2007). What can comparative genomics tell us about species concepts in the genus *Aspergillus*?. Stud Mycol.

[CR383] Saito K (1907). Űber die Säurebildung bei *Aspergillus oryzae*. Botan Mag (Tokyo).

[CR384] Saito M, Tsuruta O (1993). A new variety of *Aspergillus flavus* from tropical soil in Thailand and its aflatoxin productivity. Proc Jpn Assoc Mycotoxicol.

[CR385] Sakaguchi K, Iizuka H, Yamazaki S (1951). A study on the black aspergilli. J Agric Chem Soc Japan.

[CR386] Sakaguchi K, Takahashi H, Morino H (1953). Production of α-ketoglutaric acid by molds. J Agr Chem Soc Japan.

[CR387] Sakai H (1953). On vitamin B_12_ production by fermentation. Part. 2. Production test of B_12_ by various microorganisms. J Agr Chem Soc Japan.

[CR388] Sakai K, Kinioshita H, Shimuzu T, Nihira T (2008). Construction of a citrinin gene cluster expression system in heterologous *Aspergillus oryzae*. J Biosci Bioeng.

[CR389] Sakata K, Kuwatsuka T, Sakurai A, Takahashi N, Tamura G (1983). Isolation of aspirochlorine (= antibiotic A 30641) as a true antimicrobial constituent of the antibiotic oryzachlorin, from *Aspergillus oryzae*. Agric Biol Chem.

[CR390] Sakata K, Maruyama M, Uzawa J, Sakurai A, Lu HSM, Clardy J (1987). Structural revision of aspirochlorine (= antibiotic A 30641), a novel epidithiopiperazine-2,5-dione produced by *Aspergillus* spp. Tetrahedron Lett.

[CR391] Sakata K, Masago H, Sakurai A, Takahashi N (1982). Isolation of aspirochlorine (= antibiotic A 30641) possessing a novel dithiodiketopiperazine structure from *Aspergillus flavus*. Tetrahedron Lett.

[CR392] Sakata K, Masahito M, Kuwatsuka T, Uzawa J, Sakurai A, Lu HSM, Clardy J (1987b) Structure of aspirochlorine, a novel epidithiopiperazine-2,5-dione, and related compounds produced by *Aspergillus* spp. 29th Symp Chem Nat Prod 29:685–691

[CR393] Saldan NC, Almeida RTR, Avíncola A, Porot C, Galuch MB, Magon TFS, Pilau EJ, Svidzinski TIE, Oliveira CC (2018). Development of an analytical method for identification of *Aspergillus flavus* based on chemical markers using HPLC-MS. Food Chem.

[CR394] Samson RA, Hong S-B, Peterson SW, Frisvad JC, Varga J (2007). Polyphasic taxonomy of *Aspergillus* section *Fumigati* and its teleomorph *Neosartorya*. Stud Mycol.

[CR395] Samson RA, Visagie CM, Houbraken J, Hong S-B, Hubka V, Klaassen CHW, Perrone G, Seifert KA, Susca A, Tanney JB, Varga J, Kocsubé S, Szigeti G, Yaguchi T, Frisvad JC (2014). Phylogeny, identification and nomenclature of the genus *Aspergillus*. Stud Mycol.

[CR396] Samuels GJ, Ismaiel A, Mulaw TB, Szakacs G, Druzhinina IS, Kubicek CP, Jaklitsch WM (2012). The *Longibrachiatum* clade of *Trichoderma*: a revision with new species. Fungal Divers.

[CR397] Samuels GJ, Petrini O, Kuhls K, Lieckfeldt E, Kubicek CP (1998). The *Hypocrea schweinitzii* complex and *Trichoderma* sect. *Longibrachiatum*. Stud Mycol.

[CR398] Sano Y, Ishikawa TY, Muramatsu S, Uzuka Y, Kokubo S, Omata S, Kitamura T, Matsugo S (2007). Study on the Koji mold producing substrate – possible application as a food supplement. Food Sci Technol.

[CR399] Sasaki M, Asoa Y, Yokotsuka T (1968) Studies of the compounds produced by molds part V. Isolation of non-fluorescent pyrazine compounds (2). J Agric Chem Soc Jpn 42:351–355 (in Japanese)

[CR400] Sato N, Horiuchi T, Hamano M, Sekine H, Chiba S, Yamamoto H, Yoshioka T, Kimura I, Satake M, Ida Y (1996). Kojistatin A, a new cysteine protease inhibitor produced by *Aspergillus oryzae*. Biosci Biotech Biochem.

[CR401] SCCS (Scientific Committee on Consumer Safety) (2012) Opinion on kojic acid, 26–27 June 2012.

[CR402] Schlingmann G, Taniguchi T, He H, Bigelis R, Yang HY, Koehn FE, Carter GT, Berova N (2007). Reassessing the structure of pyranonigrin. J Nat Prod.

[CR403] Schmoll M, Dattenböck C, Carreras-Villaseñor N, Mendoza-Mendoza A, Tisch D, Alemán MI, Baker SE, Brown C, Cervantes-Badillo MG, Cetz-Chel J, Cristobal-Mondragon GR, Delaye H, Esquivel-Naranjo EU, Frischmann A, Gallardo-Negrete JD, Garca-Esquivel M, Gomez-Rodriguez E, Greenwood DR, Hernandez-Onata M, Kruszewska JS, Lawrey R, Mora-Montes HM, Munoz-Centeno T, Nieto-Jacobo MF, Lopez GN, Olmedo-Nonfil V, Osoroa-Conceosion M, Pilsyk S, Pomraning KR, Rodriguez-Iglesias A, Rosales-Saavedra MT, Sanchez-Arregyúin JA, Sedl-Seiboth V, Stewart A, Iresti-Rivera EE, Wang CL, Wang TF, Zeilinger S, Casas-Flores S, Herrera-Estrella A (2016). The genomes of three uneven siblings: Footprints of the lifestyles of three *Trichoderma* species. Microbiol Mol Biol Rev..

[CR404] Schroeder HW, Kelton WH (1975). Production of sterigmatocystin by some species of the genus *Aspergillus* and its toxicity to chicken embryos. Appl Microbiol.

[CR405] Schuster E, Dunn-Coleman N, Frisvad JC, van Dijck PWM (2002). On the safety of *Aspergillus niger*—a review. Appl Microbiol Biotechnol.

[CR406] Seshime Y, Juvvadi PR, Fujii I, Kitamoto K (2005). Discovery of a novel superfamily of type III polyketide synthases in *Aspergillus oryzae*. Biochem Biophys Res Commun.

[CR407] Seshime Y, Juvvadi PR, Kitamoto K, Ebizuka Y, Fujii I (2010). Identification of csypyrone B1 as the novel product from *Aspergillus oryzae* type III polyketide synthase CsyB. Biomed. Med Chem.

[CR408] Seshime Y, Juvvadi PR, Kitamoto K, Ebizuka Y, Nonaka T, Fujii I (2010). *Aspergillus oryzae* type III polyketide synthase csyA is involved in the biosynthesis of 3,5-dihydroxy-benzoic acid. Biomed. Med Chem Lett.

[CR409] Shaaban M, El-Metwally MM, Nasr H (2014). A new diketopiperazine alkaloid from *Aspergillus oryzae*. Nat Prod Res.

[CR410] Shen L, Ye Y-H, Wang X-T, Zhu H-L, Xu C, Song YC, Li H, Tan R-X (2006). Structure and total synthesis of aspernigerin: a novel cytotoxic endophyte metabolite. Chem Eur J.

[CR411] Shi YS, Zhang Y, Chen XZ, Zhang N, Liu YB (2015). Metabolites produced by the endophytic fungus *Aspergillus fumigatus* from the stem of *Erythrophloeum fordii* Oliv. Molecules.

[CR412] Shigehisa H, Kikuchi H, Suzuki T, Hiroya K (2015). The revised structure of trichodermatide A. Eur J Org Chem.

[CR413] Shimizu Y, Ogata H, Goto S (2017). Type III polyketide synthases: functional classification and phylogenomics. ChemBioChem.

[CR414] Shimoda C (1951). An antibacterial substance, oryzacidin, against sake-putryfying bacteria, produced by *Aspergillus oryzae*. J Agric Chem Soc Japan.

[CR415] Shinohara Y, Kawatani M, Futamura Y, Osada H, Koyama Y (2016). An overproduction of astellolides induced by genetic disruption of chromatin-remodeling factors in *Aspergillus oryzae*. J Antibiot.

[CR416] Shiomi K, Hatae K, Yamaguchi Y, Masuma R, Tomoda H, Kobayashi S, Omura S (2002). New antibiotics miyakamides produced by a fungus. J Antibiot.

[CR417] Shishido K, Omodani T, Shibuya M (1991). Novel and enantioselective total synthesis of drimane type sesquiterpenes. J Chem Soc Perkin Trans.

[CR418] Shu Y-Z, Cutrone JQ, Klohr SE, Huang S (1995). BMS-192548, a tetracyclic binding inhibitor of neuropeptide Y receptors, from *Aspergillus niger* WB2346. II Physicochemical properties and structural characterization. J Antibiot.

[CR419] Skóra J, Sulyok M, Nowak A, Otlewska A, Gutarowska B (2017). Toxinogenicity and cytotoxicity of *Alternaria*, *Aspergillus* and *Penicillium* moulds isolated from working environments. Int J Environ Sci Technol.

[CR420] Son SY, Lee S, Singh D, Lee N-R, Lee D-Y, Lee CH (2018) Comprehensive secondary metabolite profiling toward delineating the solid and submerged-state fermentation of *Aspergillus oryzae* KCCM 12698. Front Microbiol 9 Art 1076 10.3389/fmicb.2018.01076 PMC5981208 29887844

[CR421] Sørensen LM, Lametsch R, Andersen MR, Nielsen PV, Frisvad JC (2009). Proteome analysis of *Aspergillus niger*: lactate added in starch-containing medium can increase production of the mycotoxin fumonisin B_2_ by modifying acetyl-CoA metabolism. BMC Microbiol.

[CR422] Springer JP, Büchi G, Kobbe B, Demain AL, Clardy J (1977). The structure of ditryptophenaline—a new metabolite of *Aspergillus flavus*. Tetrahedron Lett.

[CR423] Srinisavan KS, Ramakrishnan CV (1952). Synthesis of thiamine by molds. Biochem Biophys Acta.

[CR424] Stockmann-Juvala H, Savolainen K (2008). A review of the toxic effects and mechanisms of action of fumonisin B_1_. Hum Exp Toxicol.

[CR425] Sugawara F, Kim K-W, Uzawa J, Yoshida S, Takahashi N, Curtis RW (1990). Structure of malformin A_2_, reinvestigation of phytotoxic metabolites produced by *Aspergillus niger*. Tetrahedron Lett.

[CR426] Sugimoto T, Murata S, Matsuura S, Pfleiderer W (1986). Synthesis of asperopterin B and some analogues. Tetrahedron Lett.

[CR427] Sugiyama M, Masaki M, Ohta M (1967). Synthesis of 1-hydroxy-3-isobutyl-6-(1-hydroxy-1-methylethyl)-2-pyrazinone and the structure of muta-aspergillic acid. Tetrahedron Lett.

[CR428] Sun Y, Laian T, Huangn J, Ma H-Y, Lv A-L, Yasukawa K, Pei Y-H (2008). Trichodermatides A-D, novel polyketides from the marine-derived fungus *Trichoderma reesei*. Org Lett.

[CR429] Sun K, Li Y, Guo L, Wang Y, Liu P, Zhu W (2014). Indole diterpenoids and isocoumarin from the fungus *Aspergillus flavus*, isolated from the prawn, *Penaeus vannamei*. Mar Drugs.

[CR430] Suresha BS, Srinivasan K (2013). Antioxidant properties of fungal metabolite nigerloxin in vitro. Appl Biochem Microbiol.

[CR431] Susca A, Proctor RH, Butchko RAE, Haidukowski M, Stea G, Logrieco A, Moretti A (2014). Variation in the fumonisin biosynthetic gene cluster in fumonisin-producing and nonproducing black aspergilli. Fungal Genet Biol.

[CR432] Taevernier L, Wynendaele E, de Vreese L, Burvenich C, de Spiegeleer B (2016). The mycotoxin definition reconsidered towards fungal cyclic depsipeptides. J Environ Sci Health, Part C.

[CR433] Tagami Koichi, Minami Atsushi, Fujii Ryuya, Liu Chengwei, Tanaka Mizuki, Gomi Katsuya, Dairi Tohru, Oikawa Hideaki (2014). Rapid Reconstitution of Biosynthetic Machinery for Fungal Metabolites inAspergillus oryzae: Total Biosynthesis of Aflatrem. ChemBioChem.

[CR434] Takagi M, Motohashi K, Hwang J-H, Nagai A, Shin-ya K (2010). New tensidols, JBIR-86 and JBIR-87, isolated from *Aspergillus* sp. fJ80. J Antibiot.

[CR435] Takahashi T, Jin FJ, Sunagawa M, Machida M, Koyama Y (2008). Generation of large chromosomal deletions in koji molds *Aspergillus oryzae* and *Aspergillus sojae* via loop-out recombination. Appl Environ Microbiol.

[CR436] Takahashi T, Ogawa M, Koyama Y (2012). Analysis of the functions of recombination-related genes in the generation of large chromosomal deletions by loop-out recombination in *Aspergillus oryzae*. Euk Cell.

[CR437] Talontsi FM, Tatong MD, Dittrich B, Douanla-Meli C, Laatsch H (2013). Structures and absolute configuration of three α-pyrones from an endophytic fungus *Aspergillus niger*. Tetrahedron.

[CR438] Tamiya H (1927). Studien über die Stoffwechselprodukte von *Aspergillus oryzae*. I. Metabolic physiology of *Aspergillus oryzae*. I. Acta Phytochim Jpn.

[CR439] Tamogami S, Katayama M, Marumo S, Isobe M (1996). Synthesis of the 5-demethyl-6-deoxy analogue of sporogen AO1, a sporogenic substance of *Aspergillus oryzae*. Biosci Biotech Biochem.

[CR440] Tan Q-W, Gao F-L, Wang F-R, Chen Q-J (2015). Anti-TMV activity of malformin A_1_, a cyclic penta-peptide produced by an endophytic fungus *Aspergillus tubingensis* FJBJ11. Int J Mol Sci.

[CR441] Tanabe M, Hamasaki T, Suzuki Y, Johnson LF (1973). Biosynthetic studies with carbon-13: Fourier nuclear magnetic resonance spectra of the metabolite avenaciolide. J Chem Soc Chem Commun.

[CR442] Tanaka K, Goto T, Manabe M, Matsuura S (2002). Traditional japanese fermented foods free from mycotoxin contamination. Jpn Agric Res Quart.

[CR443] Tanaka T, Hasegawa A, Aoki N, Yamamoto S, Udagawa S, Sekita S, Harada M, Nozawa K, Kawai K (1989). Production of aflatrem and its related indoloterpenes by microsclerotium-producing strains of *Aspergillus flavus*. Proc Jpn Assoc Mycotoxicol.

[CR444] Tanaka S, Wada K, Katayama M, Marumo S (1984). Isolation of sporogen-AO1, a sporogenic substance from *Aspergillus oryzae*. Agric Biol Chem.

[CR445] Tanaka S, Wada K, Marumo S, Hattori H (1984). Structure of sporogen-AO1, a sporogenic substance. Tetrahedron Lett.

[CR446] Tang M-C, Lin H-C, Li D, Zou Y, Li J, Xu W, Cacho RA, Hillenmeyer ME, Garg NK, Tang Y (2015). Discovery of unclustered fungal indole diterpene biosynthetic pathways through combinatorial pathway reassembly in engineered yeast. J Am Chem Soc.

[CR447] Tao L, Chung SH (2014). Non-aflatoxigenicity of commercial *Aspergillus oryzae* strains due to genetic defects compared to aflatoxigenic strains. J Microbiol Biotechnol.

[CR448] TePaske MR, Gloer JB, Wicklow DT, Dowd PF (1990). Aflavazole: a new antiinsectan carbazole metabolite from the sclerotia of *Aspergillus flavus*. J Org Chem.

[CR449] TePaske MR, Gloer JB, Wicklow DT, Dowd PF (1991). Leporin A—an antiinsectan N-alkoxypyridone from the sclerotia of *Aspergillus leporis*. Tetrahedron Lett.

[CR450] TePaske MR, Gloer JB, Wicklow DT, Dowd PF (1992). Aflavarin and beta-aflatrem—new anti-insectan metabolites from the sclerotia of *Aspergillus flavus*. J Nat Prod.

[CR451] Terabayashi Y, Sano M, Yamane N, Maruui J, Tamano K, Sagara J, Dohmoto M, Oda K, Ohshima E, Tachibana K, Higa Y, Ohashi S, Koike H, Machida M (2010). Identification and characterization of genes responsible for biosynthesis of kojic acid, an industrially important compound from *Aspergillus oryzae*. Fungal Genet Biol.

[CR452] Thom C, Church MB (1921). *Aspergillus flavus*, *A. oryzae*, and associated species. Amer J Bot.

[CR453] Tokuoda M, Seshime Y, Fujii I, Kitamoto K, Takahashi T, Koyama Y (2008). Identification of a novel polyketide synthase-nonribosomal peptide synthase (PKS-NRPS) gene required for the biosynthesis of cyclopiazonic acid in *Aspergillus oryzae*. Fungal Genet Biol.

[CR454] Tokuoka M, Kukuchi T, Shinohara Y, Koyama A, Iio S, Kubota T, Kabayashi J, Kayama A, Shindo H, Sato K (2015). Cyclopiazonic acid biosynthesis gene cluster cpaM is required for speradine A biosynthesis. Biosci Biotechnol Biochem.

[CR455] Tominaga M, Lee Y-H, Hayashi R, Suzuki Y, Yamada O, Sakamoto K, Gotoh K, Akita O (2006). Molecular analysis of an inactive aflatoxin biosynthesis gene cluster in *Aspergillus oryzae* RIB strains. Appl Environ Microbiol.

[CR456] Torres J, Guarro J, Suarez G, Sune N, Ramírez C (1980). Morphological changes in strains of *Aspergillus flavus* Link ex Fries and *Aspergillus parasiticus* Speare related with aflatoxin production. Mycopathologia.

[CR457] Tsuda M, Mugishima T, Komatus K, Sone T, Tanaka M, Mikami Y, Shiro M, Hirai M, Ohizumi Y, Kobayashi J (2003). Speradine A, a new pentacyclic oxindole alkaloid from a marine-derived fungus *Aspergillus tamarii*. Tetrahedron.

[CR458] Tsukui T, Nagano N, Umemura M, Kumagai T, Terai G, Machida M, Asai K (2015). Ustiloxins, fungal cyclic peptides, are ribosomally synthesized in *Ustilaginoidea virens*. Bioinformatics.

[CR459] Turner WB (1971). Fungal metabolites.

[CR460] Turner WB, Aldridge DC (1983). Fungal metabolites II.

[CR461] Uchoa Paula Karina S., Pimenta Antonia T. A., Braz-Filho Raimundo, de Oliveira Maria da Conceição F., Saraiva Natália N., Rodrigues Barbara S. F., Pfenning Ludwig H., Abreu Lucas M., Wilke Diego V., Florêncio Katharine G. D., Lima Mary Anne S. (2017). New cytotoxic furan from the marine sediment-derived fungi Aspergillus niger. Natural Product Research.

[CR462] Ueno T, Nishimura A, Yoshizako F (1977). Isolation and identification of a new analogue of aspergillic acid derived from valine and isoleucine. Agric Biol Chem.

[CR463] Ui H, Shiomi K, Yamaguchi Y, Masuma R, Nagamitsu T, Takano D, Sunazuka T, Namikoshi M, Omura S (2001). Nafuredin, a novel inhibitor of NADH-fumarate reductase, produced by *Aspergillus niger* FT-0554. J Antibiot.

[CR464] Uka V, Moore GG, Arroyo-Manzanares N, Nebija D, De Saeger S, Mavungu JDD (2017). Unravelling the diversity of the cyclopiazonic acid family of mycotoxins in *Aspergillus flavus* by UHPLC triple-TOF HRMS. Toxins.

[CR465] Umemura M, Koike H, Nagano N, Ishii T, Kawano J, Yamane N, Kozone I, Horimoto K, Shin-ya K, Asai K, Yu J, Bennett JW, Machida M (2013). MIDDAS-M: Motif-independent de novo deetction of secondary metabolite gene clusters through the integration of genome sequencing and trinscriptome data. PLoS ONE.

[CR466] Umemura M, Koike H, Yamane N, Koyama Y, Satou Y, Kikuzato I, Teruya M, Tsukahara M, Imada Y, Wachi Y, Miwa Y, Yano S, Tamano K, Kawarabayasi Y, Fujimoro KE, Machida M, Hirano T (2012). Comparative genome analysis between *Aspergillus oryzae* strains reveals close relationship between sites of mutation localization and regions of highly divergent genes among *Aspergillus* species. DNA Res.

[CR467] Umemura M, Koyama Y, Takeda I, Hagiwara H, Ikegami T, Koike H, Machida M (2013). Fine de novo sequencing of a fungal genome using only SOLiD short read data: verfication on *Aspergillus oryzae* RIB40. PLoS ONE.

[CR468] Umemura M, Nagano N, Koike H, Kawano J, Ishii T, Miyamura Y, Kikuchi M, Tamano K, Yu J, Shin-ya K, Machida M (2014). Characterization of the biosynthetic gene cluster for the ribosomally synthesized cyclic peptide ustiloxin B in *Aspergillus flavus*. Fungal Genet Biol.

[CR469] Umezawa H, Tobe H, Shibamoto N, Nakamura F, Nakamura K, Matzusaki M, Takeuchi T (1975). Isolation of isoflavones inhibiting DOPA decarboxylase from fungi and *Streptomyces*. J Antibiot.

[CR470] Valdes JJ, Cameron JE, Cole RJ (1985). Aflatrem: a tremorgenic mycotoxin with acute neurotoxic effects. Environ Health Perspec.

[CR471] Van der Merwe KJ, Fourie L, Scott de B (1963). On the structure of the aflatoxins. Chem Ind.

[CR472] Varga J, Due M, Frisvad JC, Samson RA (2007). Taxonomic revision of *Aspergillus* section *Clavati* based on molecular, morphological and physiological data. Stud Mycol.

[CR473] Varga J, Frisvad JC, Kocsubé S, Brankovics B, Tóth B, Szigeti G, Samson RA (2011). New and revisited species in *Aspergillus* section *Nigri*. Stud Mycol.

[CR474] Varga J, Frisvad JC, Samson RA (2009). A reappraisal of fungi producing aflatoxin. World Mycotoxin J.

[CR475] Varga J, Frisvad JC, Samson RA (2011). Two new aflatoxin producing species and an overview of *Aspergillus* section *Flavi*. Stud Mycol.

[CR476] Varoglu M, Corbett TH, Valeriote FA, Crews P (1997). Asperazine, a selective cytotoxic alkaloid from a sponge-derived culture of *Aspergillus niger*. J Org Chem.

[CR477] Varoglu M, Crews P (2000). Biosynthetically diverse compounds from a saltwater culture of sponge-derived *Aspergillus niger*. J Nat Prod.

[CR478] Vasantha KY, Singh RP, Sattur AP (2018). A preliminary pharmacokinetic and toxicity study of nigerloxin. Ind J Biochem Biophys.

[CR479] Vernot EH, MacEwen CC, Kinkead ER (1977). Acute toxicity and skin corrosion data for some organic and inorganic compounds and aqueous solutions. Toxicol Appl Pharmacol.

[CR480] Vesth TC, Nybo JL, Theobald S, Frisvad JC, Larsen TO, Nielsen KF, Hoof JB, Brandl J, Salamov A, Ryley R, Gladden JM, Phatale P, Nielsen MT, Lyhne EK, Kogle ME, Strasser K, McDonald E, Berrey K, Clun A, Chen C, Nolan M, Sandor L, Kuo A, Lipzen A, Hainaut M, Drula E, Tsang A, Magnuson JK, Henrissat B, Wiebenga A, Simmons BA, Mäkelä MR, de Vries RP, Grigoriev IV, Mortensen UH, Baker SE, Andersen MR (2018) Section-level genome sequencing of *Aspergillus* section *Nigri* to investigate inter- and intra-species variation. Nature Genetics in press 10.1038/s41588-018-0246-1 30349117

[CR481] Vidal-Garcia M, Redrado S, Domingo MP, Marquina P, Colmemarejo C, Meis JF, Rezusta A, Pardo J, Galvez EM (2018). Production of the invasive aspergillosis biomarker bis(methylthio)gliotoxin within the genus *Aspergillus*: in vitro and in vivo metabolite quantification and genomic analysis. Front Microbiol.

[CR482] Voss KA, Riley RT (2013). Fumonisin toxicity and mechanism action: overview and current perspectives. Food Saf.

[CR483] Wada K, Tanaka S, Marumo S (1983). Structures of two new sesquiterpenes from *Aspergillus oryzae*. Agric Biol Chem.

[CR484] Waiss AC, Wiley M, Black DR, Lundin RE (1968). 3-Hydroxy-6,7-dimethoxydifuroxanthone—a new metabolite from *Aspergillus flavus*. Tetrahedron Lett.

[CR485] Waksman SA, Bugie E (1943). Strain specificity and production of antibiotic substances. II. *Aspergillus flavus-oryzae* group. Proc Nat Acad Sci U S A.

[CR486] Wang D, Bao Y-R, Yang X-X, Meng X-S, Chen G (2015). A new alkaloid from *Penicillium dipodomyicola*. Chem Nat Comp.

[CR487] Wang Yan, Wang Liuqing, Liu Fei, Wang Qi, Selvaraj Jonathan, Xing Fuguo, Zhao Yueju, Liu Yang (2016). Ochratoxin A Producing Fungi, Biosynthetic Pathway and Regulatory Mechanisms. Toxins.

[CR488] Wani MA, Sanjana K, Kumar DM, Lal DK (2010). GC-MS analysis reveals production of 2-phenylethanol from *Aspergillus niger* endophytic in rose. J Basic Microbiol.

[CR489] Wasil Z, Huhnert E, Simpson TJ, Cox RJ (2018). Oryzines A & B, maleidride congeners from *Aspergillus oryae* and their putative biosynthesis. J Fungi.

[CR490] Watts R, Dahiya J, Chanhary K, Tauro P (1988). Isolation of a new antifungal metabolite of *Trichoderma reesei*. Plant Soil.

[CR491] Wenke J, Anke H, Sterner O (1993). Pseurotin A and 8-O-demethylpseurotin A from *Aspergillus fumigatus* and their inhibitory activities on chitin synthase. Biosci Biotechnol Biochem.

[CR492] White EC (1940). Bactericidal filtrates from a mold culture. Science.

[CR493] White EC, Hill JH (1943). Studies on antibacterial products formed by molds. I. Aspergillic acid, a product of a strain of *Aspergillus flavus*. J Bacteriol.

[CR494] Wicklow DT, Kurata H, Ueno Y (1984). Adaptation in wild and domesticated yellow-green aspergilli. Toxigenic fungi—their toxins and health hazard.

[CR495] Wicklow DT, Cole RJ (1982). Tremorgenic indole metabolites and aflatoxins in sclerotia of *Aspergillus flavus*—an evolutionary perspective. Can J Bot.

[CR496] Wiemann P, Guo C-J, Palmer JM, Sekonyela R, Wang CCC, Keller NP (2013). Prototype of an intertwined secondary metabolite gene cluster. Proc Nat Acad Sci U S A.

[CR497] WU Zu-Jian, OUYANG Ming-An, SU Ren-Kuan, GUO Yue-Xiong (2008). Two New Cerebrosides and Anthraquinone Derivatives from the Marine FungusAspergillus niger. Chinese Journal of Chemistry.

[CR498] Wu ZC, Williams LJ, Danischefsky SJ (2000). A tree-step entry to the aspirochlorine family of antifungal agents. Angew Chem Int Ed.

[CR499] Xu X, Zhang X, Nong X, Wei X, Shuhua Q (2015). Oxindole alkaloids from the fungus *Penicillium commune* DFFSCS026 isolated from deep-sea-derived sediments. Tetrahedron.

[CR500] Yabuta T (1912). On kojic acid, a new organic acid produced by *Aspergillus oryzae*. J Coll Agr, Imp Univ Tokyo.

[CR501] Yabuta T (1922). A new organic acid (kojic acid) formed by *Aspergillus oryzae*. J Chem Soc.

[CR502] Yamada T, Hiratake J, Aikawa M, Suizu T, Saito Y, Kawato A, Suginami K, Oda J (1998). Cysteine protease inhibitors produced by the industrial koji mold, *Aspergillus oryzae* O-1018. Biosci Biotechnol Biochem.

[CR503] Yamaji K, Fukushi Y, Hashidoko Y, Yoshida T, Tahara S (1999). Characterization of antifungal metabolites produced by *Penicillium* species isolated from seeds of *Picea glehnii*. J Chem Ecol.

[CR504] Yang X-L, Awakara T, Wakimoto T, Abe I (2014). Three acyltetronic acid derivatives: noncanonical cryptic polyketides from *Aspergillus niger* identified by genome mining. ChemBioChem.

[CR505] Yang G, Sandjo L, Yun K, Leutou AS, Kim GD, Choi HD, Kang JS, Hong J, Son BW (2011). Flavusides A and B, antibacterial cerebrosides from the marine-derived fungus *Aspergillus flavus*. Chem Pharm Bull.

[CR506] Yassin MA, El-Rahim A, El-Samawaty MA, Moslem AA, Al-Arfaj AA (2015). Coffee bean myco-contaminants and oxalic acid producing *Aspergillus niger*. Ital J Food Sci.

[CR507] Ye Y, Minami A, Igarashi Y, Izumikawa M, Umemura M, Nagano N, Machida M, Kawahara T, Shin-ya K, Gomi K, Oikawa H (2016). Unveiling the biosynthetic pathway of the ribosomally synthesized and post-translationally modified peptide ustiloxin B in filamentous fungi. Angew Chem Int Ed.

[CR508] Ye YH, Zhu HL, Song YC, Liu JY, Tan RX (2005). Structural revision of aspernigrin A, reisolated from *Cladosporium herbarum* IFB-E002. J Nat Prod.

[CR509] Yoshimi A, Umemura M, Nagano N, Koike H, Machida M, Abe K (2016). Expression if *ustR* and the Golgi protease KexB are required for ustiloxin B biosynthesis in *Aspergillus oryzae*. AMB Express.

[CR510] Yoshizawa T, Tsuchiya Y, Morooka N, Sawada Y (1975). Malformin A_1_ as a mammalian toxicant from *Aspergillus niger*. Agric Biol Chem.

[CR511] Yu J, Tamura G, Takahashi N, Arima K (1967). Asperyellone, a new pigment of *Aspergillus awamori*. Agric Biol Chem.

[CR512] Zabala AO, Xu W, Chooi Y-H, Tang Y (2012). Charactrization of a silent azaphilone gene cluster from *Aspergillus niger* ATCC 1015 reveals a hydroxylation-mediate pyran-ring formation. Chem Biol.

[CR513] Zalesskaya MI (1950). Riboflavin formation in *Aspergillus flavus* mycelium grown in filtered cereal mashes. Mikrobiologiya.

[CR514] Zeilinger S, Gruber S, Bansal R, Mukherjee PK (2016). Secondary metabolism in *Trichoderma*—chemistry meets genomics. Fungal Biol Rev.

[CR515] Zeiliger S, Martin J-F, García-Estrada C (eds) (2015) Biosynthesis and molecular genetics of fungal secondary metabolites. vol. 2. Springer, New York

[CR516] Zeringue HJ, Shih BY, Moskos K, Grimm D (1999). Identification of the bright-greenish-yellow-fluorescence (BGY-F) compound on cotton lint associated with aflatoxin contamination in cottonseed. Phytochemistry.

[CR517] Zhang Y, Li X-M, Feng Y, Wang B-G (2010). Phenetyl-α-pyrone derivatives and cyclodipeptides from a marine algous endophytic fungus *Aspergillus niger* EN-13. Nat Prod Res.

[CR518] Zhang Y, Li X-M, Proksch P, Wang B-G (2007). Ergosterimide, a new natural Diels-Alder adduct of a steroid and maleimide in the fungus *Aspergillus niger*. Steroids.

[CR519] Zhang Y, Li X-M, Wang B-G (2007). Nigerasperones A-C, new monomeric and dimeric naphtho-γ-pyrones from marine alga-derived endophytic fungus *Aspergillus niger* EN-13. J Antibiot.

[CR520] Zhang Y, Li XM, Wang CY, Wang BG (2007). A new naphthoquinoneimide derivative from the marine algal-derived endophytic fungus *Aspergillus niger* EN-13. Chin Chem Lett.

[CR521] Zhang S, Monahan BJ, Tkacz JS, Scott B (2004). Indole-diterpene gene cluster from *Aspergillus flavus*. Appl Environ Microbiol.

[CR522] Zhang Y, Wang S, Li X, Cui CM, Feng C, Wang BG (2007). New sphingolipids with a previously unreported 9-methyl-C20-sphingosine moiety from a marine algous endophytic fungus *Aspergillus niger* EN-13. Lipids.

[CR523] Zhao G, Yao Y, Chen W, Cao X (2013). Comparison and analysis of the genomes of two *Aspergillus oryzae* strains. J Agric Food Chem.

[CR524] Zhao G, Yao Y, Hou L, Wang C, Cao X (2014a) Draft genome sequence of *Aspergillus oryzae* 100-8, an increased acid protease production strain. Genome Announc 2: e00548–14 10.1128/genomeA.00548-14 PMC4047454 24903875

[CR525] Zhao G, Yao Y, Hou L, Wang C, Cao X (2014). Comparison of the genomes and transcriptomes associated with the different protease secretions of *Aspergillus oryzae* 100-8 and 3.042. Biotechnol Lett.

[CR526] Zhao G, Yao Y, Wang C, Hou L, Cao X (2013). Comparative genomic analysis of *Aspergillus oryzae* strains 3.042 and RIB40 for soy sauce fermentation. Int J Food Microbiol.

[CR527] Zhao G, Yao W, Wang C, Hou L, Zeng B, Cao X (2012). Draft genome sequence of *Aspergillus oryzae* strain 3-042. Euk Cell.

[CR528] Zhou X, Fang W, Tan S, Lin X, Xun T, Yang B, Liu S, Liu Y (2016). Aspernigrins with anti-HIV-1 activities from the marine-derived fungus *Aspergillus niger* SCSIO Jcsw6F30. Bioorg Med Chem Lett.

[CR529] Zhuravleva OI, Kirichuk NN, Denisenko VA, Dmetrenok PS, Pivkin MV, Afiatullov SS (2016). New kipukasin from marine isolate of the fungus *Aspergillus flavus*. Chem Nat Comp.

